# Butorphanol decreased the median effective concentration of ropivacaine in ultrasound-guided interscalene brachial plexus block

**DOI:** 10.1371/journal.pone.0350613

**Published:** 2026-06-16

**Authors:** Hang Su, Juan Li, Jiping Zhang, Yiran Zhang, Faping Tu

**Affiliations:** 1 Department of Anesthesiology, Affiliated Hospital of North Sichuan Medical College, Nanchong, People’s Republic of China; 2 Department of Anesthesiology, Bazhong Central Hospital, Bazhong, People’s Republic of China; Para Federal University, BRAZIL

## Abstract

**Background:**

Combining butorphanol with local anesthesia can enhance the analgesic effect of local anesthesia and prolong the duration of action for nerve block anesthesia. In this study, we aimed to determine the effects of butorphanol on the median effective concentration (EC50) of ropivacaine in ultrasound-guided interscalene brachial plexus block (ISB).

**Methods:**

This is a randomized, double-blind, up-and-down sequential allocation study. Fifty patients undergoing arthroscopic shoulder surgery under general anesthesia were randomly assigned to the ropivacaine group or the butorphanol group in a 1:1 ratio. The former received 20 mL of ropivacaine for ultrasound-guided ISB, and the latter received a mixture containing 1 mg butorphanol and 20 mL ropivacaine. The concentration of ropivacaine for the first patient was 0.4%, and the ropivacaine concentration for the next patient was determined on the basis of the response of the previous patient to 0.4% ropivacaine using Dixon’s up-and-down sequential method with a ratio of 1:1.1.

**Results:**

Forty patients completed this study. The bootstrap EC50 of ropivacaine was significantly lower in the butorphanol group (0.24%, 95% CI: 0.23%–0.24%) than in the ropivacaine group (0.30%, 95% CI: 0.29%–0.31%). Compared with the ropivacaine group, the dosage of ropivacaine in the butorphanol group decreased significantly during the test. Postoperative visual analog scale scores at 8 h and 12 h were lower in the butorphanol group than in the ropivacaine group, and the need for salvage analgesia within 24 h after surgery was significantly lower in the butorphanol group.

**Conclusion:**

Butorphanol decreased the EC50 of ropivacaine required for ISB in patients undergoing arthroscopic shoulder surgery.

**Trial registration:**

It was registered on October 30th, 2022 (available at http://www.chictr.org.cn, identifier: ChiCTR2200065164).

## Introduction

Arthroscopic shoulder surgery offers the advantages of minimal trauma and allows for the observation and treatment of intra-articular lesions under clear vision; therefore, it has become the mainstay for diagnosing and treating shoulder joint diseases [1]. Continuous pressure irrigation with infusion fluid during shoulder arthroscopic surgery often leads to increased joint cavity pressure and local tissue edema [2–4], resulting in acute joint swelling and pain [5], which not only affect early postoperative recovery but also increase pain. Therefore, good postoperative analgesia is particularly important for rapid postoperative recovery of patients having undergone shoulder arthroscopic surgery. Notably, as the blood supply to the shoulder joint comes from the subclavian artery or axillary artery, tourniquets cannot be used in shoulder arthroscopic surgery. To reduce bleeding and maintain clear surgical field, controlled hypotension is often needed [6]. Nerve block combined with general anesthesia may reportedly reduce intraoperative stress response and maintain hemodynamic stability [7,8].

General anesthesia combined with brachial plexus block has seen increasing implementation in shoulder arthroscopic surgery [9,10]. Fredrickson et al. [5] believed that ultrasound-guided brachial plexus block could alleviate postoperative pain. According to previous research, higher concentrations of local anesthetic drugs are associated with more effective nerve block anesthesia [11]. However, in practical application, higher concentrations of local anesthetic drugs are also associated with increased incidence of adverse events, such as nerve damage and drug poisoning. Therefore, choosing the suitable concentration of local anesthetic drugs is of great significance. In recent years, the use of adjuvants in combination with local anesthetics for nerve block has increased to reduce the concentration of local anesthetics in nerve block anesthesia and enhance their analgesic effect. Desai et al [12]. believed that both dexmedetomidine + local anesthetics and dexamethasone + local anesthetics could prolong the analgesic time for nerve block anesthesia. In addition, giving ropivacaine in combination with opioid drugs can also enhance the analgesic effect and reduce the dosage of local anesthetics [13].

Butorphanol tartrate is an artificially synthesized opioid receptor agonist-antagonist. Recent studies have shown that adding butorphanol to ropivacaine for nerve block can shorten the onset time of nerve block, enhance the analgesic effect, and prolong the analgesic time [14]. The present study aimed to use butorphanol + ropivacaine for ultrasound-guided interscalene brachial plexus block (ISB) and explored the effect of local administration of butorphanol on the median effective concentration (EC50) of ropivacaine for ultrasound-guided ISB.

### Materials and methods

#### Ethical approval

This was a prospective, randomized, double-blind, and up-and-down sequential allocation trial. The study protocol was approved by the Institutional Ethics Committee of the Suining Central Hospital (S4 Fig,approval no: LLSLH20220029) and registered in the Chinese Clinical Trials Registry in October 30th, 2022 (available at http://www.chictr.org.cn, identifier: ChiCTR2200065164), all methods were carried out in accordance with relevant guidelines and regulations. Participants were recruited between June 13, 2022, and December 31, 2022, from patients at the Suining Central Hospital.The study protocol, including the design, eligibility criteria, primary and secondary outcomes, and statistical analysis plan, was finalized prior to the enrollment of the first participant. The delay in registration was primarily due to administrative oversight during the early stage of the study initiation. The authors confirm that all ongoing and related trials for this drug/intervention are registered and all participants provided their written informed consent prior to participation in the study.

#### Participants

In this study, we enrolled 50 patients who underwent arthroscopic shoulder surgery under general anesthesia. The inclusion criteria were 20–65 years of age, American Society of Anesthesiologists (ASA) physical status I–II, and body mass index (BMI) of 18–28 kg/m^2^. We excluded patients with serious cardiopulmonary disease, abnormal liver and kidney function, skin infection at the site of nerve block, coagulation dysfunction, drug abuse,and known allergy to general anesthetics and research drugs. Furthermore, patients who did not cooperate during the trial, experienced adverse events (e.g., major bleeding and cardiac arrest), had insufficient postoperative follow-up time, or had incomplete data were considered for withdrawal.

#### Randomization and blinding

Using a computer-generated random number sequence, the eligible patients were randomly divided into ropivacaine and butorphanol groups. Opaque sealed envelopes were used to conceal the allocation details. A research nurse prepared the study solutions in identical 20-mL syringes. The study solution for all patients was diluted with 0.9% normal saline to a final volume of 20 mL. For all patients in the butorphanol group, the quantity of butorphanol was 1 mg, and the final volume of butorphanol + ropivacaine was 20 mL. The concentration of ropivacaine for the first patient was 0.4% in both groups. The ropivacaine concentration for the next patient was determined on the basis of the response of the previous patient. All patients, surgeons, and other investigators were blinded to the group allocation.

#### Anesthesia

All patients underwent routine 8-h fasting, and no preoperative medication was administered. After entering the operating room, electrocardiogram, pulse oximetry, systolic blood pressure, diastolic blood pressure, non-invasive mean arterial blood pressure (MAP), and heart rate (HR) were monitored. A peripheral venous channel was established and compound sodium chloride injection (6–8 mL/kg^-1^·h^-1^) was administered.

All patients were placed in a supine position, with their upper limbs on the surgical side closely attached to the trunk and the head slightly tilted to the opposite side. First, the location of the intermuscular sulcus was identified under ultrasound to determine the precise location of the brachial plexus nerve. The linear array probe was placed at the level of the cricoid cartilage and gradually advanced toward the outer edge of the sternocleidomastoid muscle. Several low-echoic structures were noted between the anterior and middle scalene muscles, and these structures appeared to be arranged in a string of beads. This was identified as the location of the brachial plexus (S1 Fig). The probe was slid up and down to identify the ideal section for puncture, and before puncture, it was differentiated from blood vessels using ultrasound color mode to avoid puncture injury and prevent the local anesthetic from erroneously entering the blood vessels. After identifying the precise location of the brachial plexus nerve, the intended puncture site was disinfected, and a 50-mm puncture needle was used to insert the needle at a distance of 1–1.5 cm outside the ultrasound probe. The puncture needle was advanced at a 45° angle to the skin, and an in-plane puncture technique was used (S2 Fig). When the tip of the puncture needle reached the vicinity of the nerve and there was no blood, gas, or cerebrospinal fluid in the syringe, the experimental drug was injected into the space between the two scalene muscles to completely surround the brachial plexus nerve with local anesthetic solution (S3 Fig). If the puncture needle position needs to be adjusted, the syringe must be drawn back again without blood before injection. Within 30 min of brachial plexus block injection, acupuncture using a 1-mL syringe needle was used every 5 min to evaluate skin pain in areas innervated by the musculocutaneous nerve, radial nerve, median nerve, ulnar nerve, and axillary nerve. Based on the degree of pain reduction, the level of pain was interpreted as follows: level I indicated normal or slightly reduced pain, level II indicated significantly reduced pain, and level III indicated no pain. Negative reactions are defined as Grade III (no pain) in all five innervated areas, whereas positive reactions are defined as Grade I–II (Grade I: normal or slightly reduced pain; Grade II: significantly reduced pain) in any part of the five innervated areas. When a negative reaction was noted in a patient, the concentration of ropivacaine was reduced by one level in the next patient, and when a positive reaction was noted in a patient, the concentration of ropivacaine was increased by one level in the next patient. The trial was terminated when seven negative *vs*. positive or positive *vs*. negative crossover points were obtained.

After completing the brachial plexus block and observing for 30 min (EC50 test completed), general anesthesia was induced. The patient was given oxygen via an oxygen mask for 3 min, and the oxygen flow rate was 6 L/min. Then, 0.4 µg/kg sufentanil, 2 mg/kg propofol, and 0.6 mg/kg rocuronium were administered intravenously to induce an appropriate depth of anesthesia with complete muscle relaxation, and mechanical ventilation was started through tracheal intubation. Respiratory parameters (tidal volume 6–8 mL/kg, respiratory rate 10–15 breaths/min) were adjusted to maintain the end-expiratory carbon dioxide partial pressure (PETCO_2_) at 35–45 mmHg. Positive end-expiratory pressure was used where necessary. Intraoperative infusion of propofol and remifentanil was used to maintain anesthesia, with an electroencephalogram bispectral index value of 40–60. The patient’s body temperature was maintained between 36℃ and 37℃. Hypotension was defined as a > 20% drop in the MAP from the baseline and was treated with intravenous injection of 6-mg ephedrine or 20% μg deoxyepinephrine. When an increase of >20% relative to the baseline was noted in the MAP, 5-mg urapidil was intravenously administered. When the HR declined to <50 beats/min, atropine 0.3–0.5 mg was intravenously administered, and when it exceeded >130 beats/min, esmolol 10–20 mg was intravenously administered. Repeated administration may be necessary.

If symptoms indicative of local anesthetic poisoning, such as nausea, vomiting, increased breathing rate and HR, delirium, and convulsions, occurred during the nerve block, the use of the local anesthetic was immediately stopped, the patient was given oxygen inhalation, and respiratory tract patency was maintained. During tachycardia, esmolol was administered to normalize the HR. Ventricular arrhythmia was treated with amiodarone. If the patient started convulsing, propofol was administered, and if necessary, intralipid was used. If circulatory suppression was noted, intravenous infusion or the use of vasoactive drugs was accelerated. In the event of respiratory suppression, mask pressure was used to provide oxygen, and emergency tracheal intubation was performed if necessary. If allergic reactions occurred, corresponding treatments were carried out according to the grading of allergic reactions.

Remifentanil was stopped 30 min before the end of the surgery, and intravenous sufentanil was administered at 0.2 μg/kg for pain relief. Propofol was stopped at the end of suturing. After the surgery, we waited for the patient to wake up, recover spontaneous breathing and show stable circulation, and then, the patient was sent to the recovery room for observation after extubation and anesthesia recovery. After the surgery, celecoxib (0.2 g. bid) was administered orally.

#### Statistical analysis

The EC50 of ropivacaine was determined using Dixon’s up-and-down sequential allocation method [15]. The data of sequential allocation method has two major characteristics: non independence and unknown distribution, so conventional sample size calculation methods are not suitable for sequential allocation methods. A simulation study shows that including at least 20–40 patients will provide a stable estimate of the target dose for most scenarios [16]. With a sample size of 22 cases, Tian et al [17]. used the sequential method to identify the EC50 of ropivacaine for brachial plexus block. Considering that the improved sequential method terminated the experiment at 6–7 crossovers, the final sample size was determined to be 25 patients in each group. We used the SPSS software (version 25; SPSS Inc., IBM Corp, Armonk, USA) to process the data. All data were subjected to normality tests, and normally distributed variables were described as mean ± standard deviation. The patient’s age, BMI, and visual analog scale (VAS) score were statistically analyzed using independent sample t-tests. Count data, such as patient’s sex and number of additional postoperative analgesics, were tested using chi-square tests or Fisher’s exact test. Multiple comparisons were adjusted using the Benjamini–Hochberg (BH) method. Given the nonindependent structure and unknown distributional properties of responses obtained from the up-and-down sequential allocation design, traditional parametric approaches such as probit regression may yield biased estimates. Therefore, EC50 was estimated using isotonic regression implemented via the pooled-adjacent-violators algorithm (PAVA), in accordance with the recommendations of Pace and Stylianou. Bootstrap resampling was applied to derive the mean EC50 and corresponding 95% confidence intervals (CIs), as well as to evaluate the between-group difference (contrast) in EC50. A sensitivity analysis was performed by re-including the excluded samples. Statistical significance was defined as a two-sided P value <0.05.

## Results

### Patient

**Fig 1:**
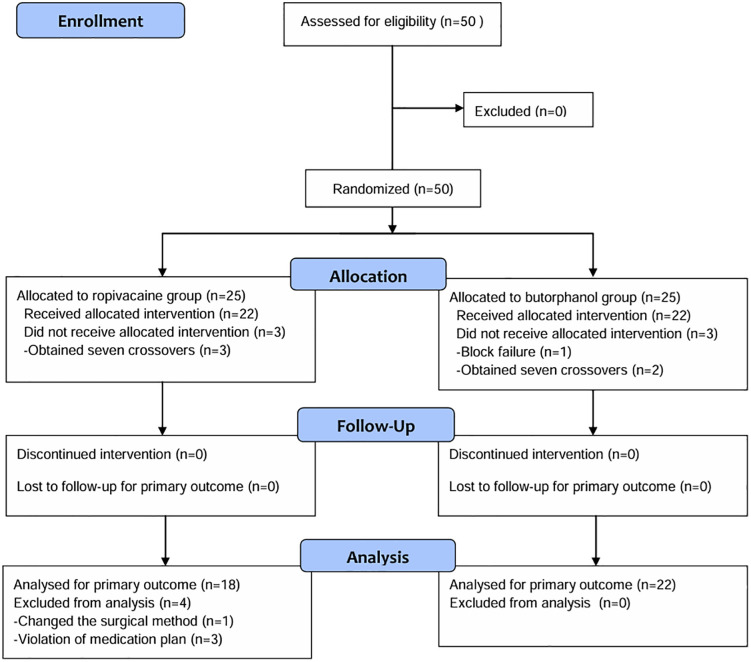
Flowchart of the study.

**Fig 2:**
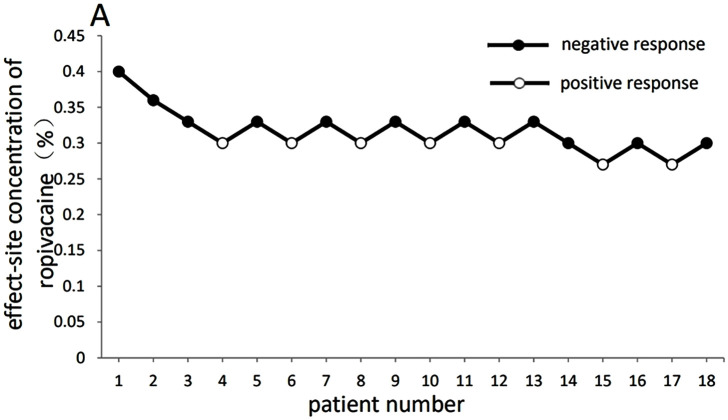
Dixon up-and-down plots for the ropivacaine group.

**Fig 3:**
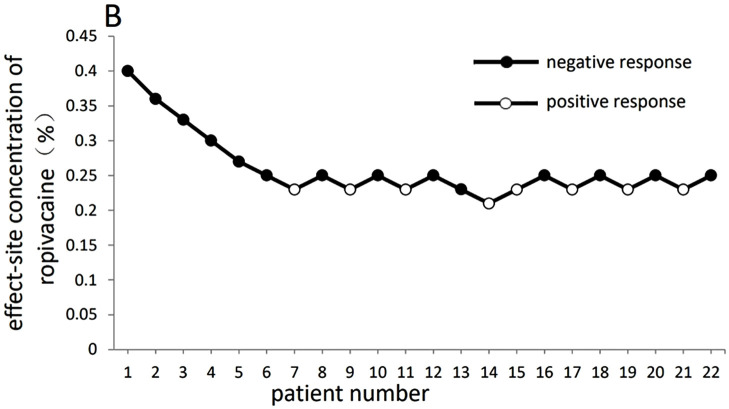
Dixon up-and-down plots for the butorphanol group.

Fifty patients (25 in each group) were initially included in this trial. In the ropivacaine group, 3 patients were excluded for violating the postoperative medication plan, and 1 patient was excluded for a change in the surgical method. In the butorphanol group, 1 patient was excluded for failing to complete the ultrasound-guided interscalene brachial plexus block (ISB) procedure. In addition, 3 patients in the ropivacaine group and 2 in the butorphanol group were excluded from the study after seven crossover points were obtained.Among the excluded patients, only 4 cases (all in ropivacaine group) had available data. Finally, 40 patients were included in the statistical analysis, with 18 in the ropivacaine group and 22 in the butorphanol group (Fig 1). Figs 2 and 3 show the Dixon up-and-down plots for each group. Patient characteristics did not significantly differ between the two groups. Compared with the ropivacaine group, the dosage of ropivacaine was significantly reduced in the butorphanol group (*P* < 0.05; Table 1).

**Table 1:** Patient characteristics and medications.

	Ropivacaine group (n = 18)	Butorphanol group (n = 22)	*P* value
Age (years)	51.94 ± 7.54	48.68 ± 9.16	0.23
BMI (kg/m^2^)	24.19 ± 2.63	23.22 ± 2.67	0.26
Sex (male/female)	8/10	10/12	0.95
Operation time (min)	124.72 ± 45.78	120.91 ± 52.57	0.81
Sufentanil (µg)	35.28 ± 8.13	36.25 ± 5.86	0.67
Remifentanil (µg)	698.44 ± 197.60	720.45 ± 256.35	0.77
Ropivacaine (mg)	63.11 ± 6.22	51.90 ± 9.43	<0.05

**Notes:** Data are presented as mean ± SD or numbers. Independent sample t-test and Fisher’s exact test was used.

**Abbreviations:** BMI, body mass index.

### Adverse reactions

After brachial plexus block and before the operation, adverse reactions occurred in 6 patients in the ropivacaine group and in only 1 patient in the butorphanol group; the incidence of adverse reactions was significantly lower in the butorphanol group than in the ropivacaine group (*P* < 0.05; Table 2). The number of patients who experienced adverse reactions during the operation in ropivacaine and butorphanol groups was 5 and 4 respectively, which did not statistically significantly differ between the two groups (*P* > 0.05; Table 2).

**Table 2:** Adverse reactions for the two groups.

	Ropivacaine group (n = 18)	Butorphanol group (n = 22)	*P* value
Preoperative adverse reactions	6 (33.33%)	1 (4.55%)	0.03
Vomiting	1(5.56%)	0 (0%)	0.45
Perioral numbness	3 (16.67%)	0 (0%)	0.08
Dyspnea	2 (11.11%)	1 (4.55%)	0.58
Intraoperative adverse reactions	5 (27.78%)	4 (18.18%)	0.70
Bradycardia	4 (22.22%)	4 (18.18%)	1.00
Hypotension	1 (5.56%)	0 (0%)	0.45

**Notes:** Data are presented as numbers or percentages. Fisher’s exact test was used.

### Postoperative MAP,HR, and pain assessment

**Fig 4:**
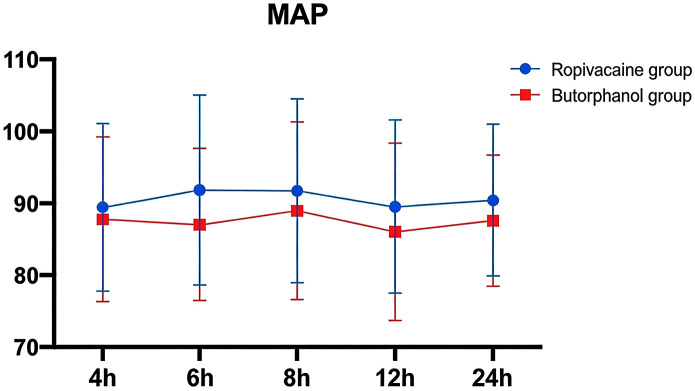
Dynamic variations in MAP.

**Fig 5:**
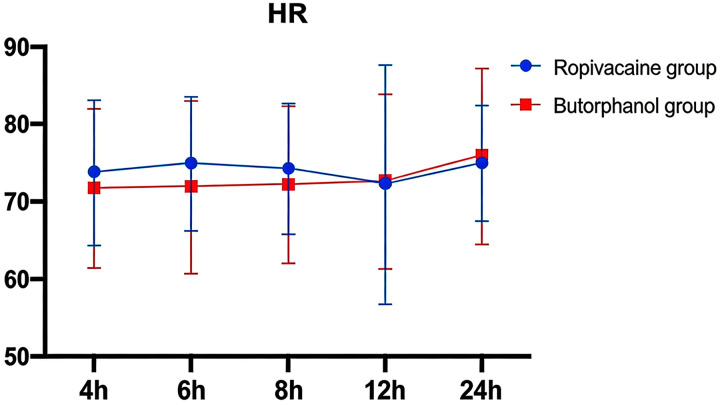
Dynamic variations in HR.

**Fig 6:**
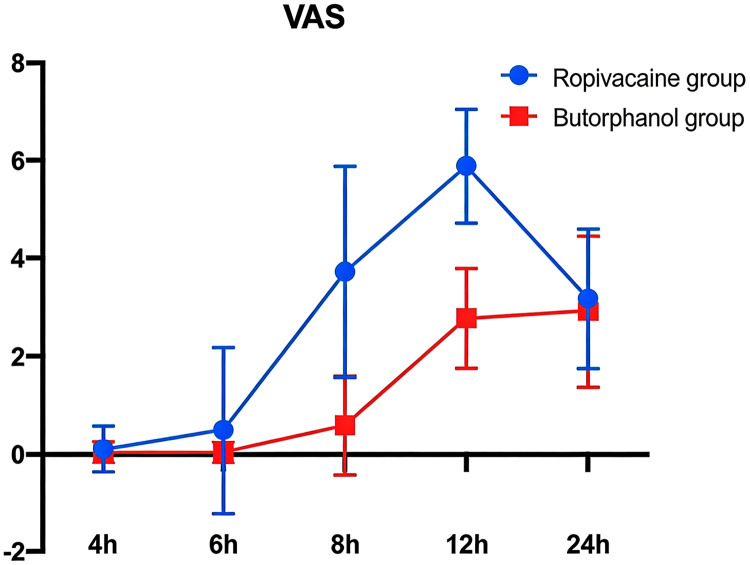
Dynamic variations in VAS.

MAP and HR values did not significantly differ between the two groups at different time points. The VAS scores at 8 h and 12 h postoperatively were significantly lower and the number of patients receiving rescue analgesia at 24 h postoperatively was significantly smaller in the butorphanol group (*P* < 0.05; Table 3. Figs 4–6 show dynamic variations in MAP, HR, and VAS over time in both groups postoperatively.

**Table 3:** Postoperative MAP, HR,and pain assessment for the two groups.

	Ropivacaine group (n = 18)	Butorphanol group (n = 22)	*P* value
Postoperative MAP			
4 h	89.44 ± 11.66	87.77 ± 11.46	0.65
6 h	91.83 ± 13.20	87.05 ± 10.62	0.21
8 h	91.72 ± 12.76	88.95 ± 12.36	0.49
12 h	89.56 ± 12.03	86.09 ± 12.31	0.38
24 h	90.44 ± 10.58	87.59 ± 9.18	0.37
Postoperative HR			
4 h	73.72 ± 9.38	71.68 ± 10.27	0.52
6 h	74.83 ± 8.65	71.86 ± 11.15	0.36
8 h	74.17 ± 8.47	72.14 ± 10.18	0.50
12 h	72.22 ± 15.44	72.59 ± 11.27	0.93
24 h	74.89 ± 7.52	75.86 ± 11.41	0.76
Postoperative VAS			
4 h	0.11 ± 0.47	0.05 ± 0.21	0.56
6 h	0.5 ± 1.69	0.05 ± 0.21	0.27
8 h	3.72 ± 2.16	0.59 ± 1.01	<0.05
12 h	5.89 ± 1.18	2.77 ± 1.02	<0.05
24 h	3.17 ± 1.42	2.91 ± 1.54	0.59
Rescue analgesia	14(77.8%)	3(13.6%)	<0.05

**Notes:** Data are presented as mean ± SD, or number (percentage of patients). Independent sample t-test and Fisher’s exact test was used.

**Abbreviations:** HR, heart rate; MAP, mean arterial pressure; VAS, visual analog scale.

### Butorphanol reduced the EC50 of ropivacaine

**Fig 7:**
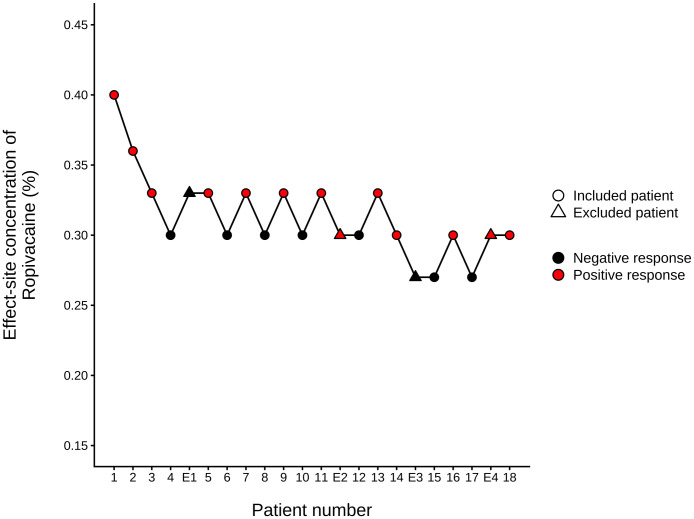
Dixon up-and-down plots for the ropivacaine group after re-including the excluded samples. **Abbreviations:**E1-E4 refer to the 4 excluded patients.

Based on bootstrap estimation following the PAVA method, the EC50 of ropivacaine was lower in the butorphanol group compared with the ropivacaine group. The bootstrap EC50 was 0.24% (95% CI: 0.23–0.24%) in the butorphanol group and 0.30% (95% CI: 0.29–0.30%) in the ropivacaine group. The confidence intervals did not overlap, indicating a statistically significant difference between the two groups (P < 0.05) (Table 4).After re-including the excluded samples and conducting a sensitivity analysis, the results were essentially identical to those of the primary analysis. Dixon up-and-down plots for the ropivacaine group after re-including excluded samples are presented in Fig 7. As presented in Table 5, the PAVA-derived EC50 was 0.30% for the ropivacaine group and 0.24.% for the butorphanol group. The bootstrap EC50 estimates were 0.30% and 0.24%, respectively. The 95% CI remained very similar (0.29%–0.32% in the ropivacaine group and 0.23%–0.24% in the butorphanol group), confirming that the main findings were robust to re-inclusion of these samples(Table 5).To accurately compare the differences before and after sensitivity analysis, we present the data rounded to three decimal places.

**Table 4:** EC50 values of Ropivacaine and Butorphanol group.

	Ropivacaine group (n = 18)	Butorphanol group (n = 22)	*P* value
PAVA EC50	0.31%(0.306%)	0.24%(0.239%)	*P* < 0.05
Bootstrap EC50	0.30%(0.305%)	0.24%(0.238%)	*P* < 0.05
95% CI	0.29%–0.32% (0.291%–0.315%)	0.23%–0.24% (0.233%–0.240%)	*P* < 0.05
Bootstrap 95% CI	0.29%–0.31% (0.289%–0.313%)	0.23%–0.24% (0.225%–0.240%)	*P* < 0.05

**Notes:** Data are presented as percentage.

**Abbreviations:** PAVA,Pooled Adjacent Violators Algorithm.

**Table 5:** EC50 values of Ropivacaine and Butorphanol group after re-including the excluded samples.

	Ropivacaine group (n = 22)	Butorphanol group (n = 22)	*P* value
PAVA EC50	0.30%(0.300%)	0.24%(0.239%)	*P* < 0.05
Bootstrap EC50	0.30(0.302%)	0.24(0.238%)	*P* < 0.05
95% CI	0.29%–0.32% (0.289%–0.319%)	0.23%−0.24% (0.233%–0.240%)	*P* < 0.05
Bootstrap 95% CI	0.29%–0.31% (0.288%–0.314%)	0.23%–0.24% (0.225%–0.240%)	*P* < 0.05

**Notes:** Data are presented as percentage.

**Abbreviations:** PAVA,Pooled Adjacent Violators Algorithm.

## Discussion

We herein found that butorphanol reduced the EC50 of ropivacaine for ISB in patients undergoing arthroscopic shoulder surgery. The EC50 of ropivacaine was 0.30% in the ropivacaine group and 0.24% in the butorphanol group.

The distribution of brachial plexus nerves in the intermuscular sulcus is relatively concentrated and shallow, and local blockade with ropivacaine can achieve precise analgesic effects [18]. Ropivacaine is the preferred local anesthetic for brachial plexus block [19]. Li et al. [20] recommend 0.4% ropivacaine as the optimal concentration for ultrasound-guided ropivacaine brachial plexus block. Therefore, we herein used 0.4% ropivacaine as the initial concentration, and the ratio of adjacent concentrations varied at 1:1.1. In this experiment, the EC50 of ropivacaine in the ropivacaine group was 0.30%, which is within the recommended concentration range by most researchers. Tian et al. [17] reported 0.21% as the EC50 of ropivacaine for intermuscular groove brachial plexus block. The reason for the difference in our results may be that we evaluated the blocking effect within 30 min of brachial plexus block injection, whereas they performed their evaluation at the time of awakening after the surgery, which takes much longer than 30 min.

Shaik et al. [21] identified the presence of opioid receptors on the surface of peripheral neurons and immune cells. Animal experiments have also confirmed that fentanyl and sufentanil can reduce the action potential amplitude of peripheral nerves, indicating that opioid drugs have anesthetic effects on peripheral nerves [22]. Butorphanol is an artificially synthesized opioid receptor agonist-antagonist that can directly act on opioid receptors in peripheral nerves. In addition, it passes through the peripheral nerve membrane and is bound by opioid-binding proteins before being transported to the dorsal horn of the spinal cord to exert analgesic effects [23]. Abboud et al. [24] found that epidural administration of butorphanol combined with 2% lidocaine resulted in a faster onset of spinal anesthesia, and the duration and efficacy of pain relief increased with increasing dosage. When butorphanol + ropivacaine is used for local anesthesia, it enhances the analgesic effect [25] and is beneficial for rapid postoperative recovery [26]. Butorphanol can reportedly prolong the duration of ropivacaine brachial plexus block and enhance its analgesic effect [27]. In the present study, we chose 1 mg of butorphanol for the experiment, and we found that the EC50 of ropivacaine was 0.30% in the ropivacaine group and 0.24% in the butorphanol group, indicating that supplementing butorphanol can reduce the EC50 of ropivacaine for ultrasound-guided ISB.

In the observation of VAS scores at various time points within 24 h postoperatively, we found that the VAS scores at 8 h and 12 h postoperatively were significantly lower in the butorphanol group than in the ropivacaine group; however, VAS scores at 4–6 h and 24 h postoperatively did not significantly differ between the two groups When the concentration of ropivacaine hydrochloride used in this study was between 0.21% and 0.4%, the analgesic time was 4–6 h. The EC50 of ropivacaine in the ropivacaine group was 0.31%, and thus, the VAS score of ropivacaine group patients significantly increased at 6–12 h after the surgery; however, the VAS score in the butorphanol group did not increase significantly between 6–12 h, which may be related to the prolonged duration of analgesia with butorphanol. The lack of significant differences in VAS scores between the two patient groups at 24 h after the surgery can be attributed to the disappearance of analgesic effects in both groups, resulting in similarly high VAS scores. Our findings suggest that butorphanol prolongs the brachial plexus block time, which is consistent with the findings of Ding et al. [28] The prolonged analgesic effect may be related to butorphanol’s stimulation of the κ receptors in spinal dorsal horn. In addition, the prolonged analgesic effect may also be related to the release of endogenous opioid peptides triggered by the absorption of butorphanol into the bloodstream by peripheral blood vessels, resulting in analgesic effects [29^,^^30^].

In this study, the amount of ropivacaine used was lesser in the butorphanol group than in the ropivacaine group (51.90 ± 9. 43 mg *vs*. 63.11 ± 6.22 mg, *P* < 0.05), and the former had significantly fewer patients receiving postoperative rescue analgesia, indicating that supplementing butorphanol to ropivacaine for nerve block can reduce the amount of local anesthetic drugs required and significantly enhance the postoperative analgesic effect. There was no between-group difference in intraoperative opioid dosage and postoperative MAP and HR, which may be attributed to the brachial plexus block blocking the transmission of harmful stimuli to the surgical site. Both patient groups may only need appropriate sedation during the surgery. In this study, the incidence of adverse reactions to nerve block was significantly lower in the butorphanol group than in the ropivacaine group, which may be attributed to the lower concentration of ropivacaine needed in the butorphanol group. Based on our findings, the combination of butorphanol and lower concentrations of ropivacaine can achieve comparable analgesic effects as higher concentrations of ropivacaine. The findings give us a reason to believe that using butorphanol as an adjuvant for ropivacaine brachial plexus block can achieve ideal analgesic effects with less ropivacaine, and this also reduces the adverse reactions caused by local anesthetics because of the lower concentrations required.

The current study has some limitations. First, we included patients aged 20–65 years with ASA grades I–II, and thus, the findings are not extrapolatable to elderly patients and patients with higher ASA grades. Second, the dosage of butorphanol was determined based on previous experimental results, and further research is needed to determine whether it is the optimal dosage for adjuvant therapy of brachial plexus block. Finally, our analgesic and observation goals mainly focused on the first 24 h after surgery, and a prolonged observation period will be used in future studies.

## Conclusion

Butorphanol decreased the EC50 of ropivacaine required for ISB in patients undergoing arthroscopic shoulder surgery.

## Supporting information

S1 Fig Ultrasound image of the brachial plexus. **Abbreviations:** ASM = anterior scalene muscle, MSM = middle scalene muscle, C5, C6, C7 brachial plexus.
(TIF)

S2 Fig Ultrasound-guided brachial plexus puncture image. (TIF)

S3 Fig Ultrasound image of liquid encapsulation around the brachial plexus. (TIF)

S4 Fig Approved file of Ethical Committee. (JPG)

S1 File CONSORT 2025 checklist. (DOCX)

S2 File Study protocol original version. (DOCX)

S3 File Study protocol. (DOCX)

S4 File Original data part 1. (ZIP)

S5 File Original data part 2. (ZIP)

S6 File Original data part 3. (ZIP)

S7 File Original data part 4. (ZIP)

S8 File English translation of the original data. (DOCX)

S9 File English translation of the original ethics approval document. (PDF)

## Abbreviations

EC50: median effective concentration
ISB: interscalene brachial plexus block
CI: confidence interval
ASA: American Society of Anesthesiologists
BMI: body mass index
MAP: arterial blood pressure
HR: heart rate
PETCO_2_: end-expiratory carbon dioxide partial pressure
VAS: visual analog scale
