# Supplementary figures and images for: Butorphanol decreased the median effective concentration of ropivacaine in ultrasound-guided interscalene brachial plexus block
Source: PLoS One. 2026 Jun 16;21(6):e0350613. doi: 10.1371/journal.pone.0350613 (PMC13271508; doi:10.1371/journal.pone.0350613)

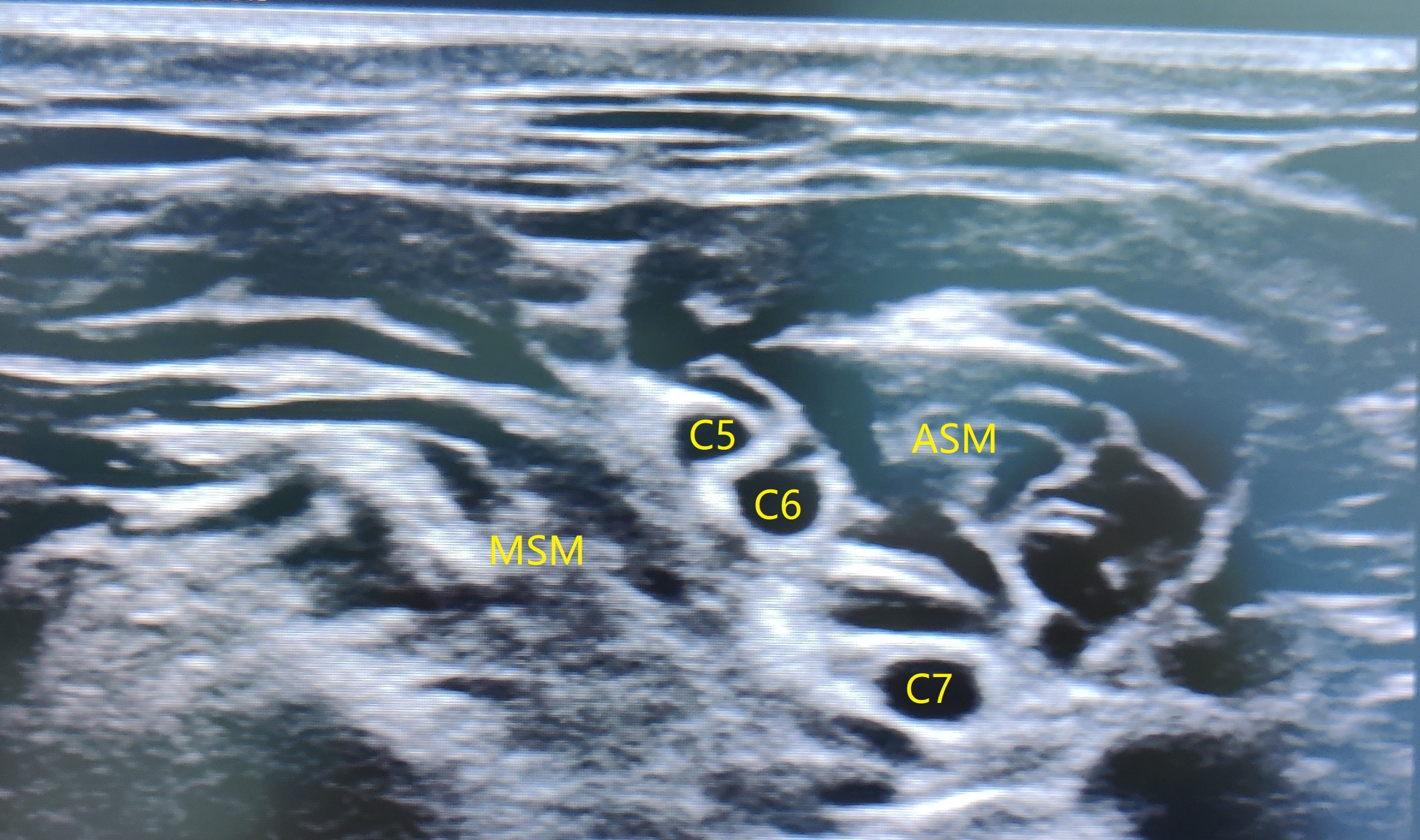

Supplement: S1 Fig — Abbreviations: ASM = anterior scalene muscle, MSM = middle scalene muscle, C5, C6, C7 brachial plexus. (TIF) [file pone.0350613.s001.tif]

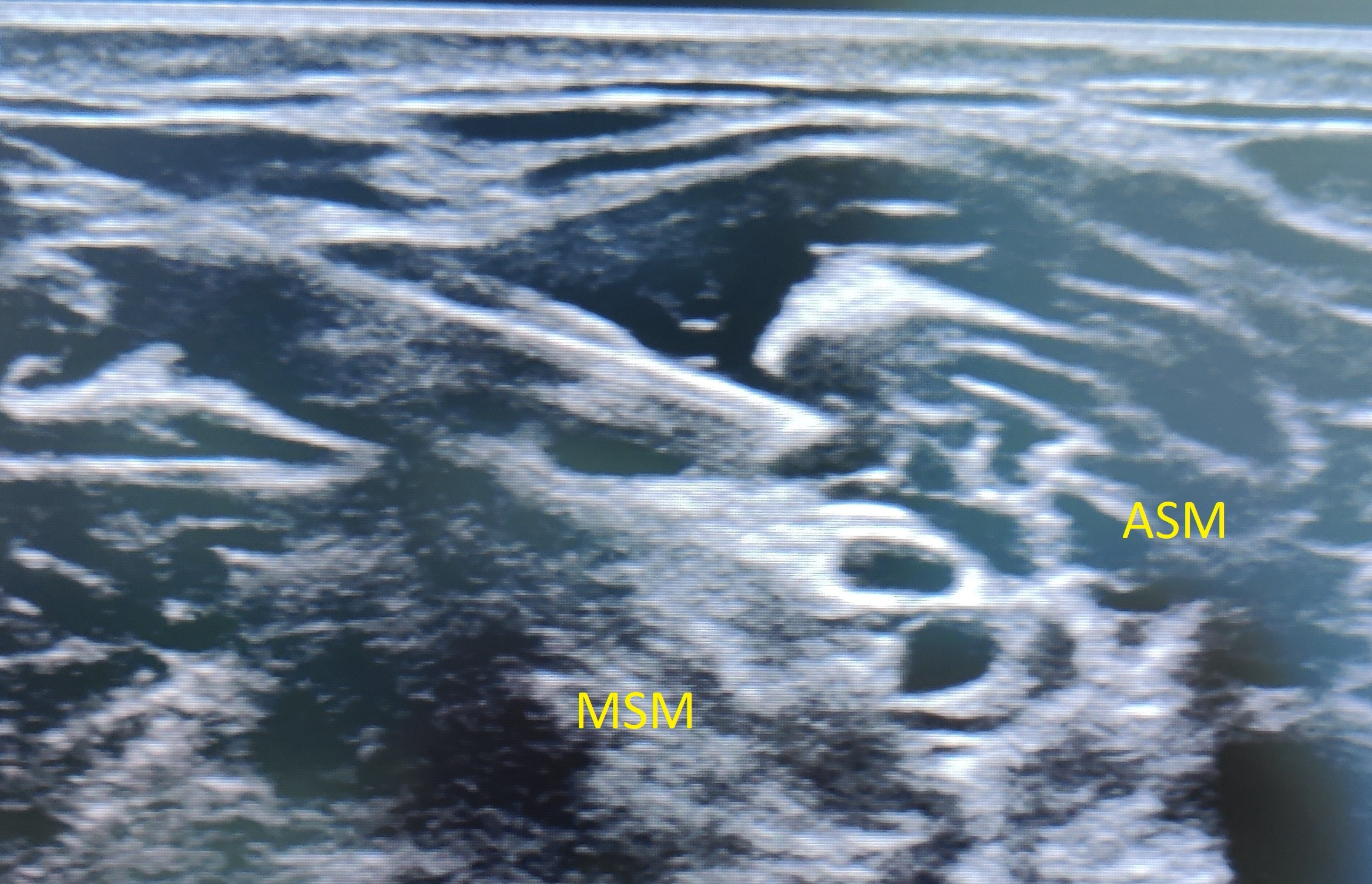

Supplement: S2 Fig — (TIF) [file pone.0350613.s002.tif]

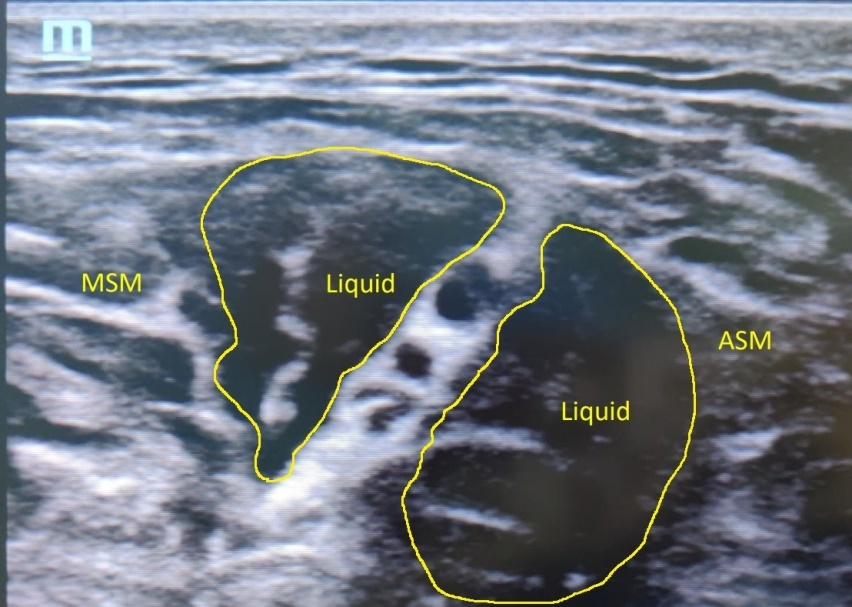

Supplement: S3 Fig — (TIF) [file pone.0350613.s003.tif]

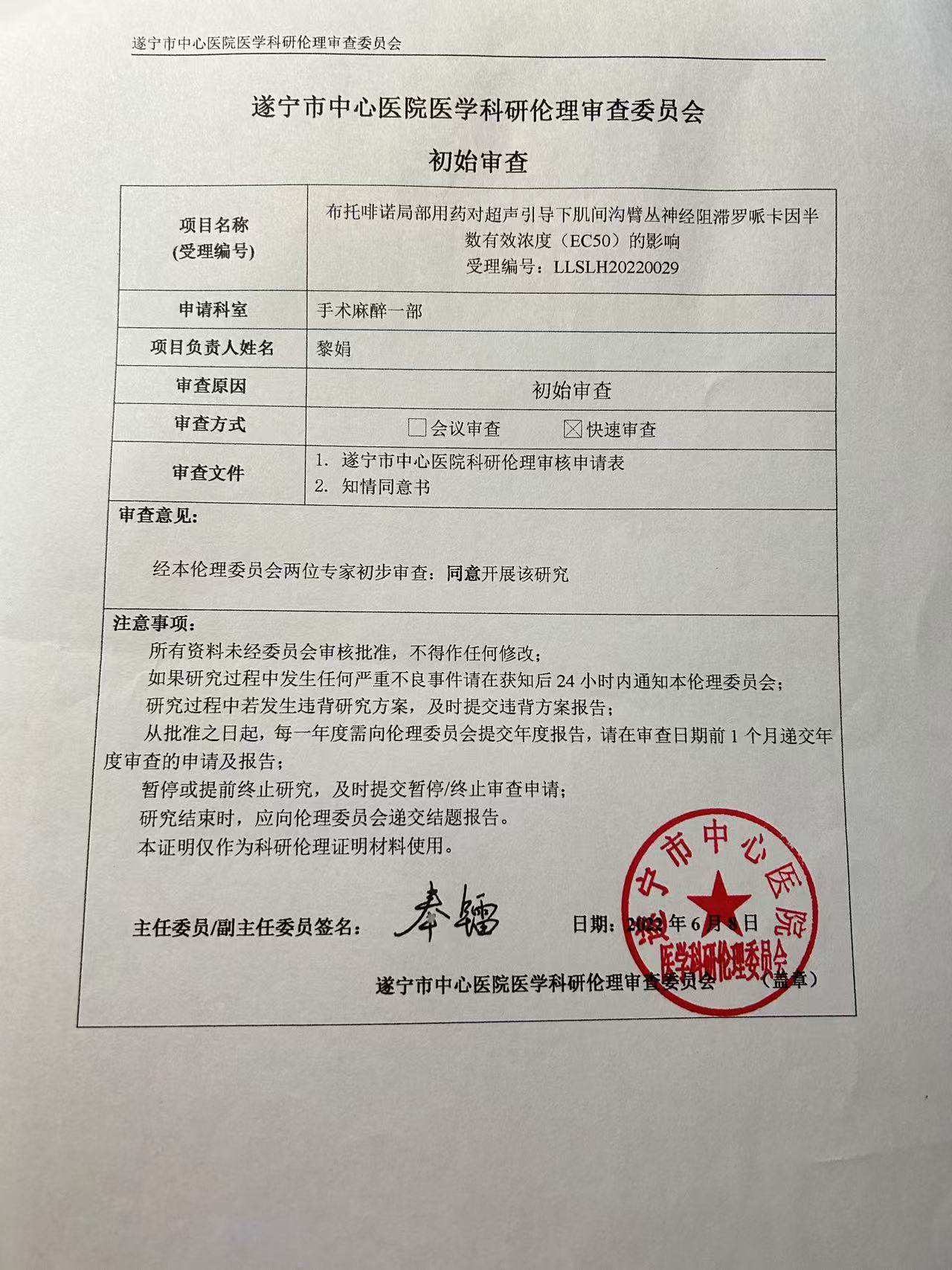

Supplement: S4 Fig — (JPG) [file pone.0350613.s004.jpg]

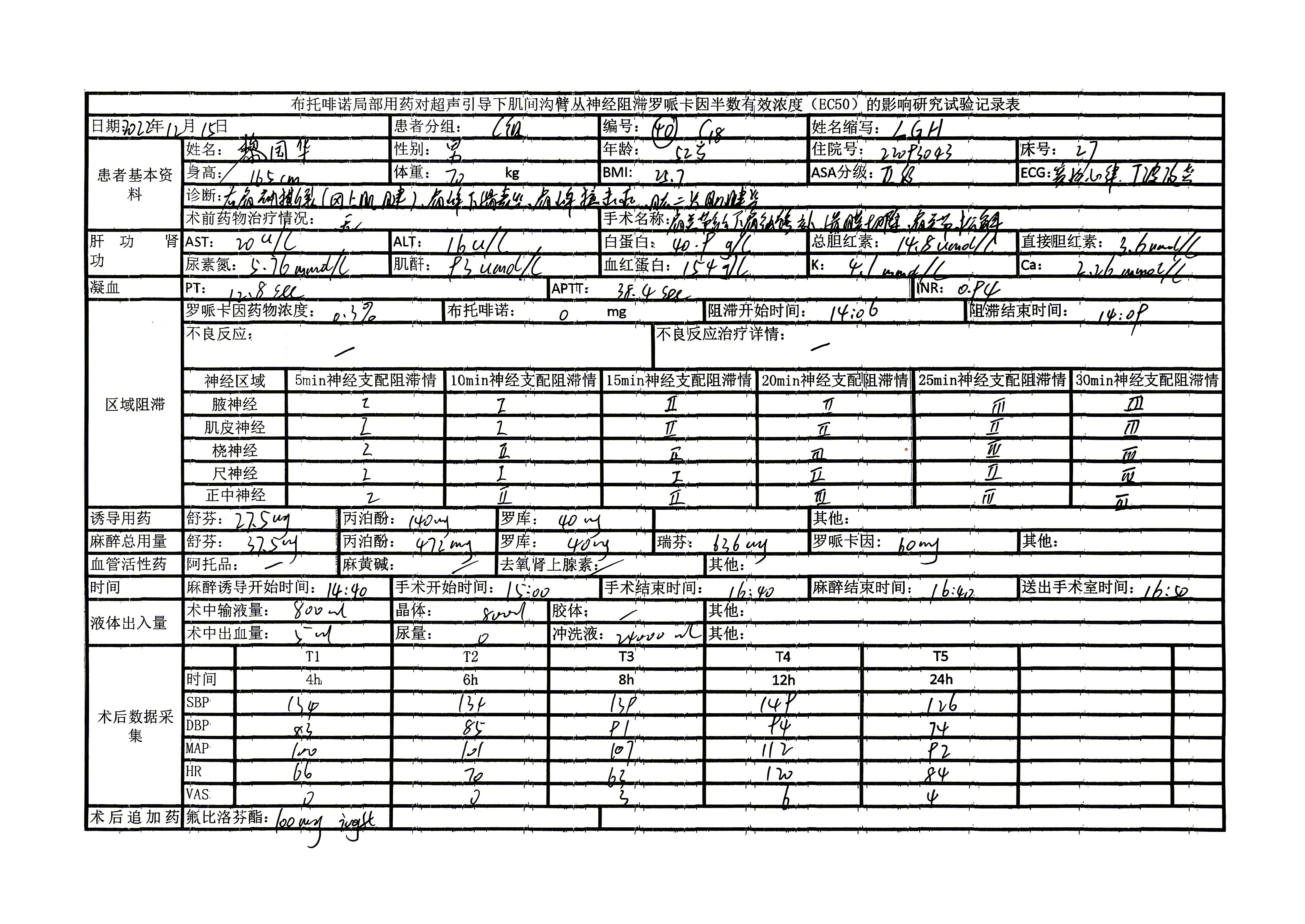

Supplement: S4 File — (ZIP) [file pone.0350613.s008.zip › 001.jpg]

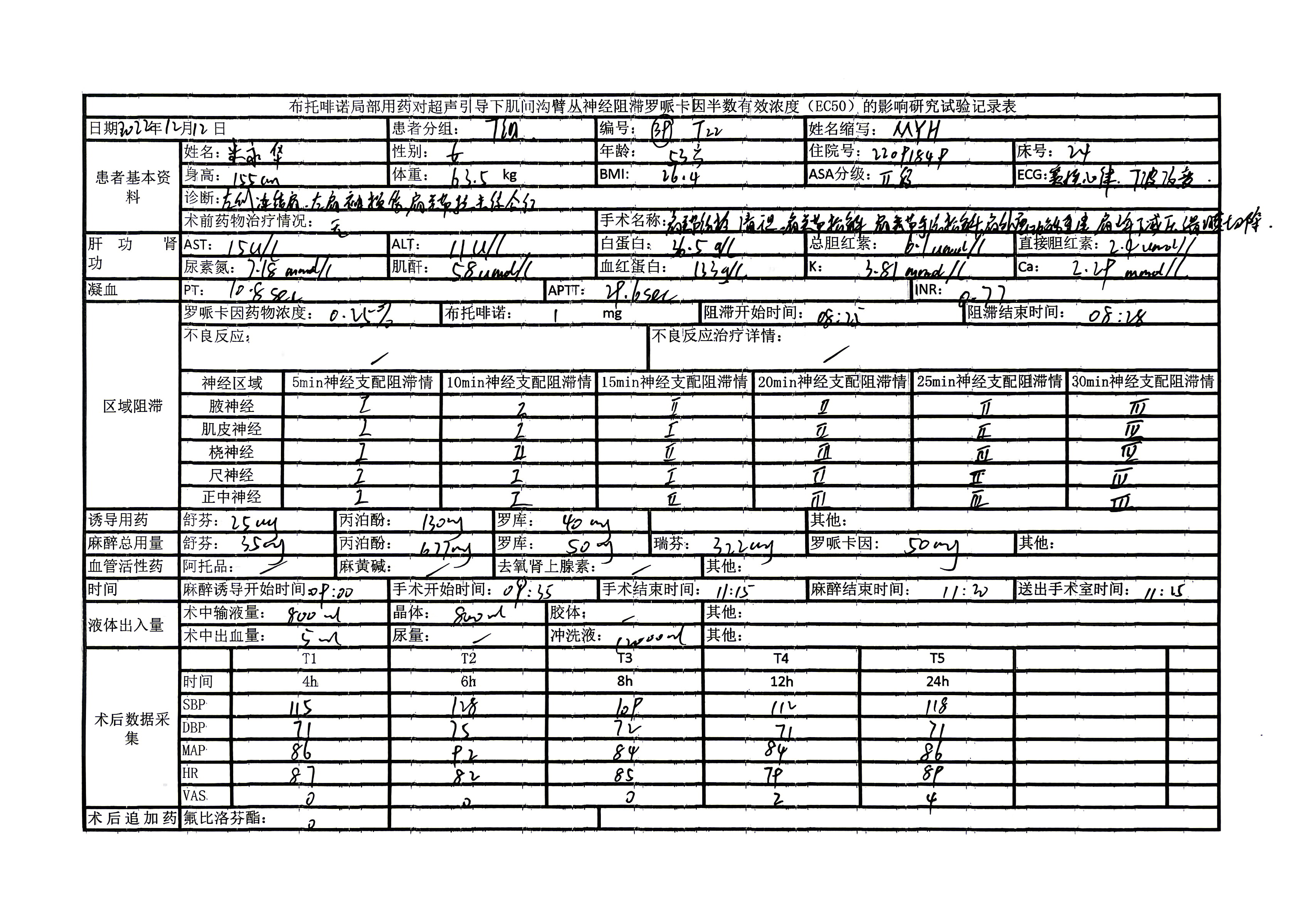

Supplement: S4 File — (ZIP) [file pone.0350613.s008.zip › 002.jpg]

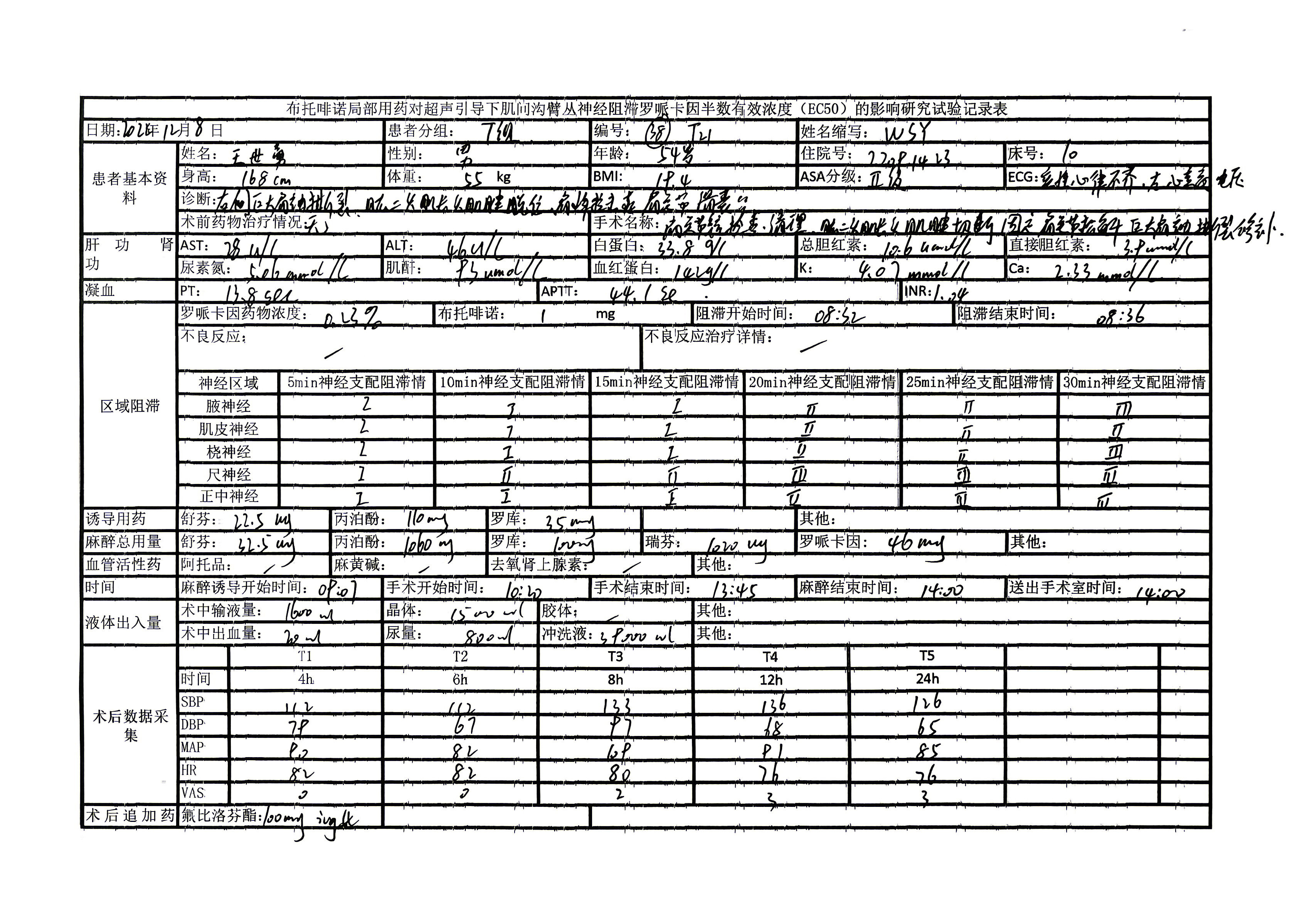

Supplement: S4 File — (ZIP) [file pone.0350613.s008.zip › 003.jpg]

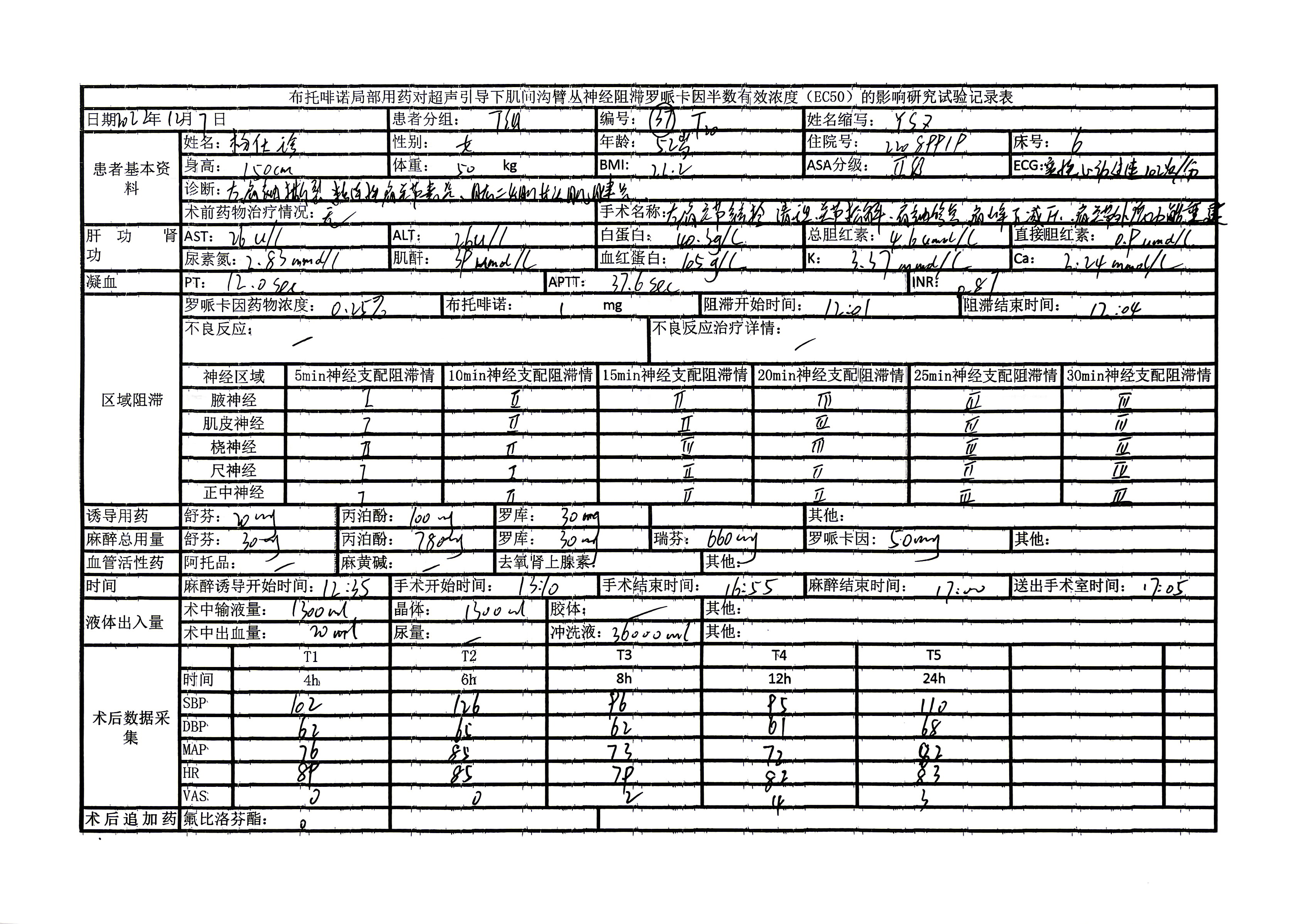

Supplement: S4 File — (ZIP) [file pone.0350613.s008.zip › 004.jpg]

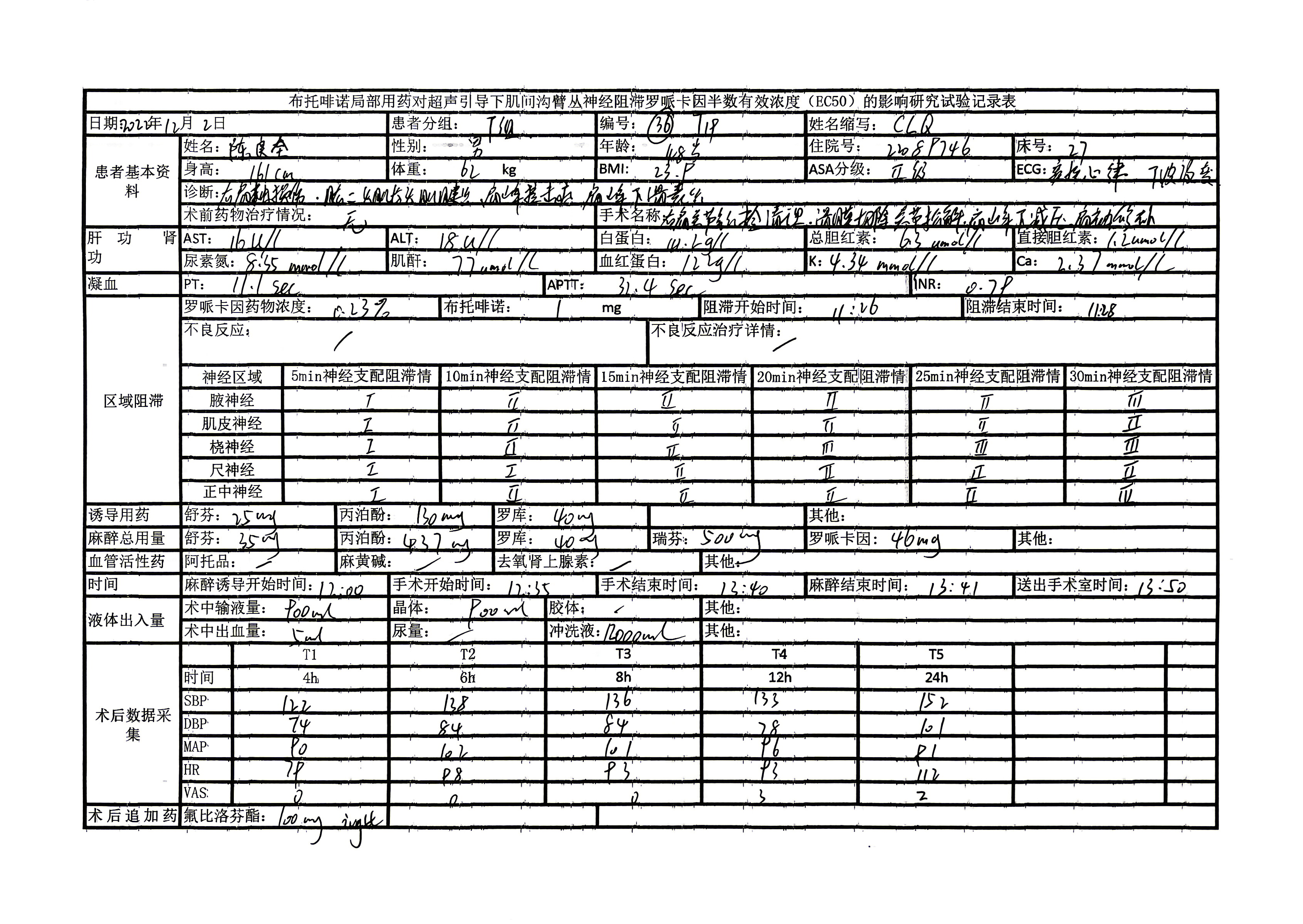

Supplement: S4 File — (ZIP) [file pone.0350613.s008.zip › 005.jpg]

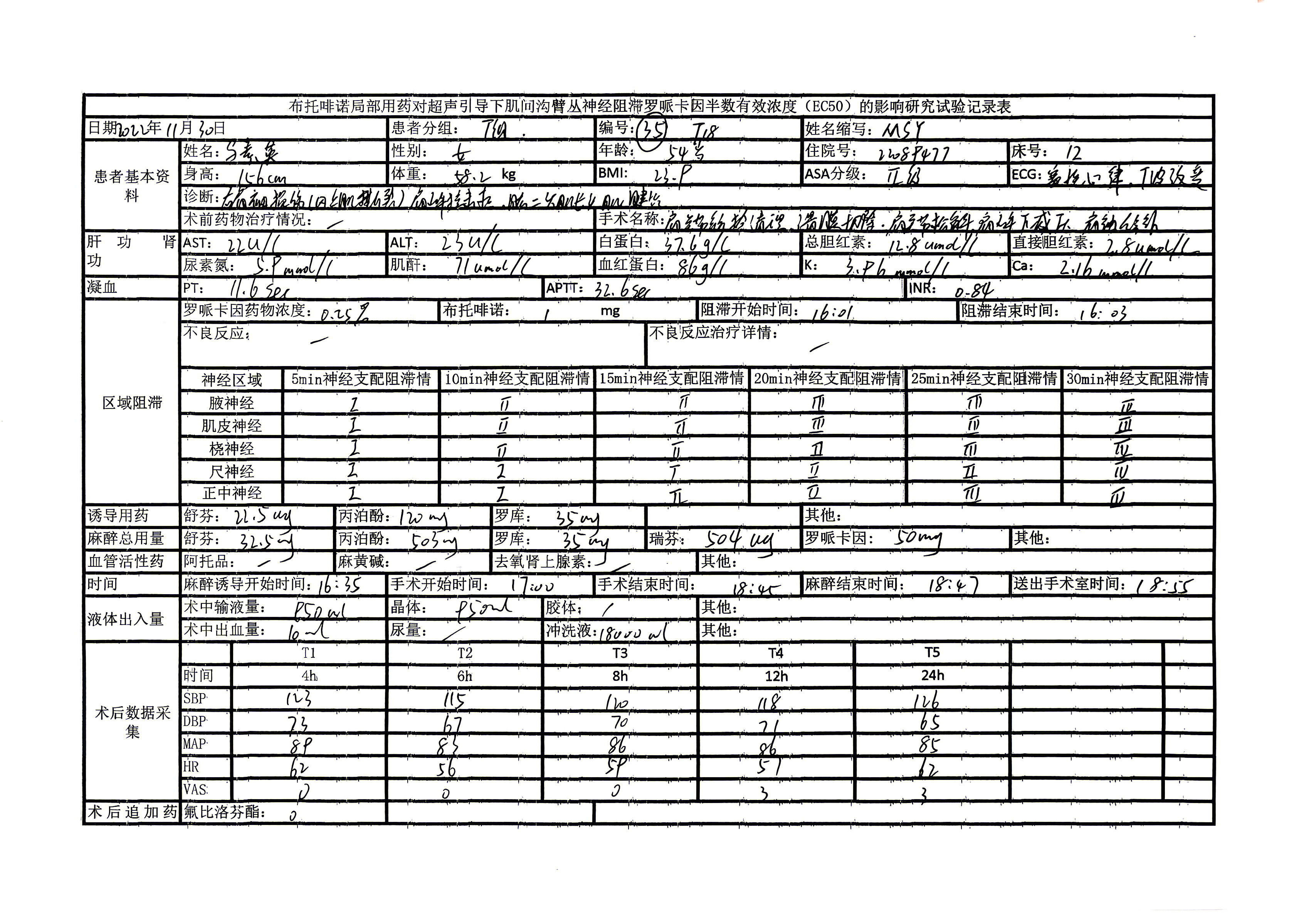

Supplement: S4 File — (ZIP) [file pone.0350613.s008.zip › 006.jpg]

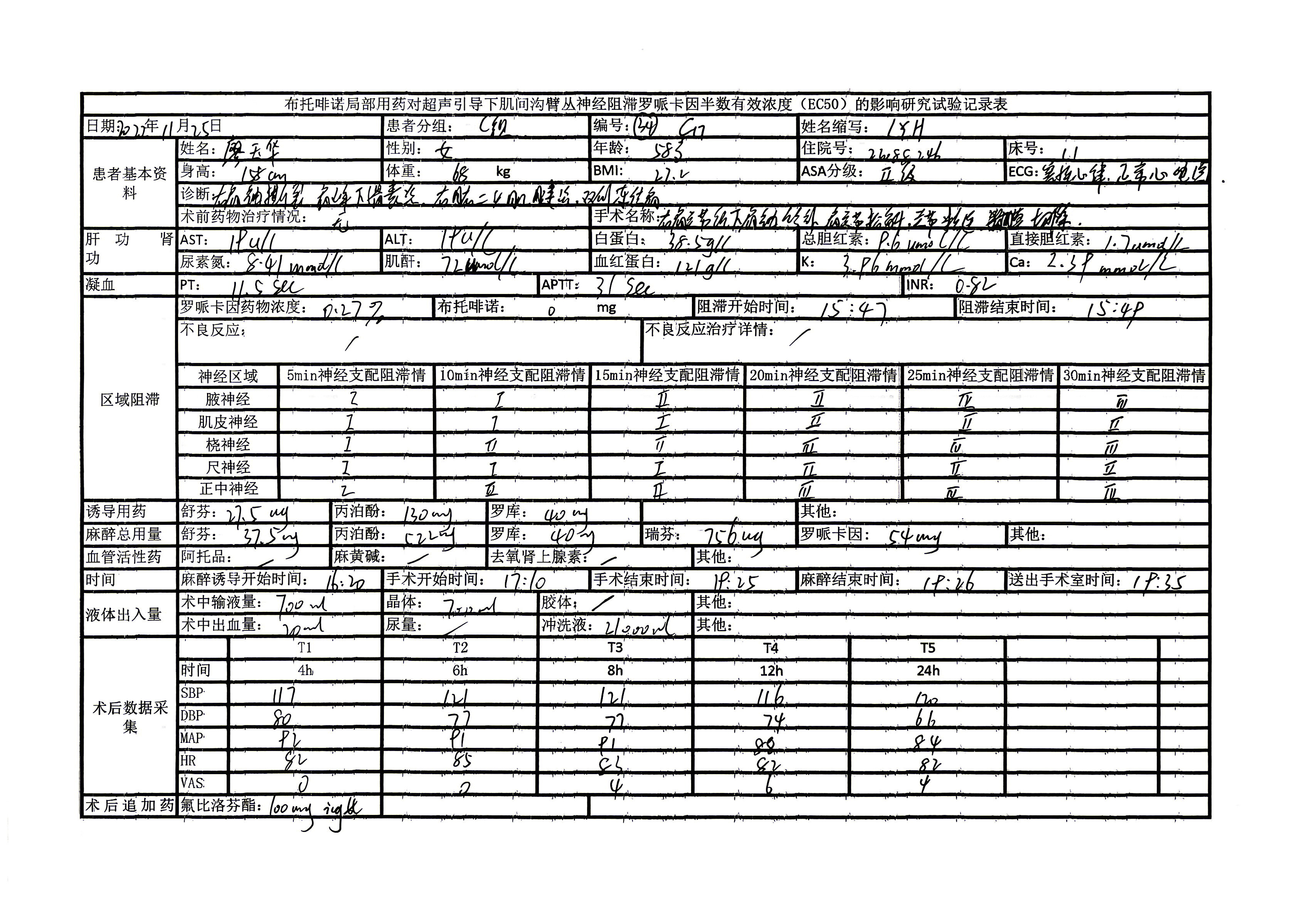

Supplement: S4 File — (ZIP) [file pone.0350613.s008.zip › 007.jpg]

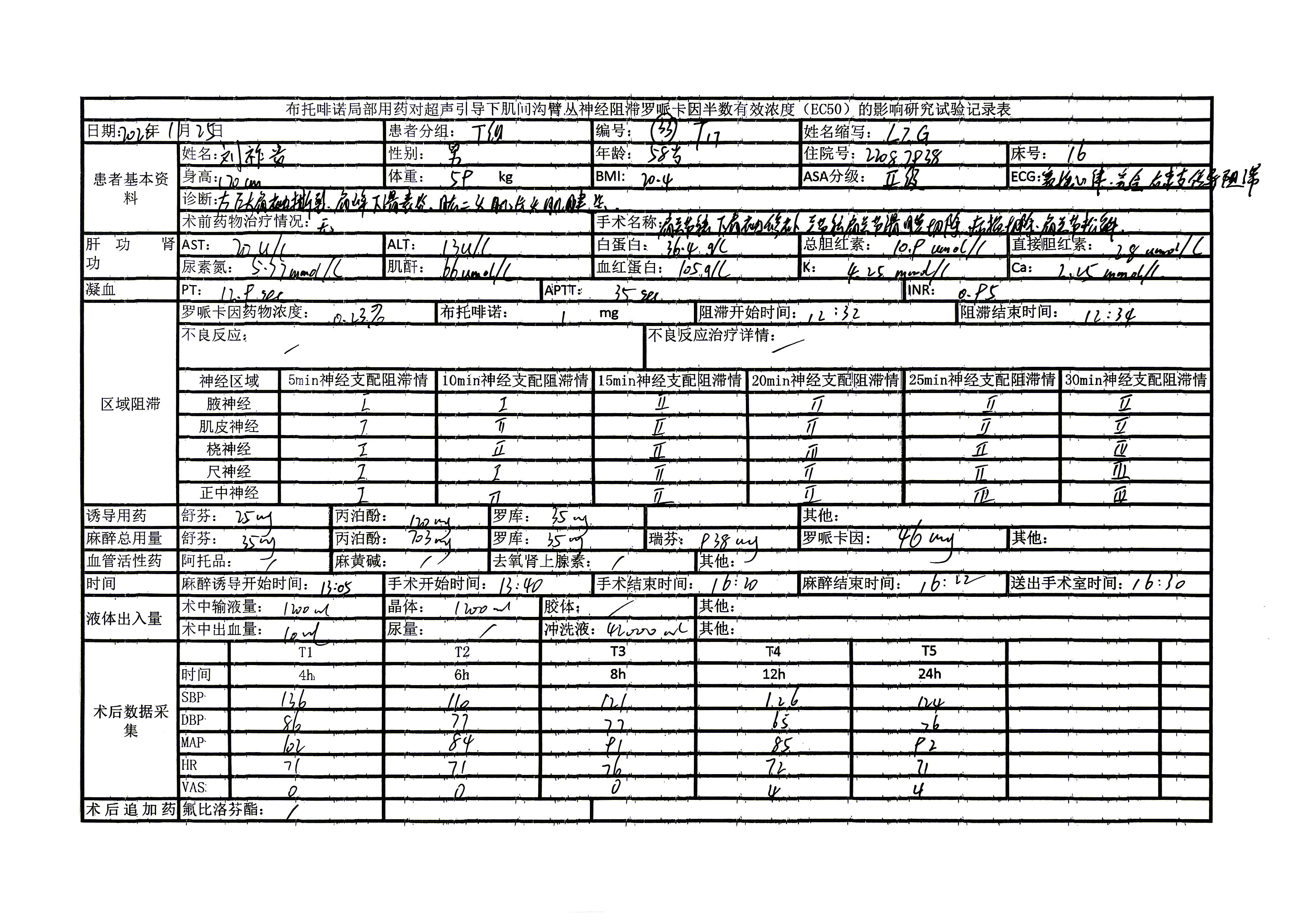

Supplement: S4 File — (ZIP) [file pone.0350613.s008.zip › 008.jpg]

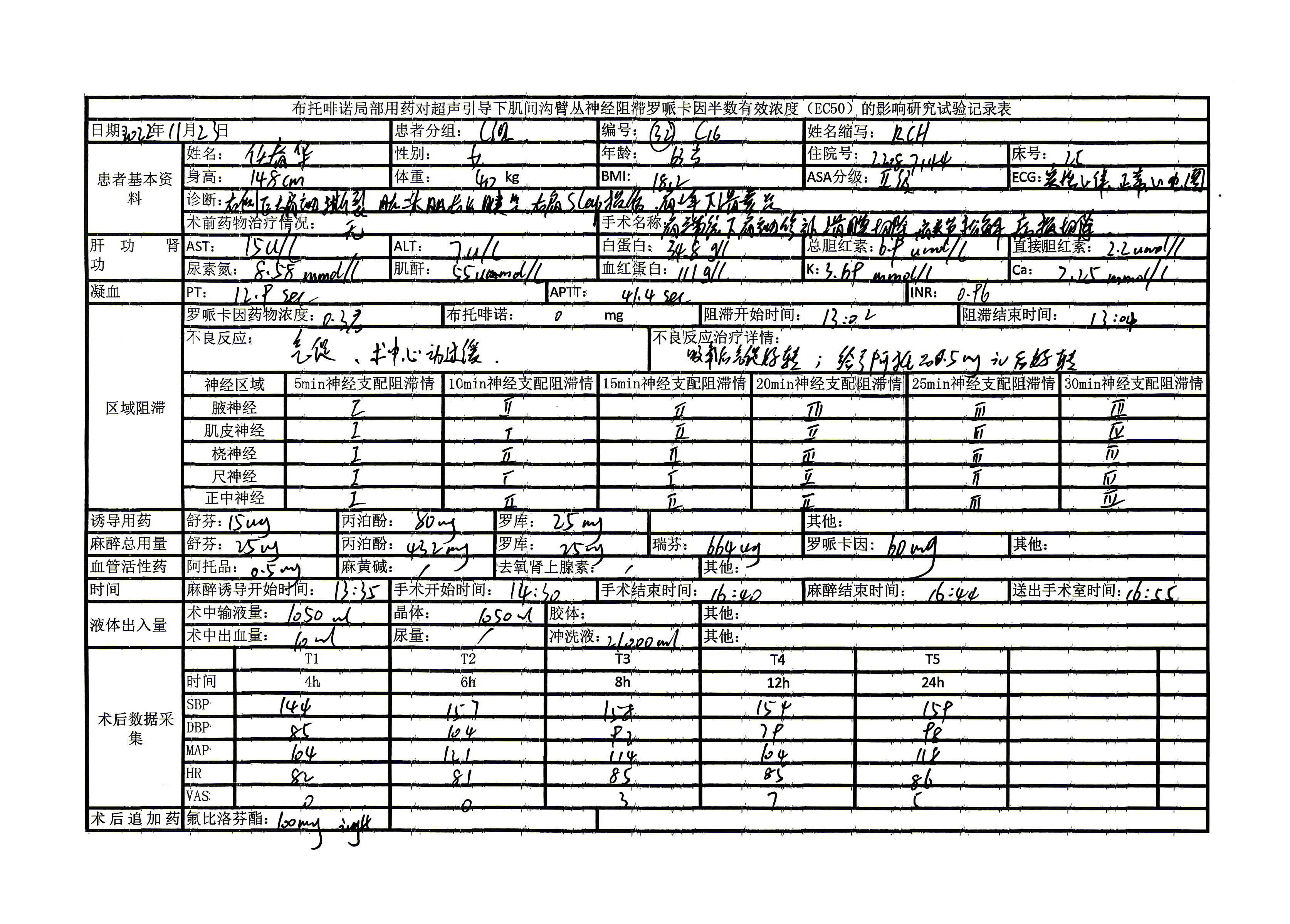

Supplement: S4 File — (ZIP) [file pone.0350613.s008.zip › 009.jpg]

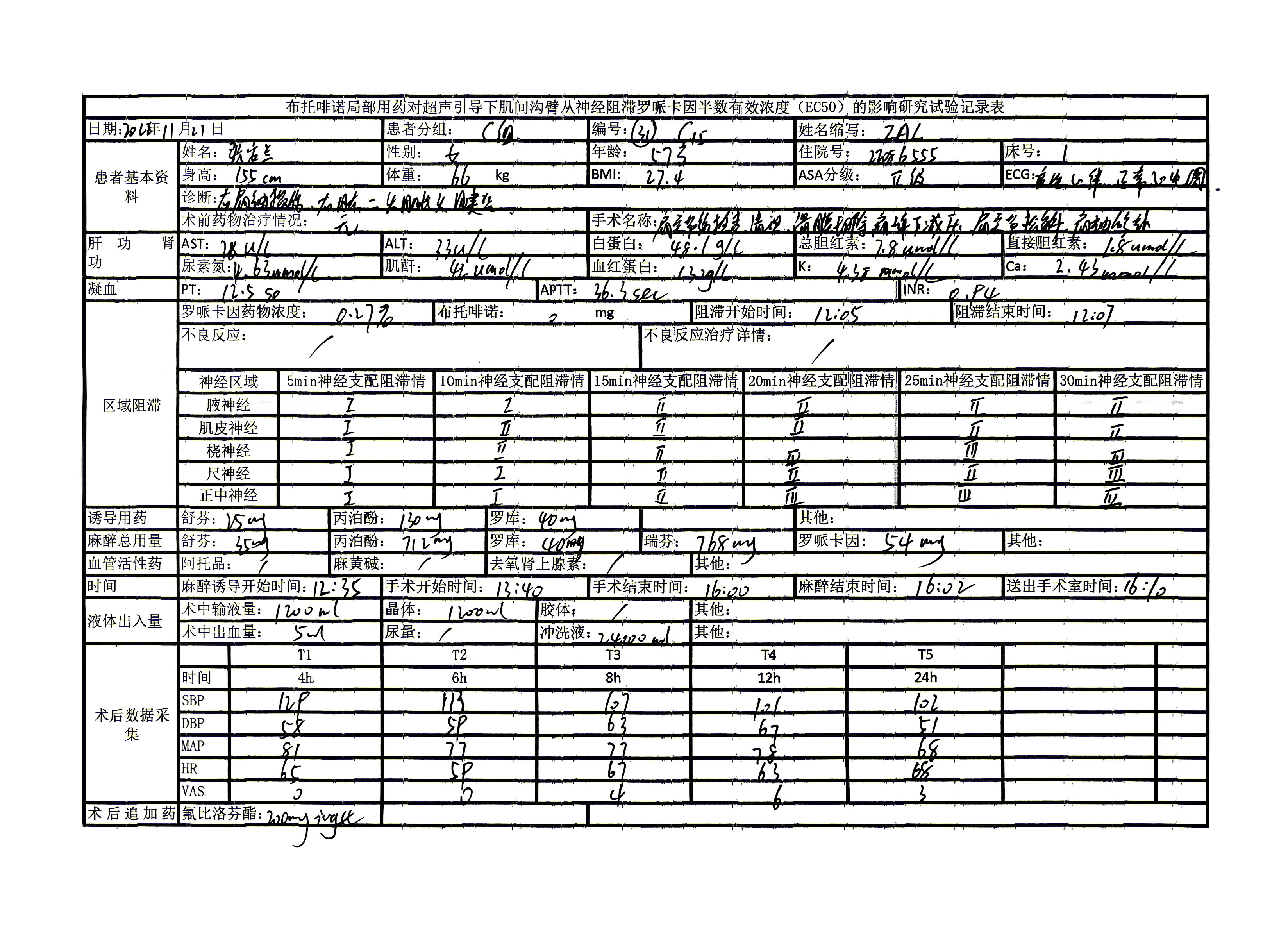

Supplement: S4 File — (ZIP) [file pone.0350613.s008.zip › 010.jpg]

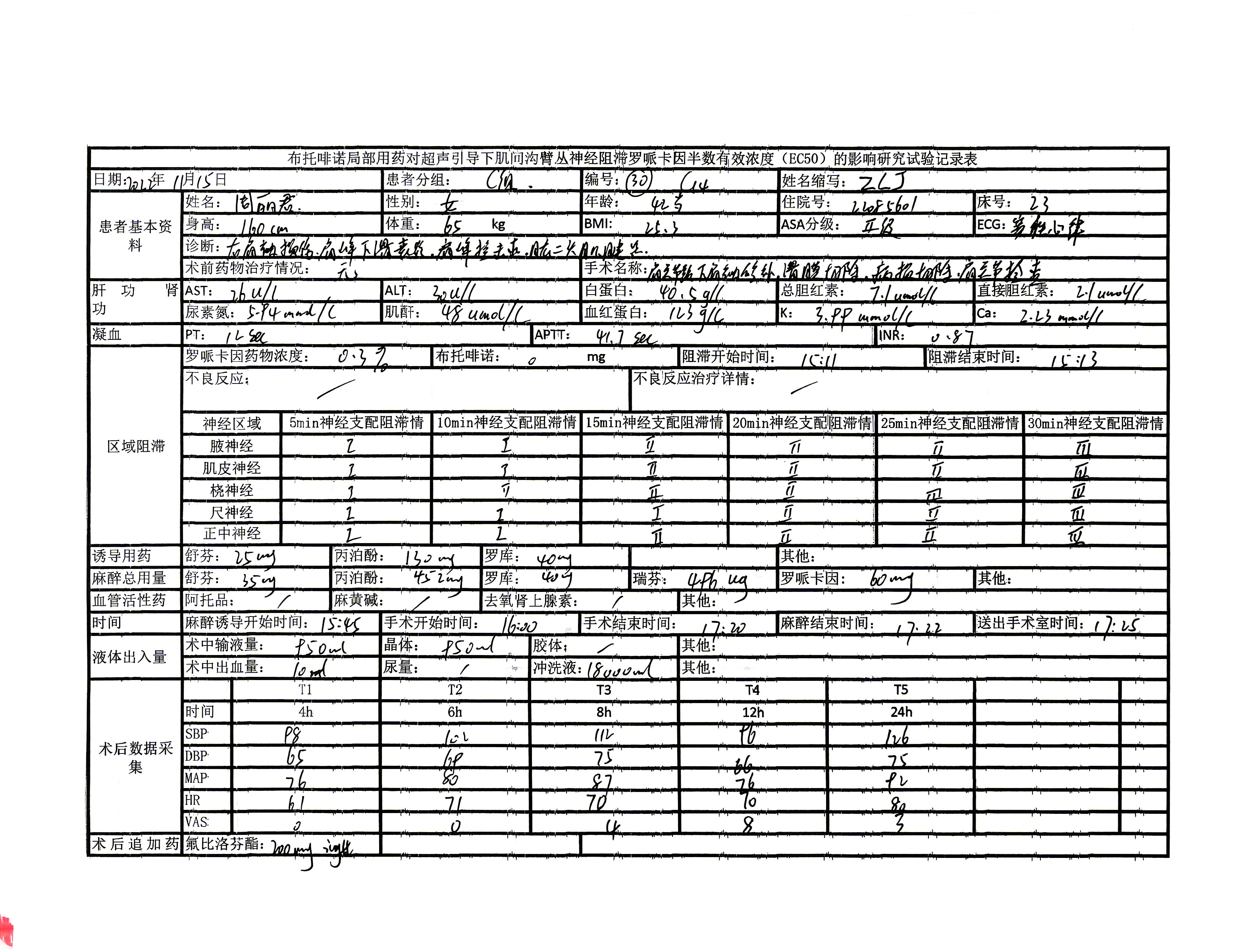

Supplement: S5 File — (ZIP) [file pone.0350613.s009.zip › 011.jpg]

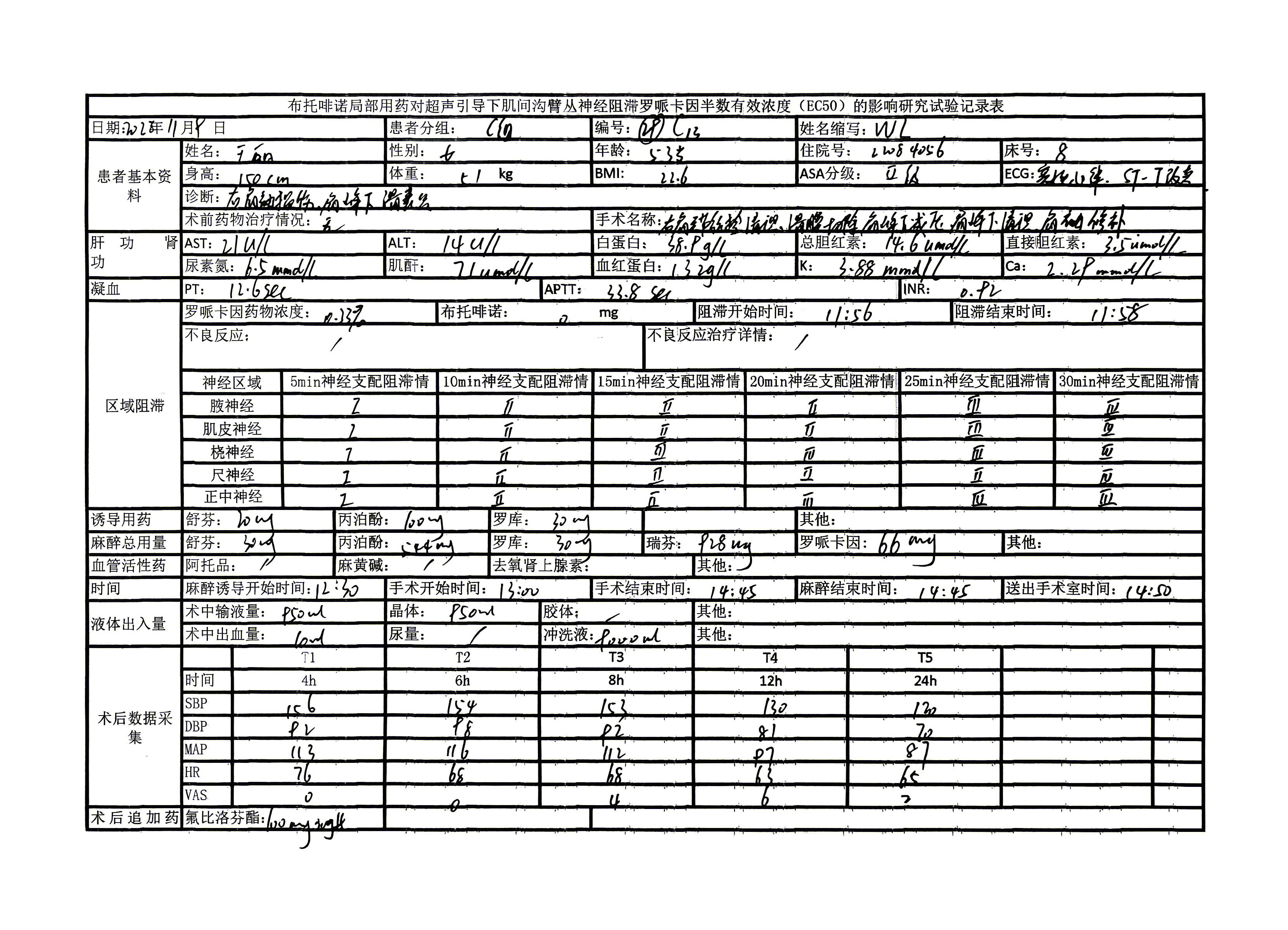

Supplement: S5 File — (ZIP) [file pone.0350613.s009.zip › 012.jpg]

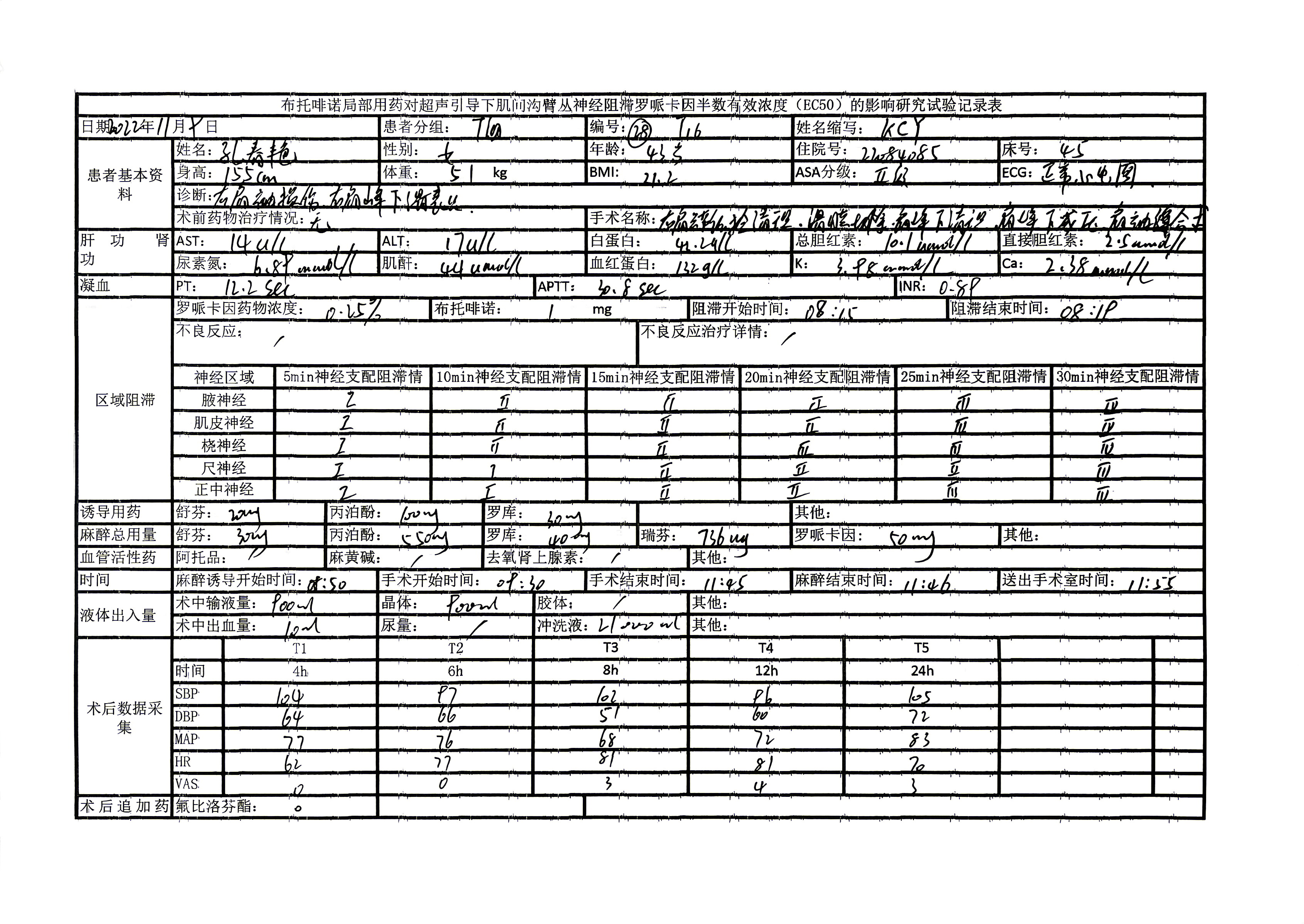

Supplement: S5 File — (ZIP) [file pone.0350613.s009.zip › 013.jpg]

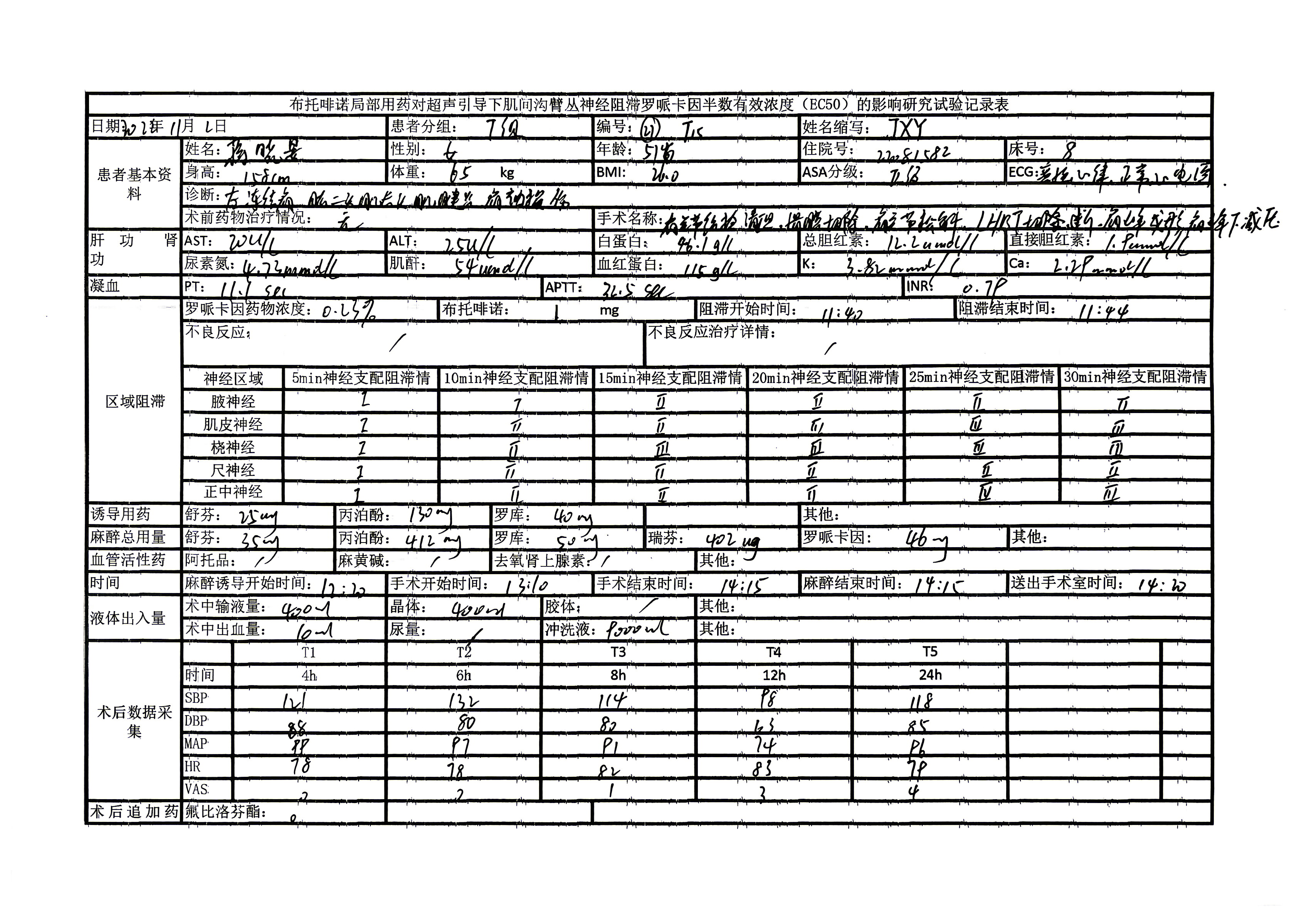

Supplement: S5 File — (ZIP) [file pone.0350613.s009.zip › 014.jpg]

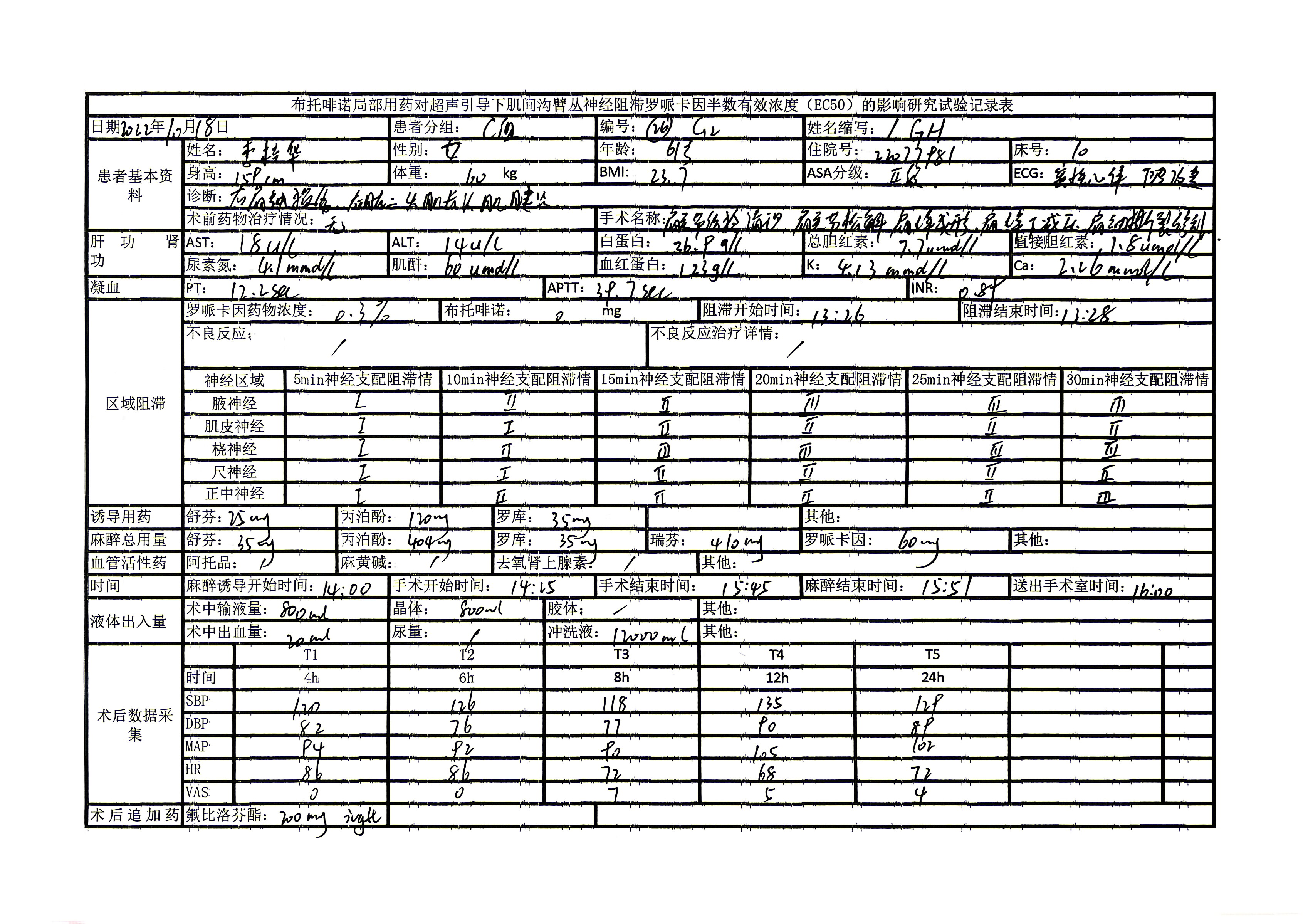

Supplement: S5 File — (ZIP) [file pone.0350613.s009.zip › 015.jpg]

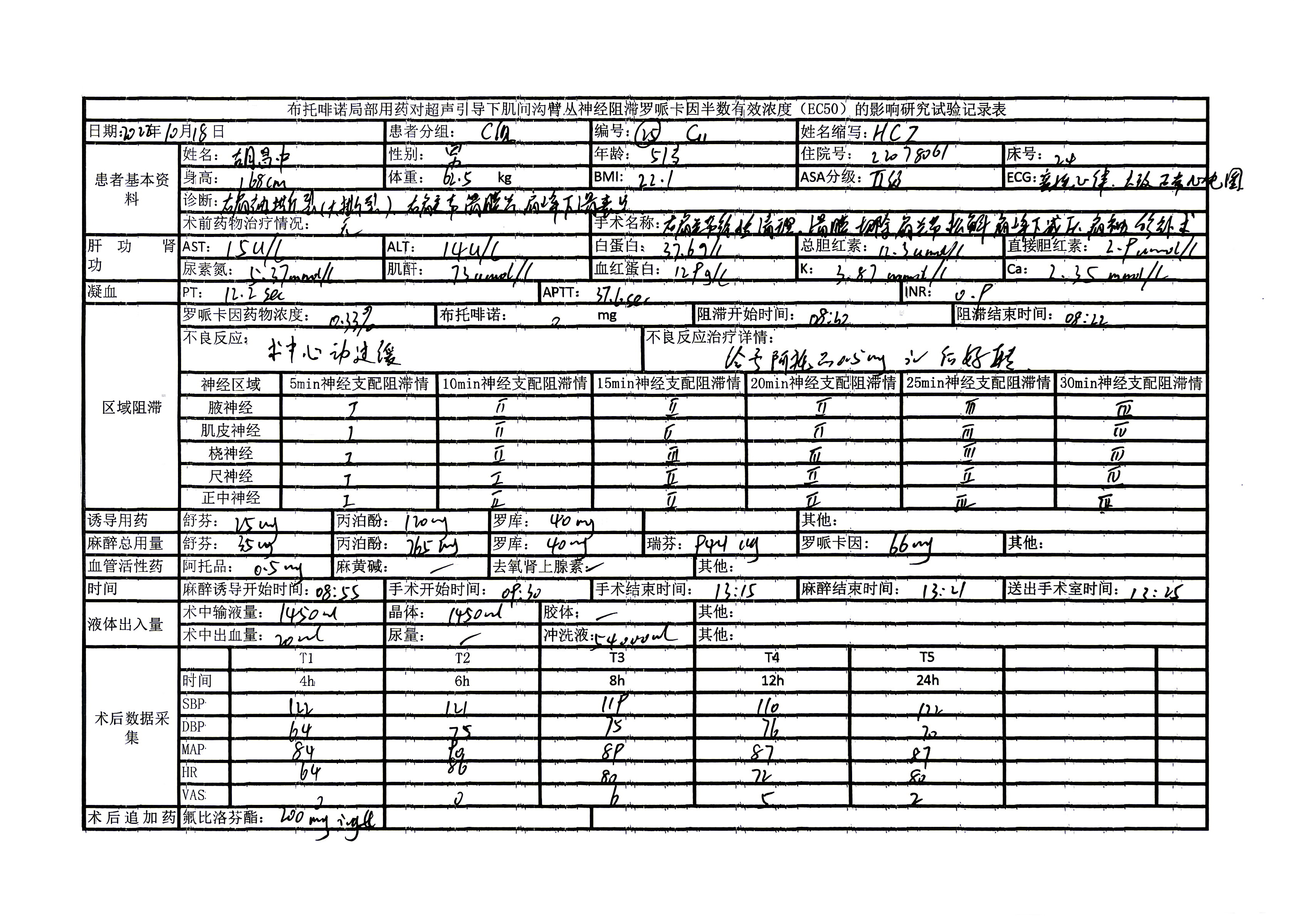

Supplement: S5 File — (ZIP) [file pone.0350613.s009.zip › 016.jpg]

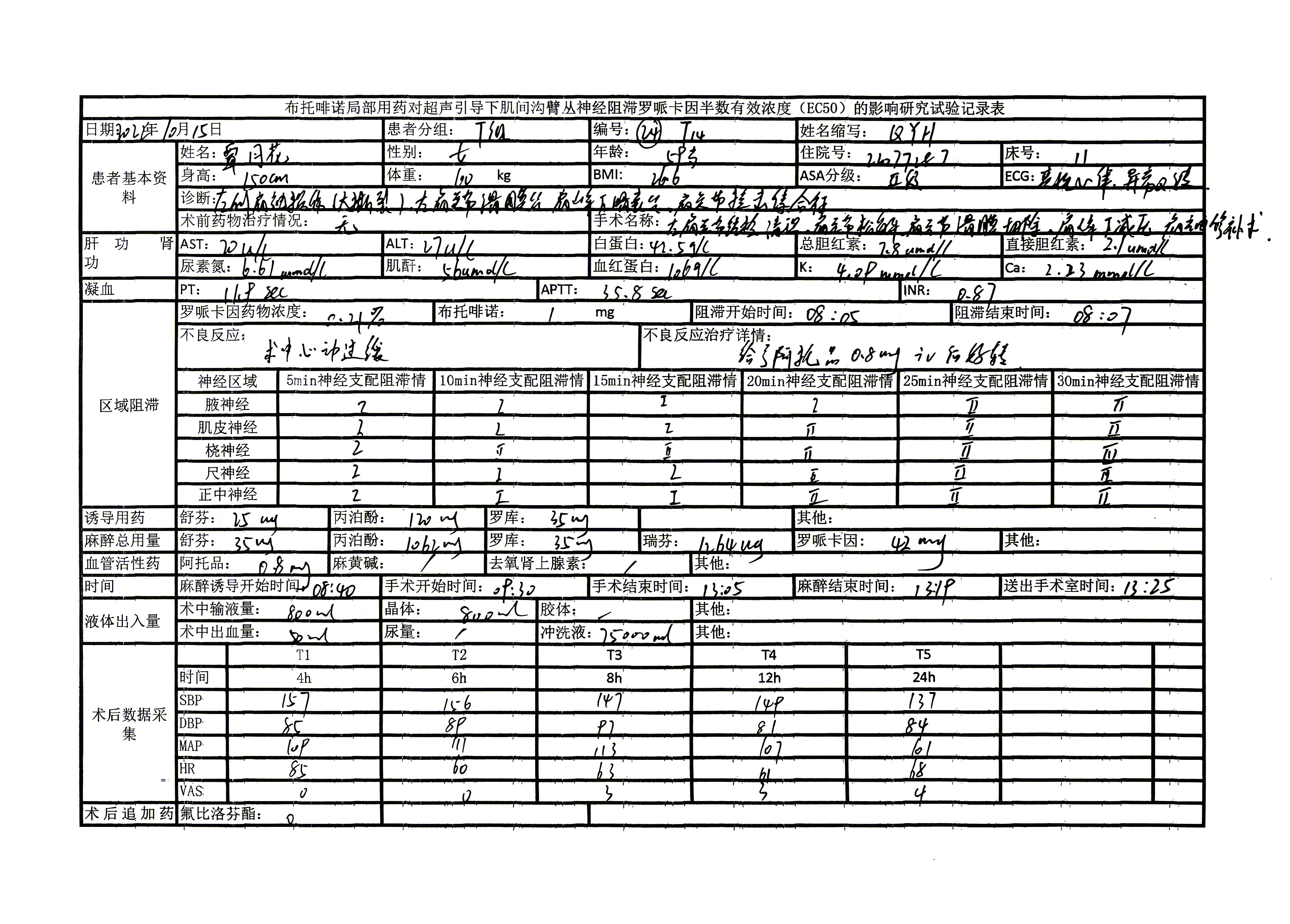

Supplement: S5 File — (ZIP) [file pone.0350613.s009.zip › 017.jpg]

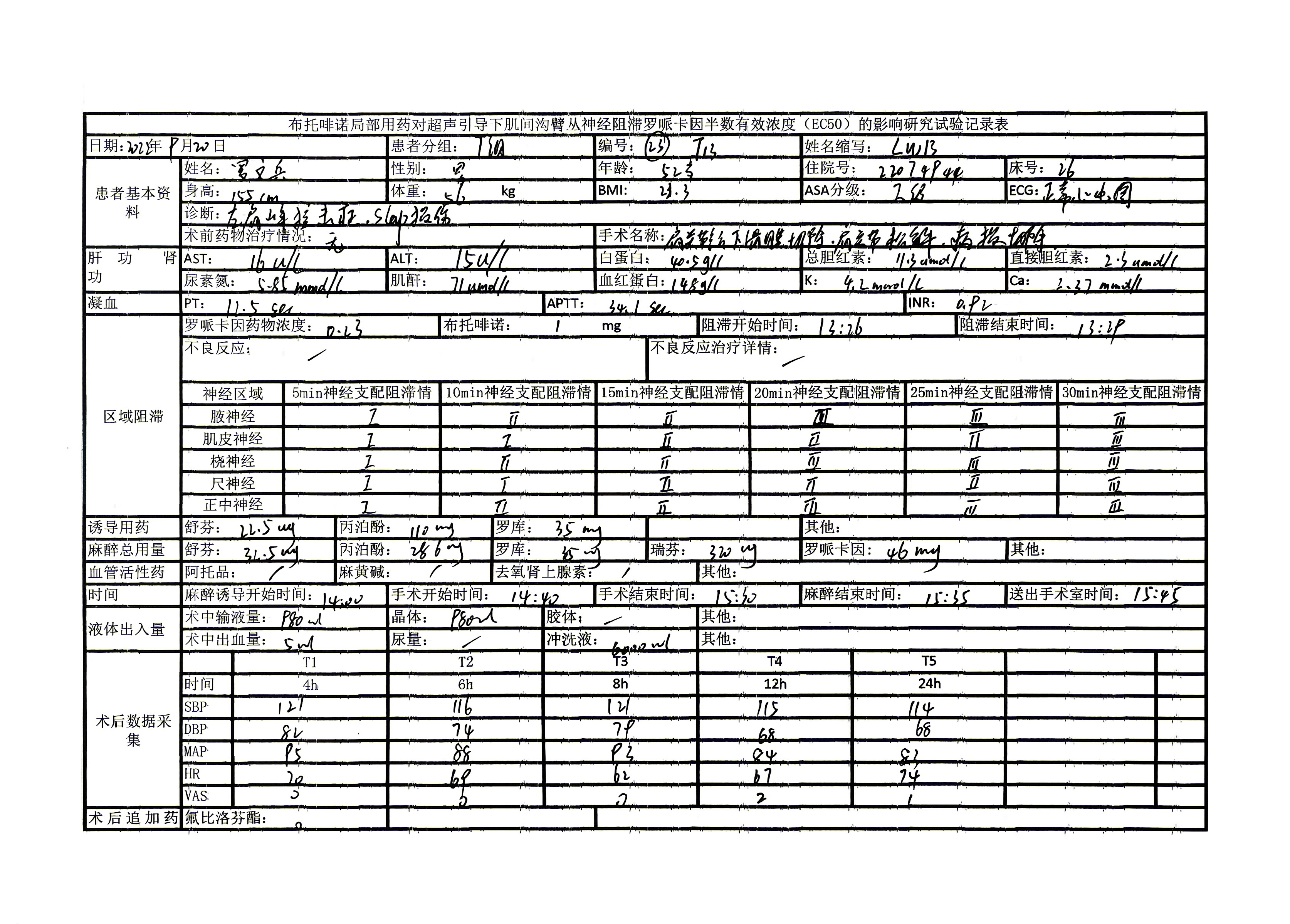

Supplement: S5 File — (ZIP) [file pone.0350613.s009.zip › 018.jpg]

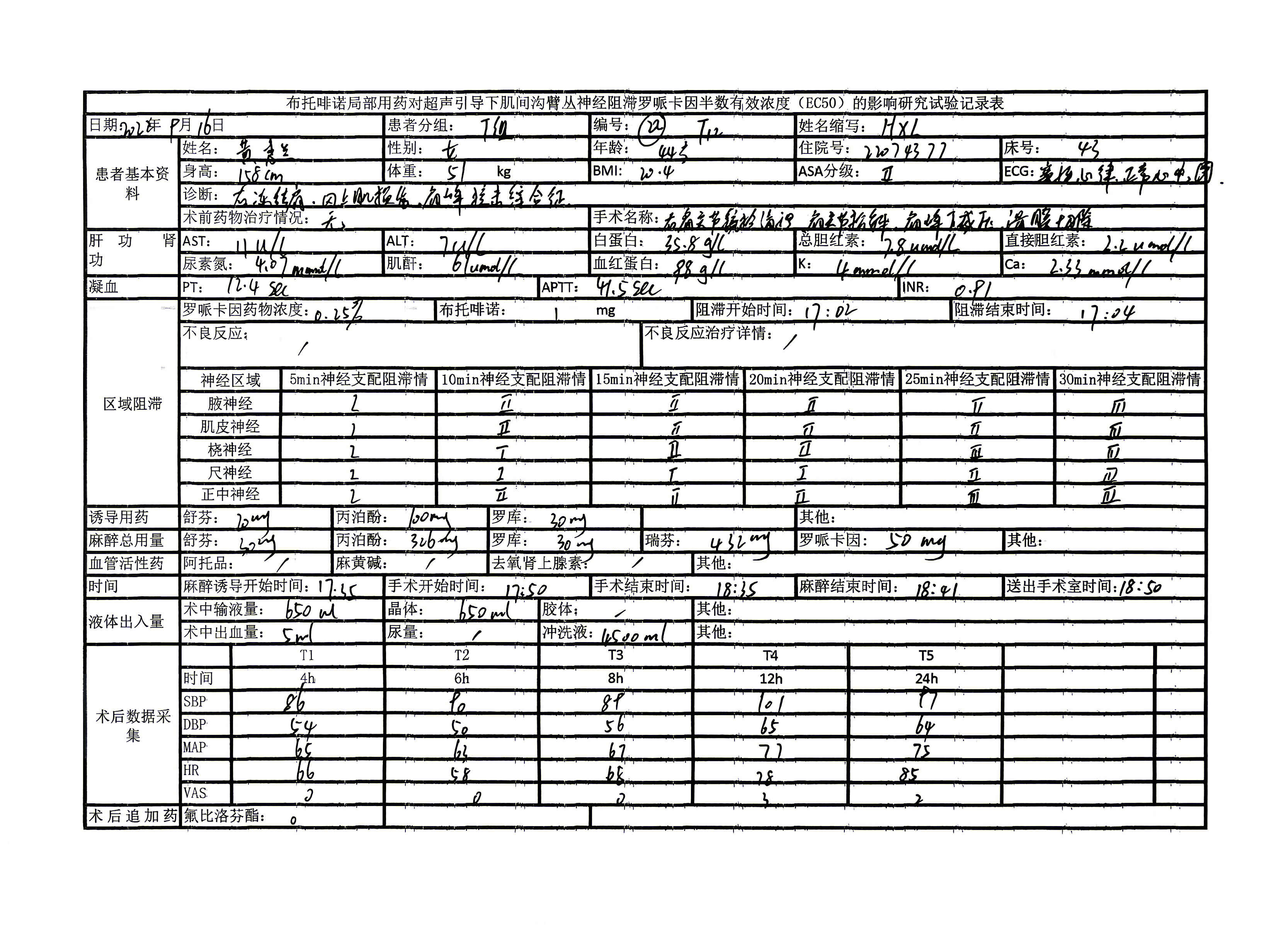

Supplement: S5 File — (ZIP) [file pone.0350613.s009.zip › 019.jpg]

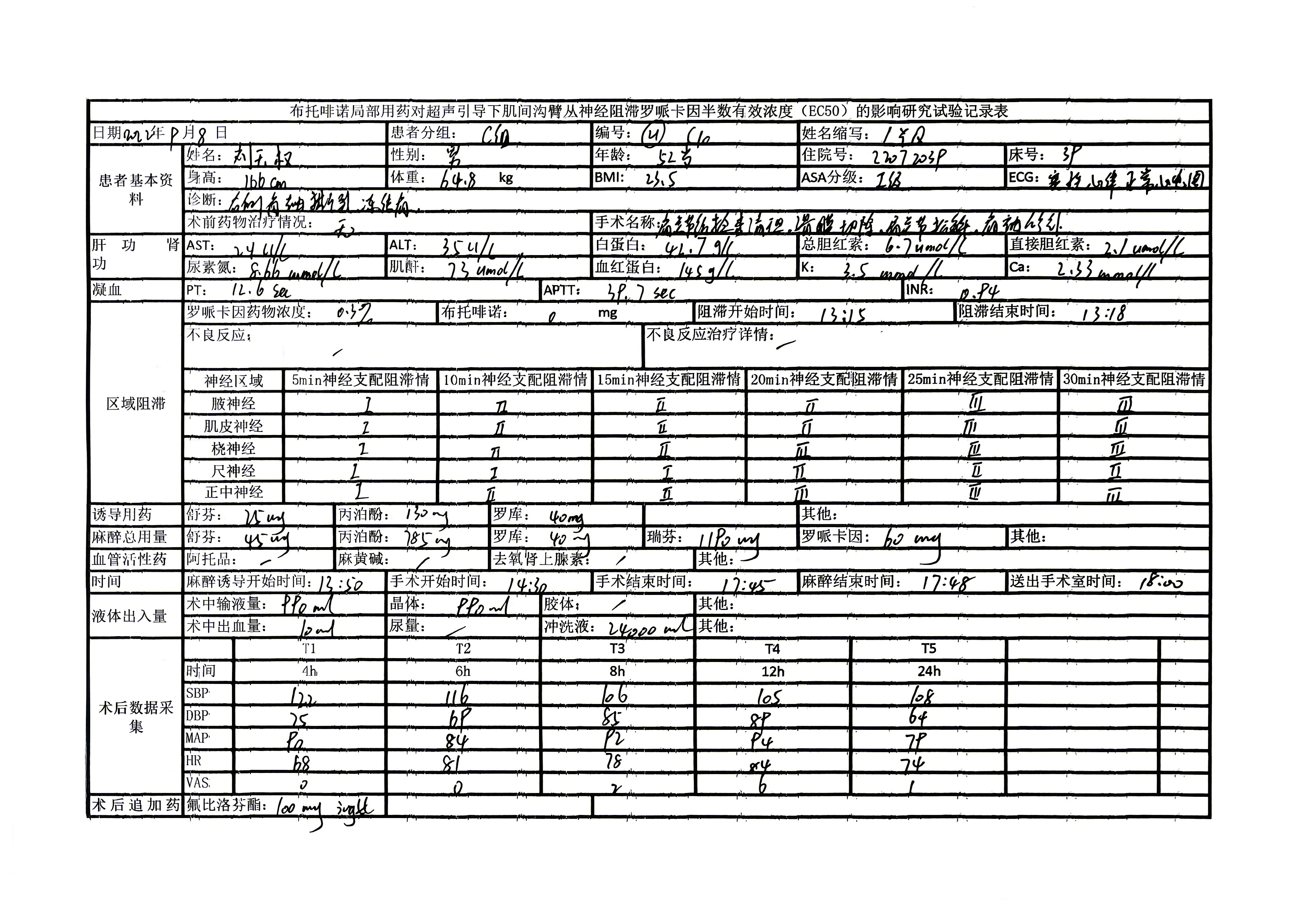

Supplement: S5 File — (ZIP) [file pone.0350613.s009.zip › 020.jpg]

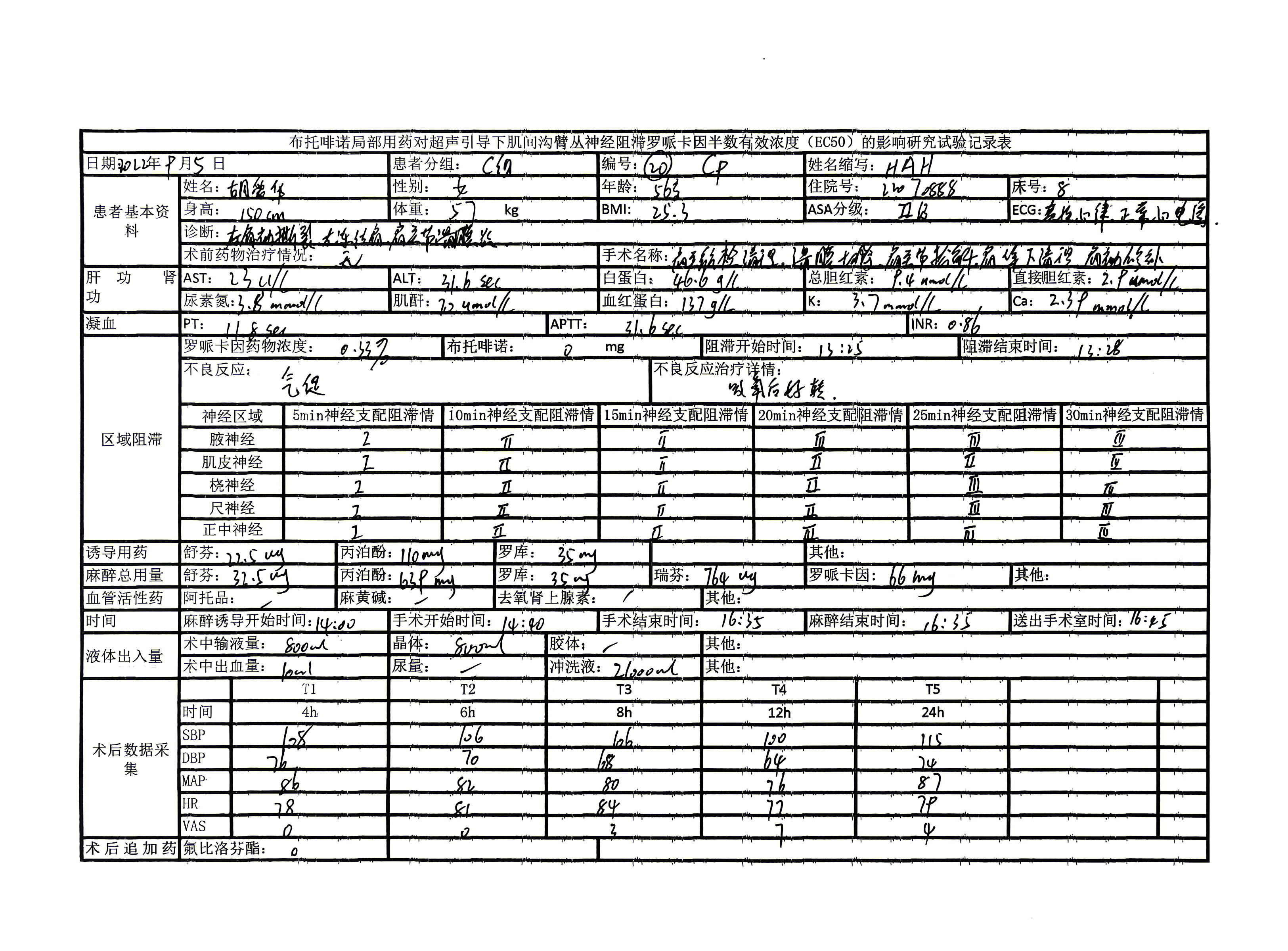

Supplement: S6 File — (ZIP) [file pone.0350613.s010.zip › 021.jpg]

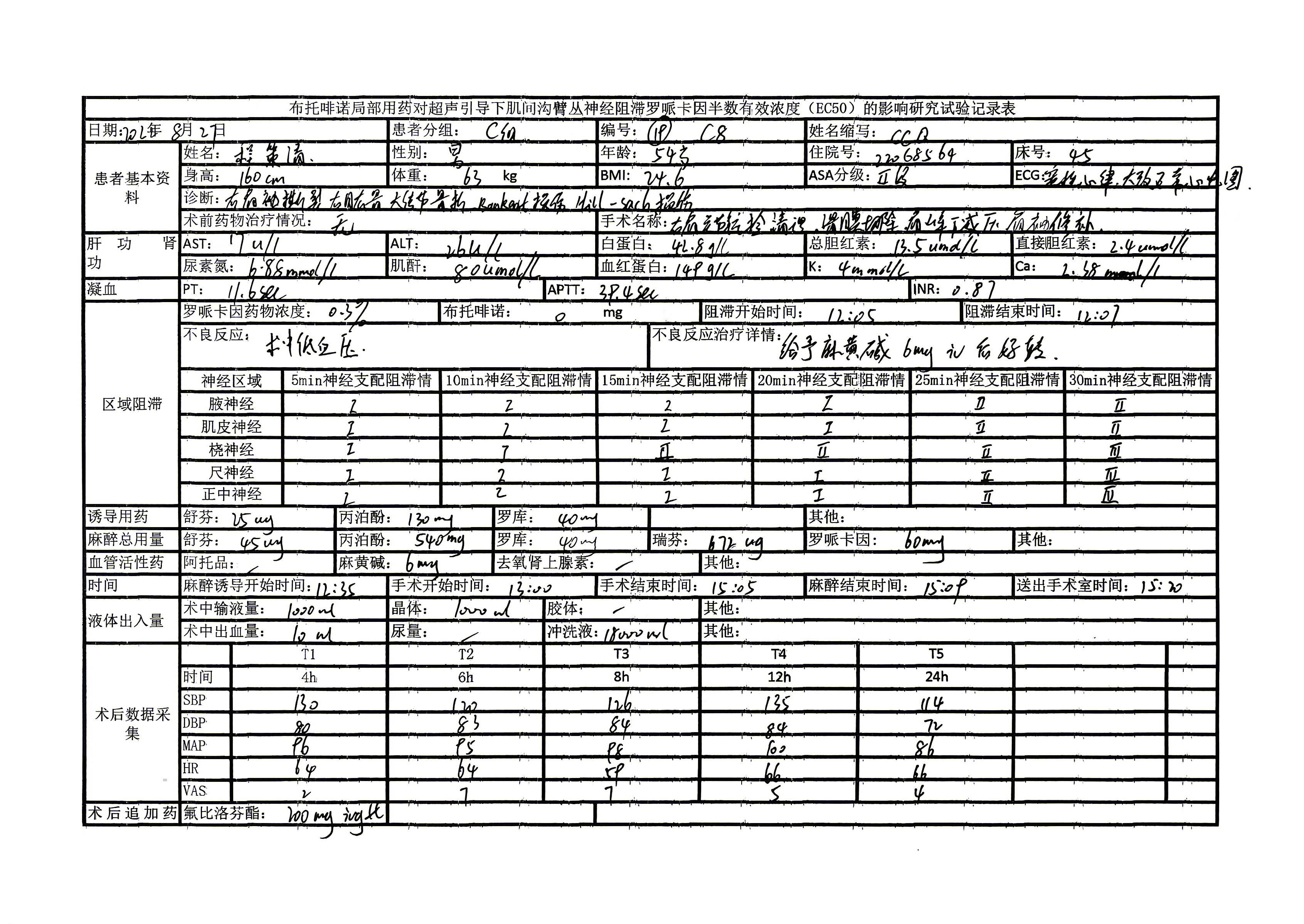

Supplement: S6 File — (ZIP) [file pone.0350613.s010.zip › 022.jpg]

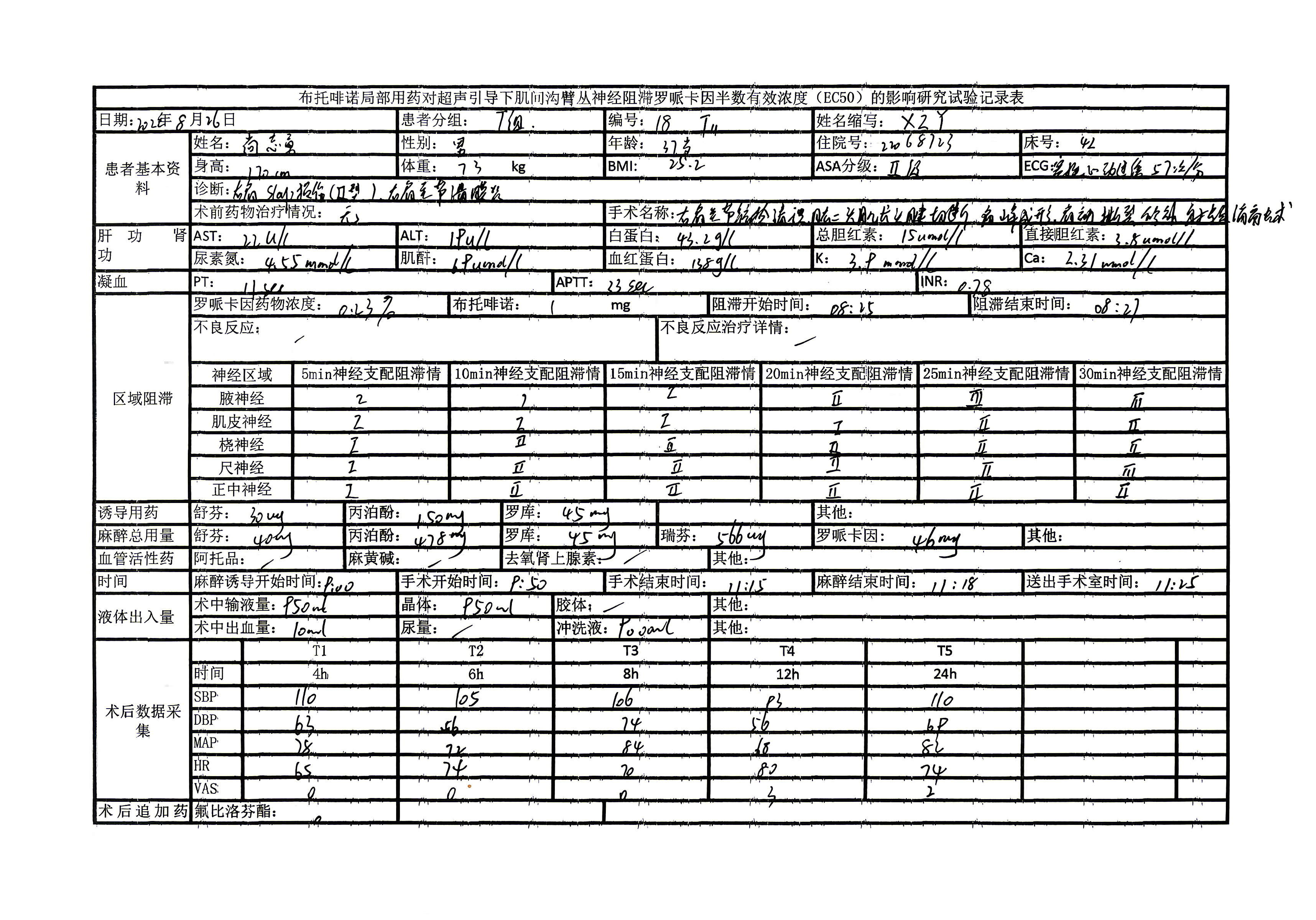

Supplement: S6 File — (ZIP) [file pone.0350613.s010.zip › 023.jpg]

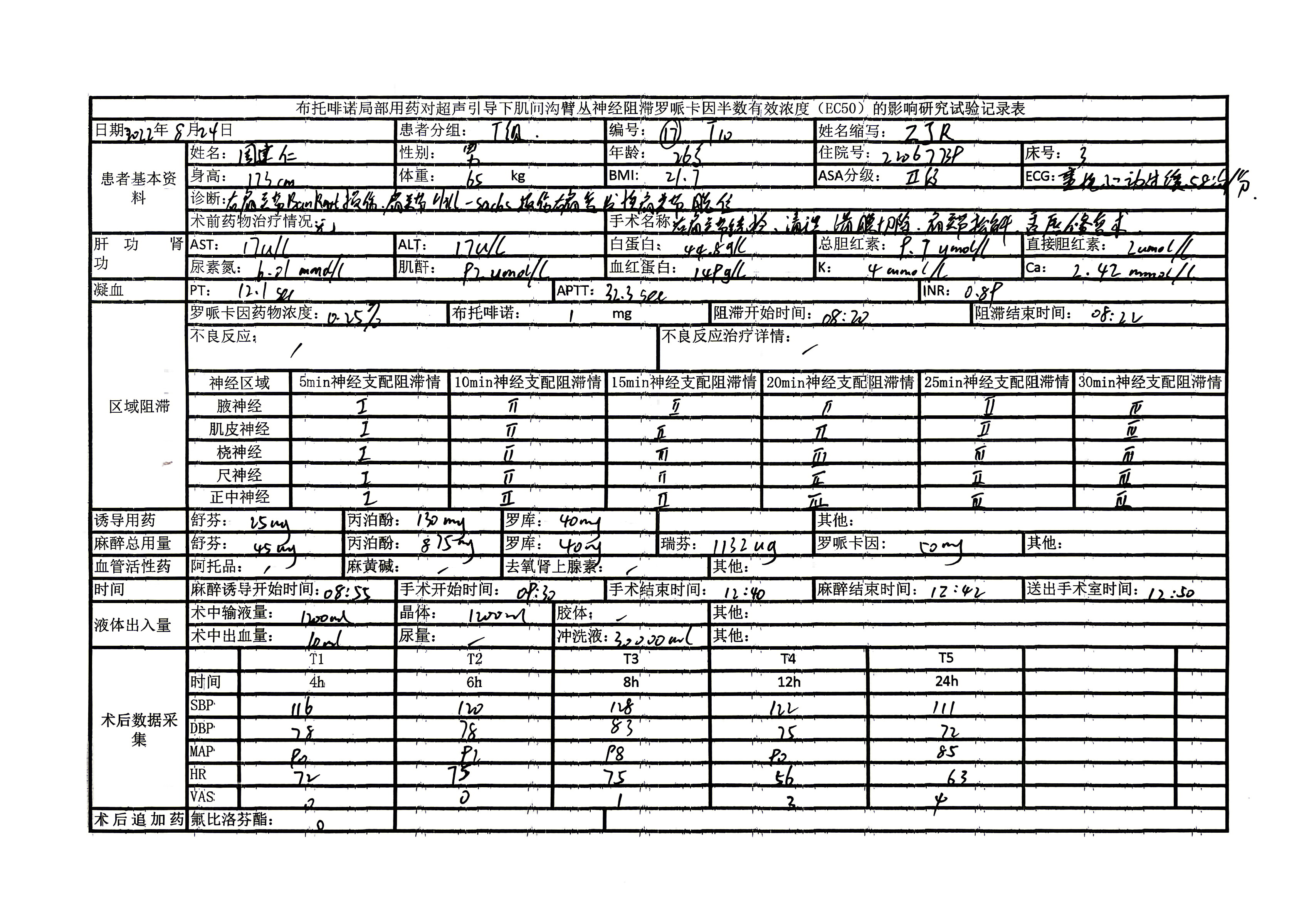

Supplement: S6 File — (ZIP) [file pone.0350613.s010.zip › 024.jpg]

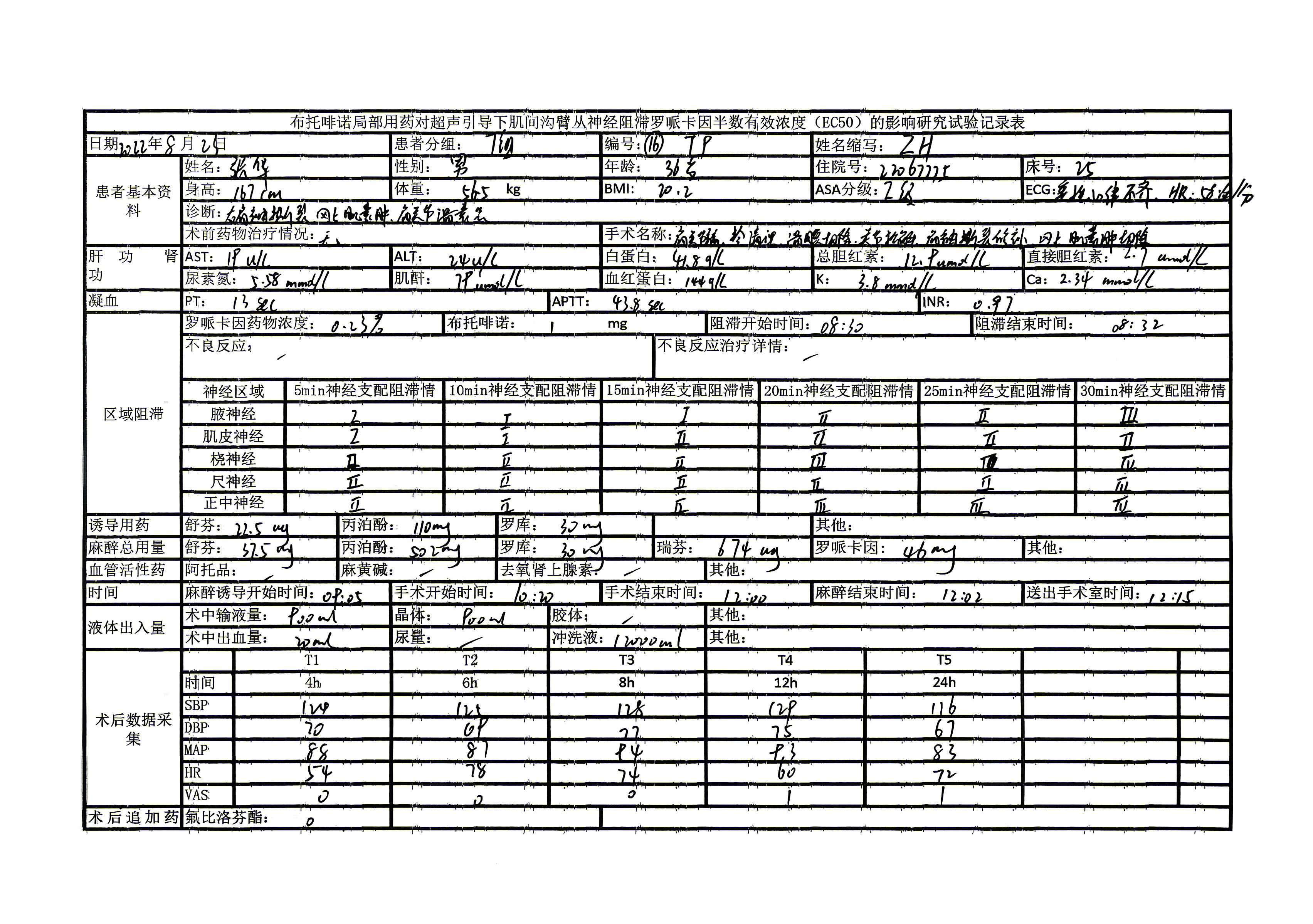

Supplement: S6 File — (ZIP) [file pone.0350613.s010.zip › 025.jpg]

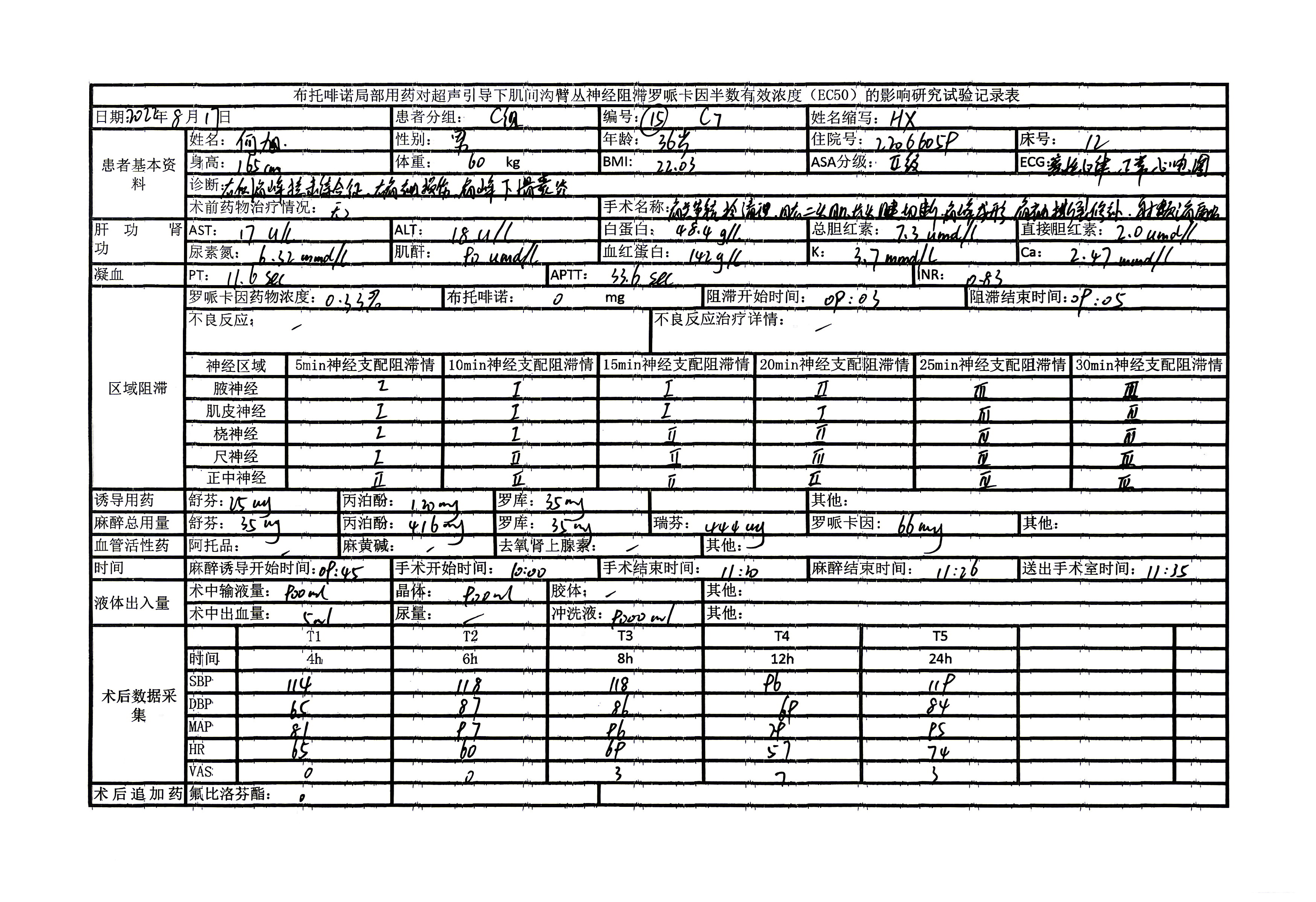

Supplement: S6 File — (ZIP) [file pone.0350613.s010.zip › 026.jpg]

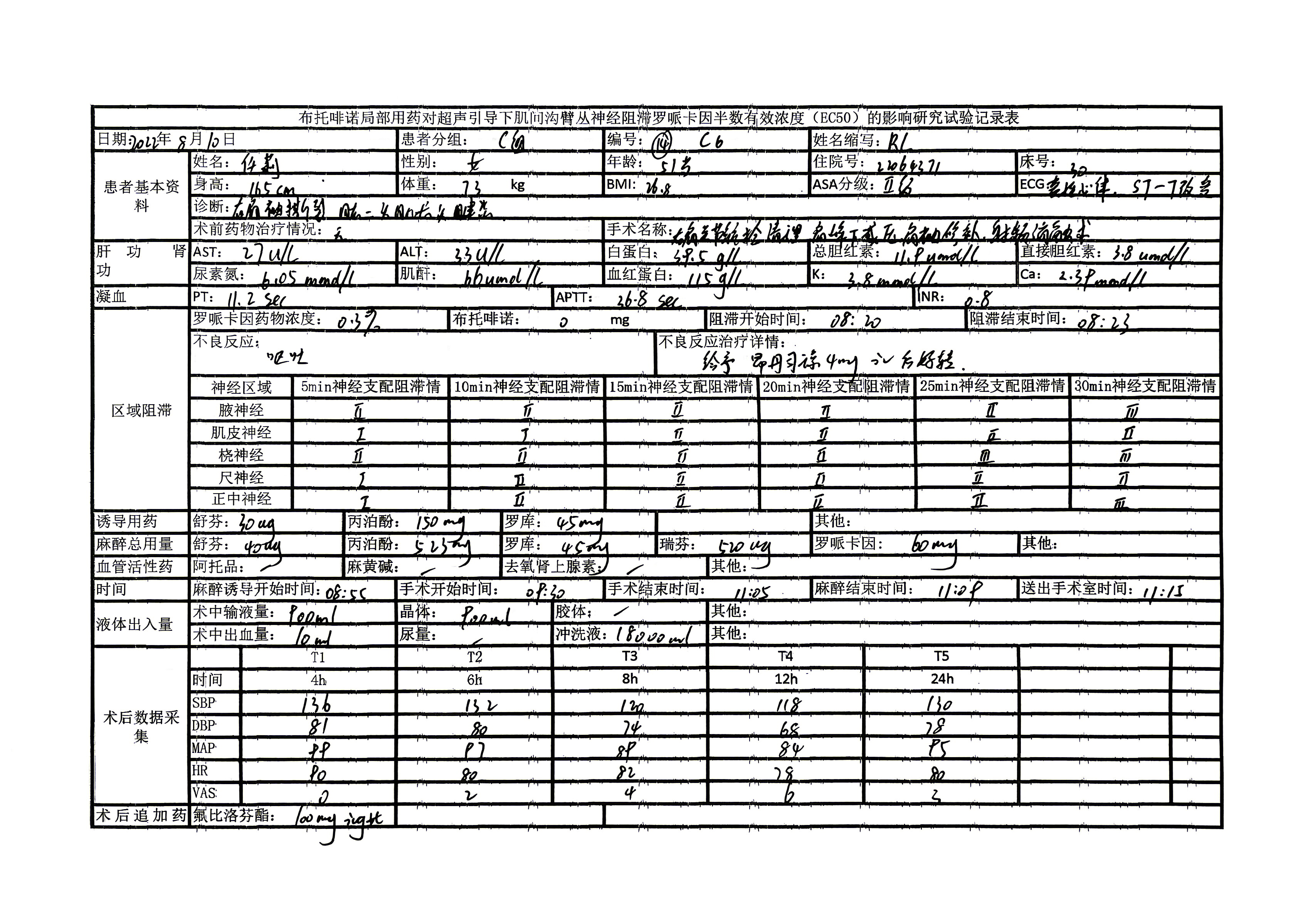

Supplement: S6 File — (ZIP) [file pone.0350613.s010.zip › 027.jpg]

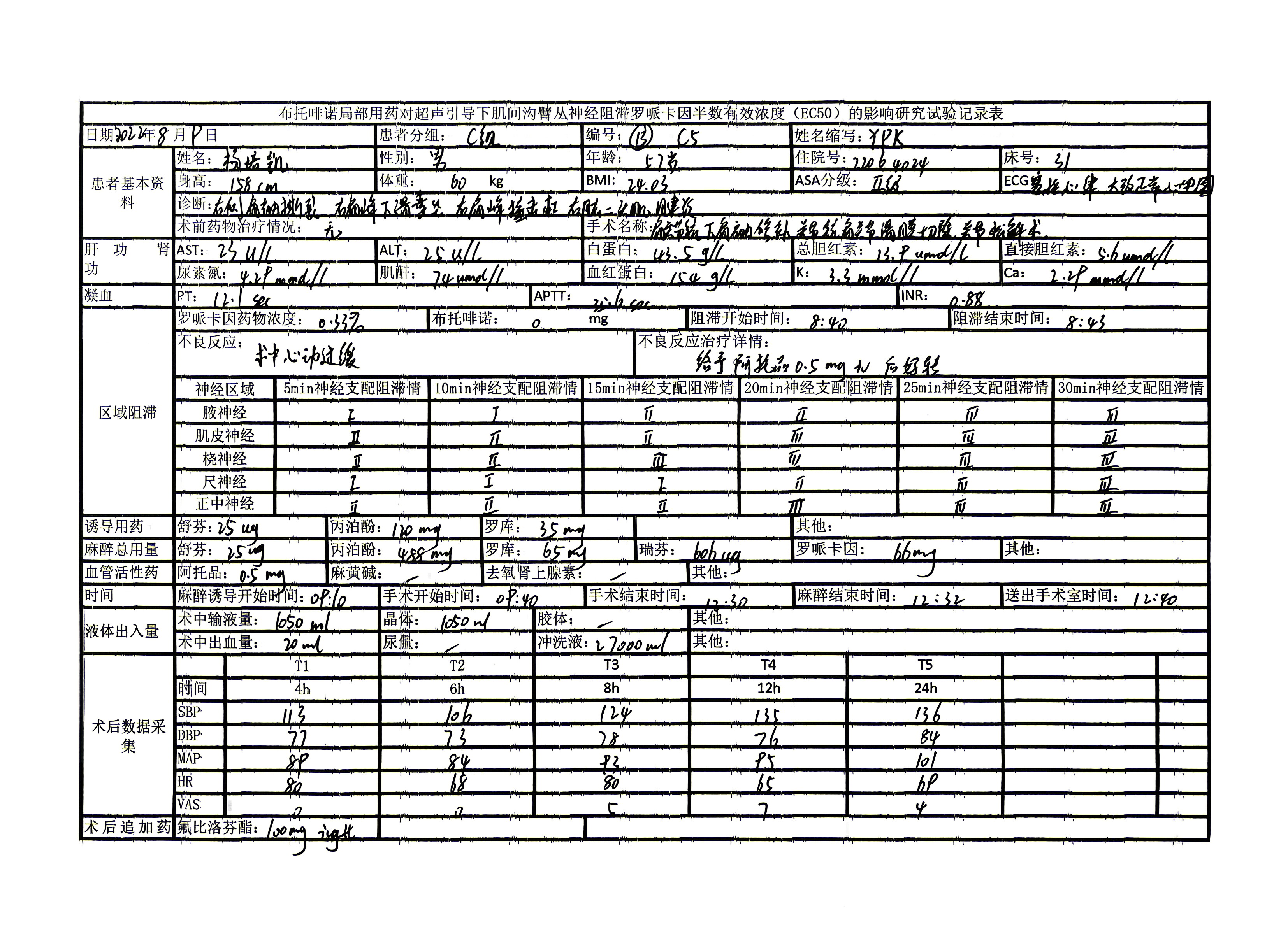

Supplement: S6 File — (ZIP) [file pone.0350613.s010.zip › 028.jpg]

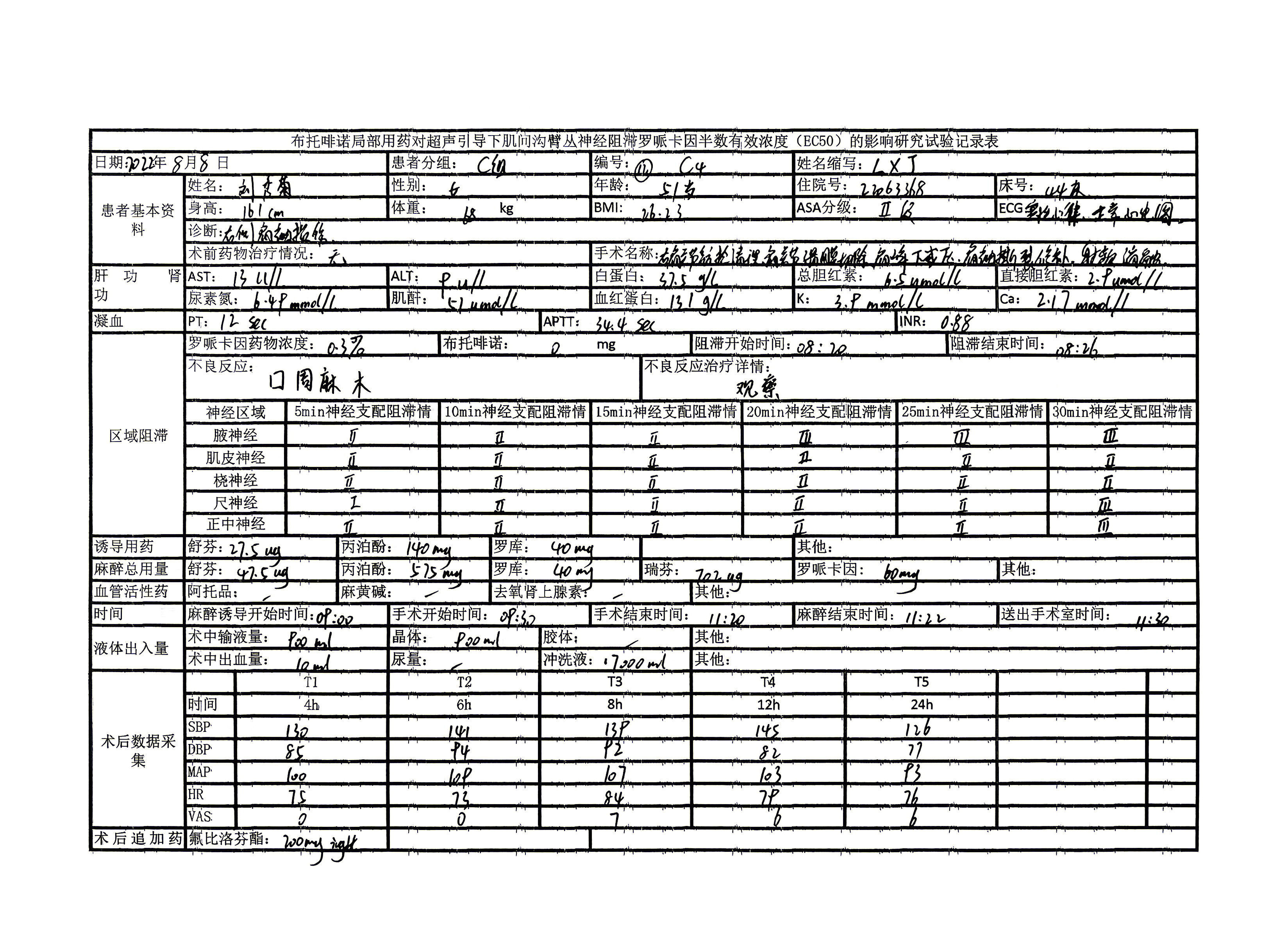

Supplement: S6 File — (ZIP) [file pone.0350613.s010.zip › 029.jpg]

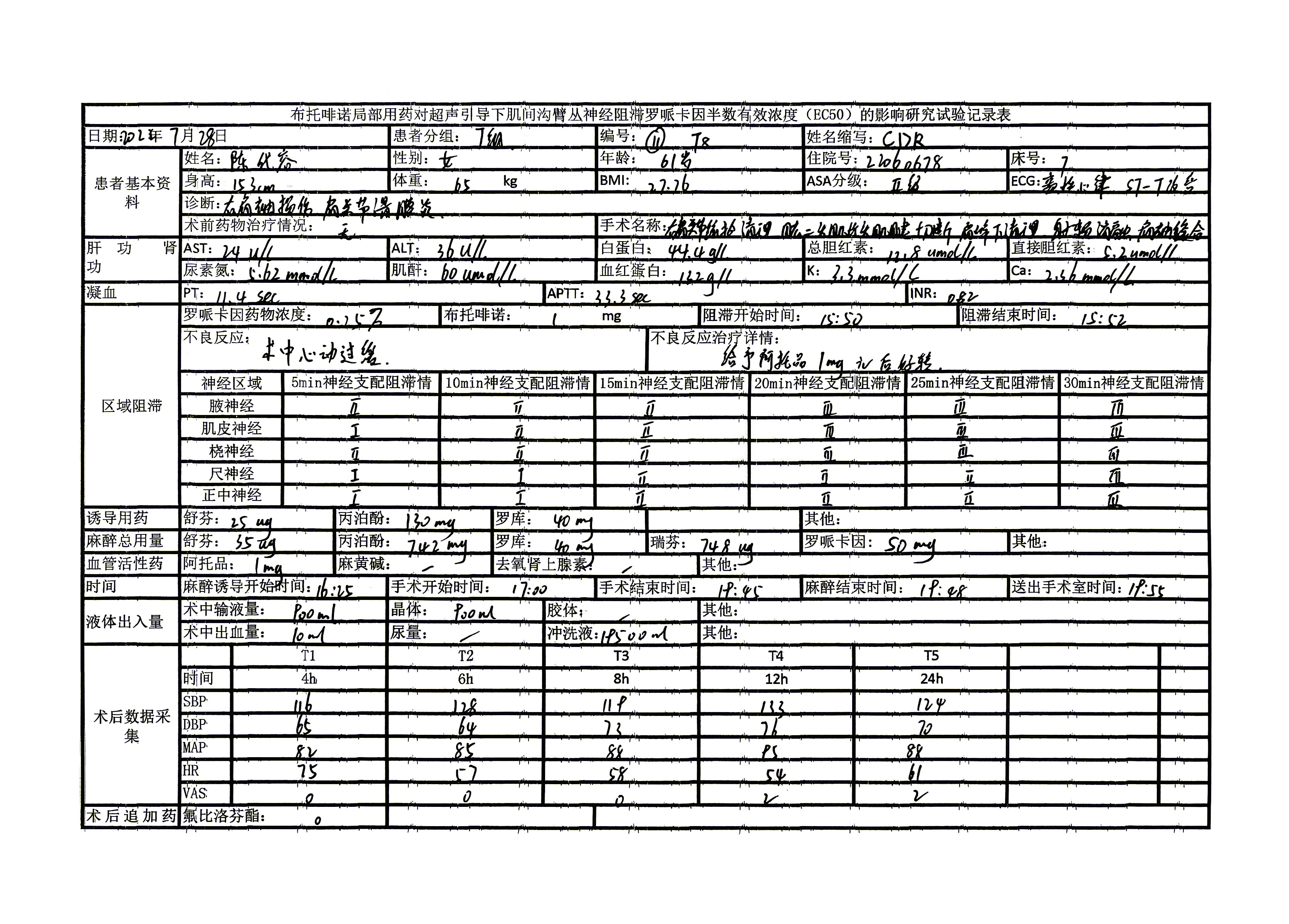

Supplement: S6 File — (ZIP) [file pone.0350613.s010.zip › 030.jpg]

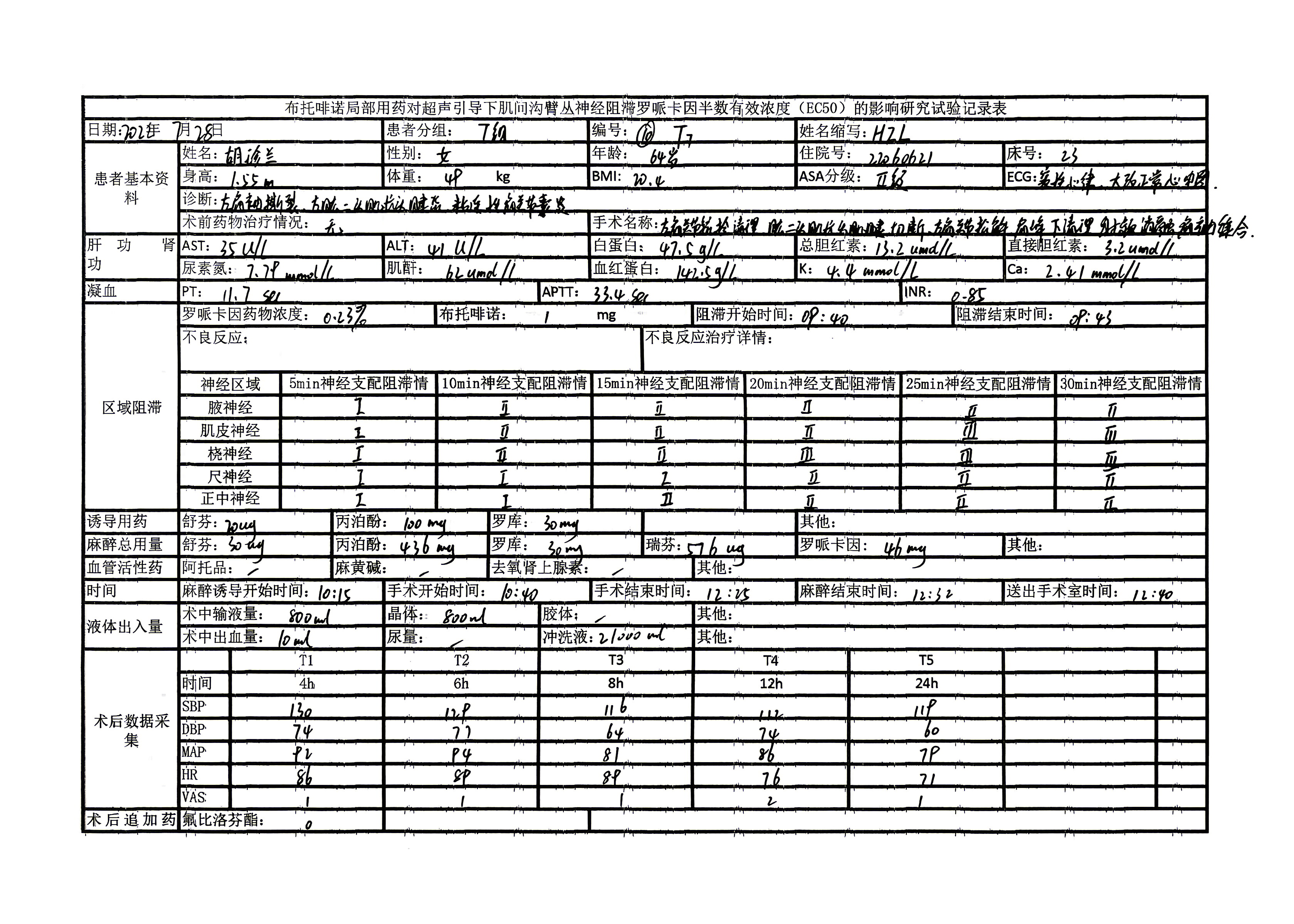

Supplement: S7 File — (ZIP) [file pone.0350613.s011.zip › 031.jpg]

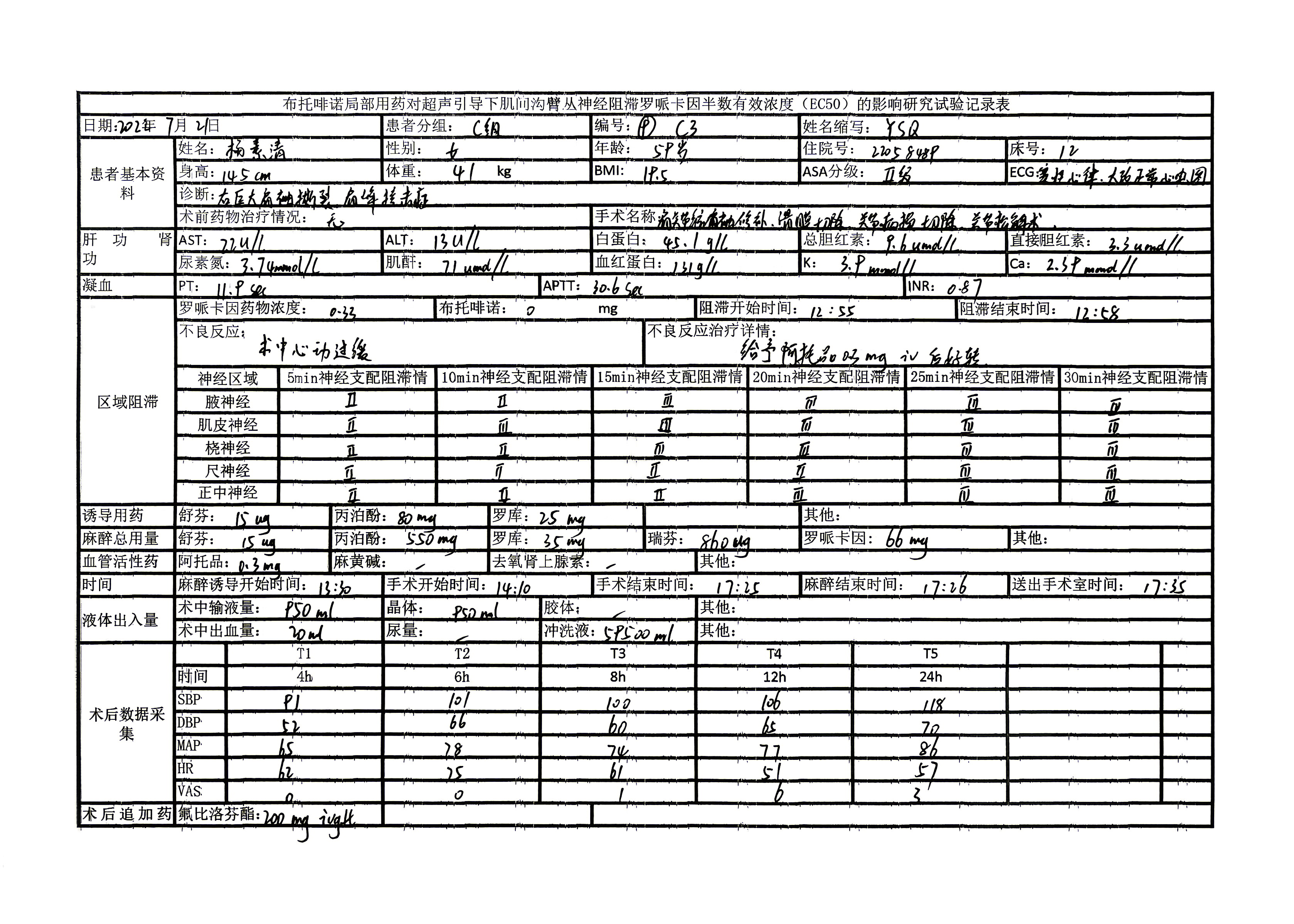

Supplement: S7 File — (ZIP) [file pone.0350613.s011.zip › 032.jpg]

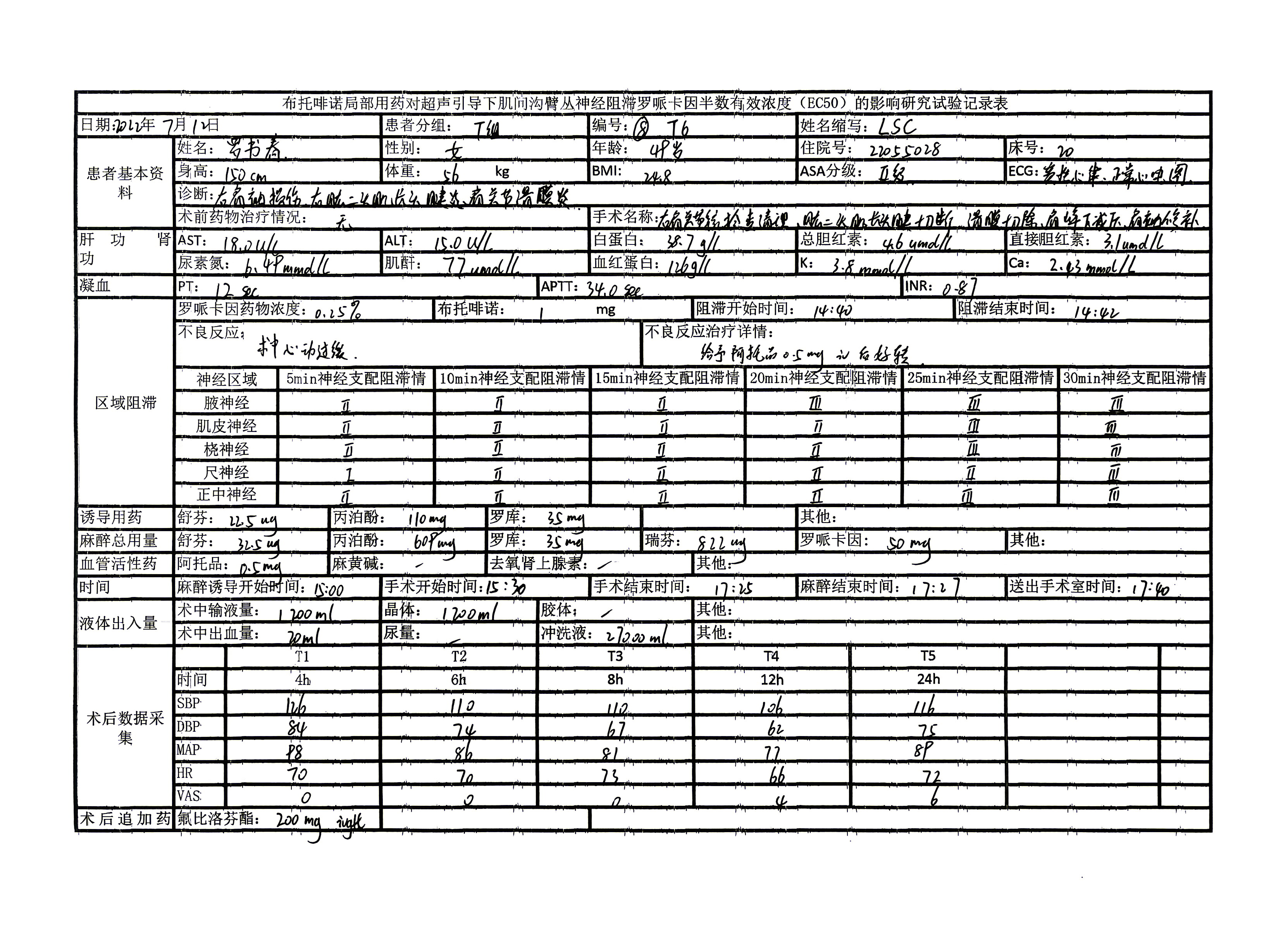

Supplement: S7 File — (ZIP) [file pone.0350613.s011.zip › 033.jpg]

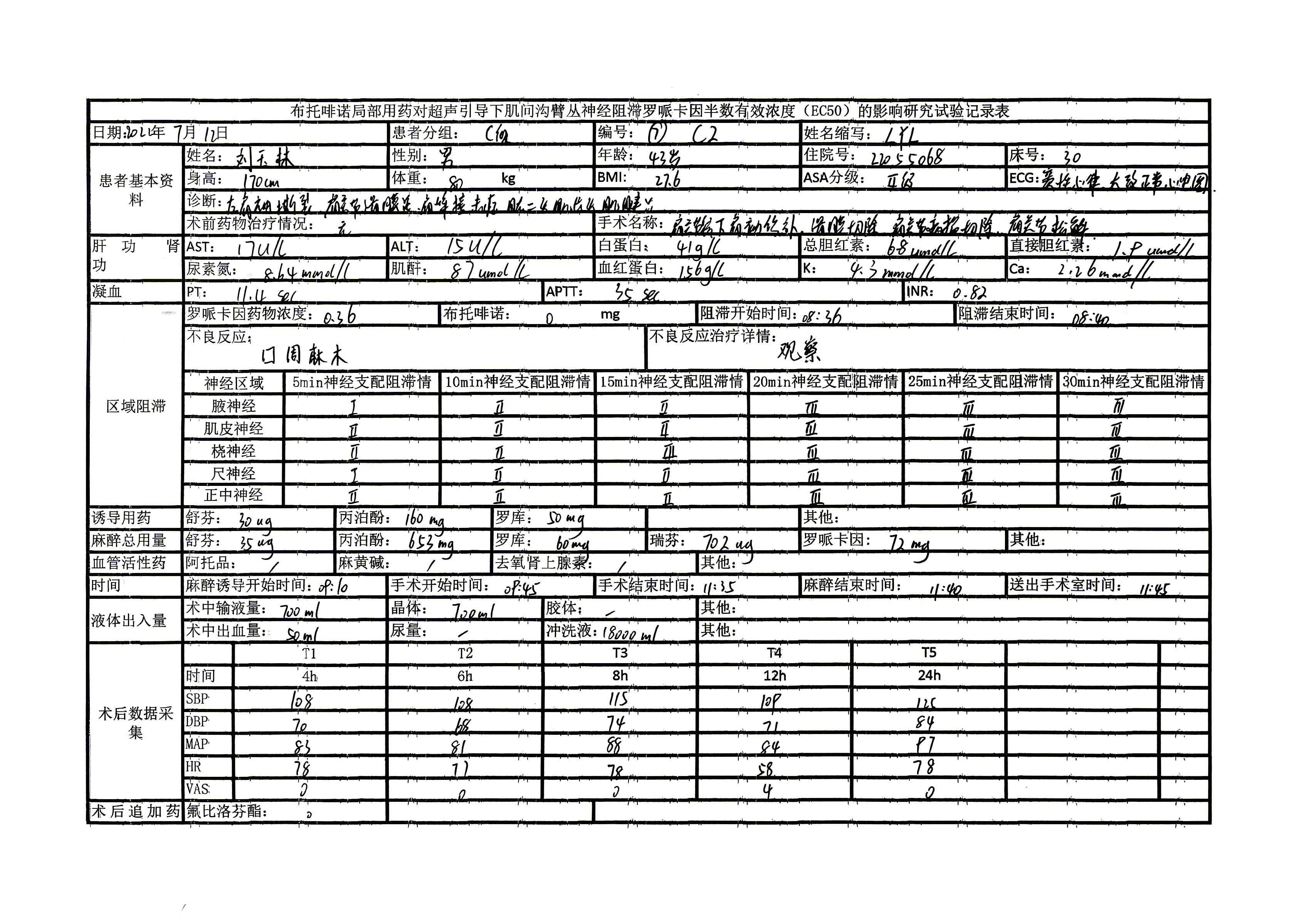

Supplement: S7 File — (ZIP) [file pone.0350613.s011.zip › 034.jpg]

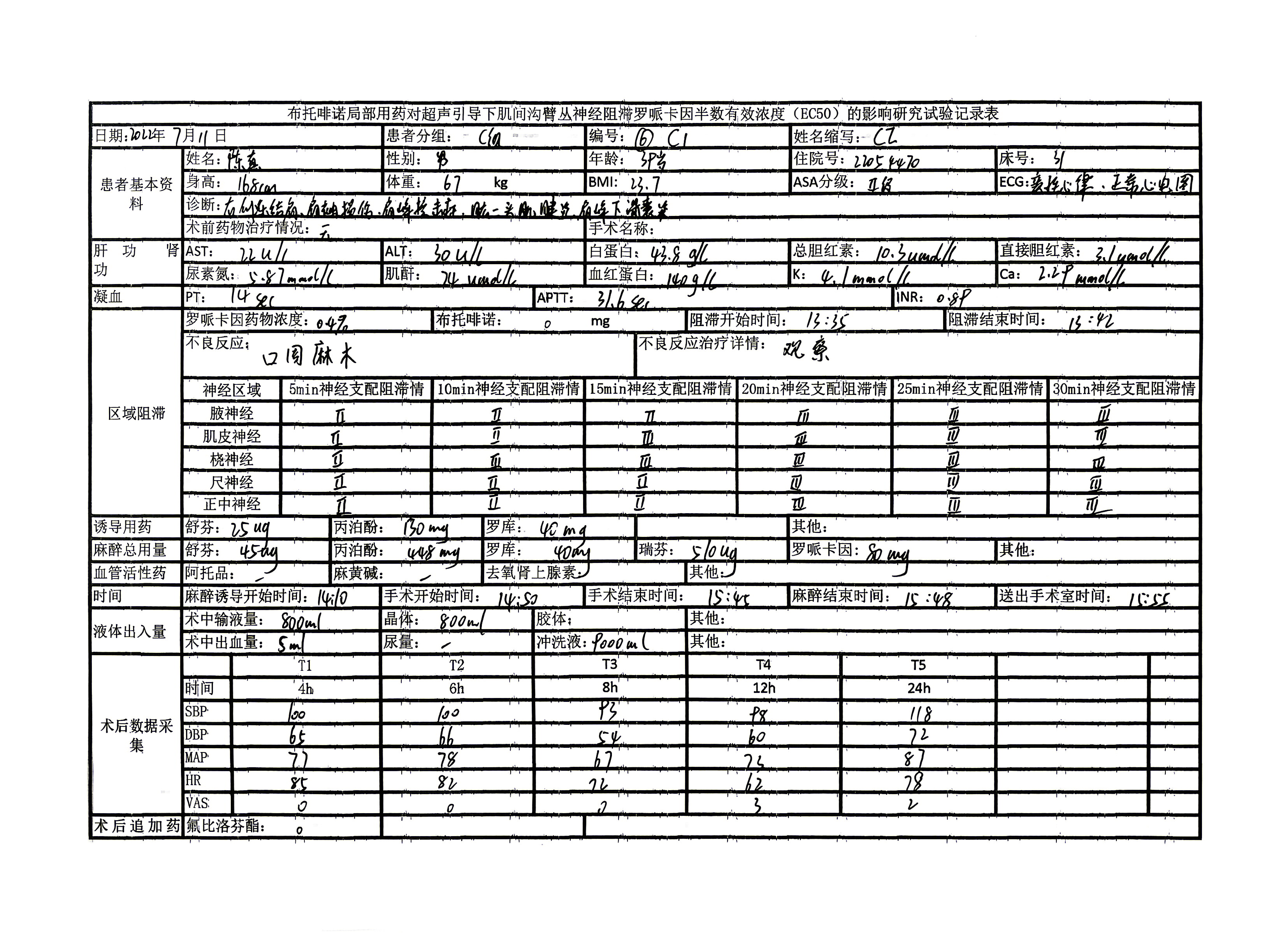

Supplement: S7 File — (ZIP) [file pone.0350613.s011.zip › 035.jpg]

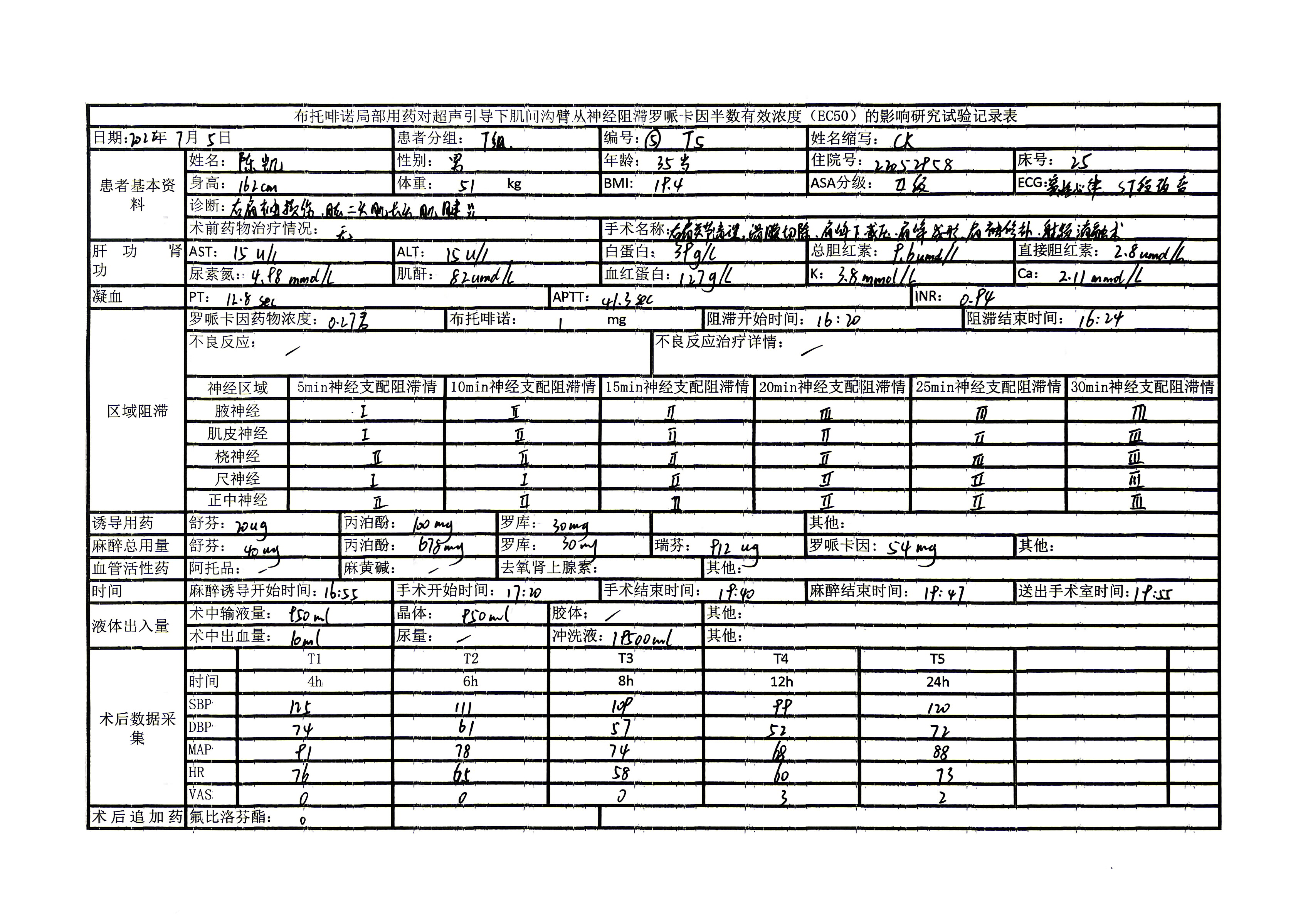

Supplement: S7 File — (ZIP) [file pone.0350613.s011.zip › 036.jpg]

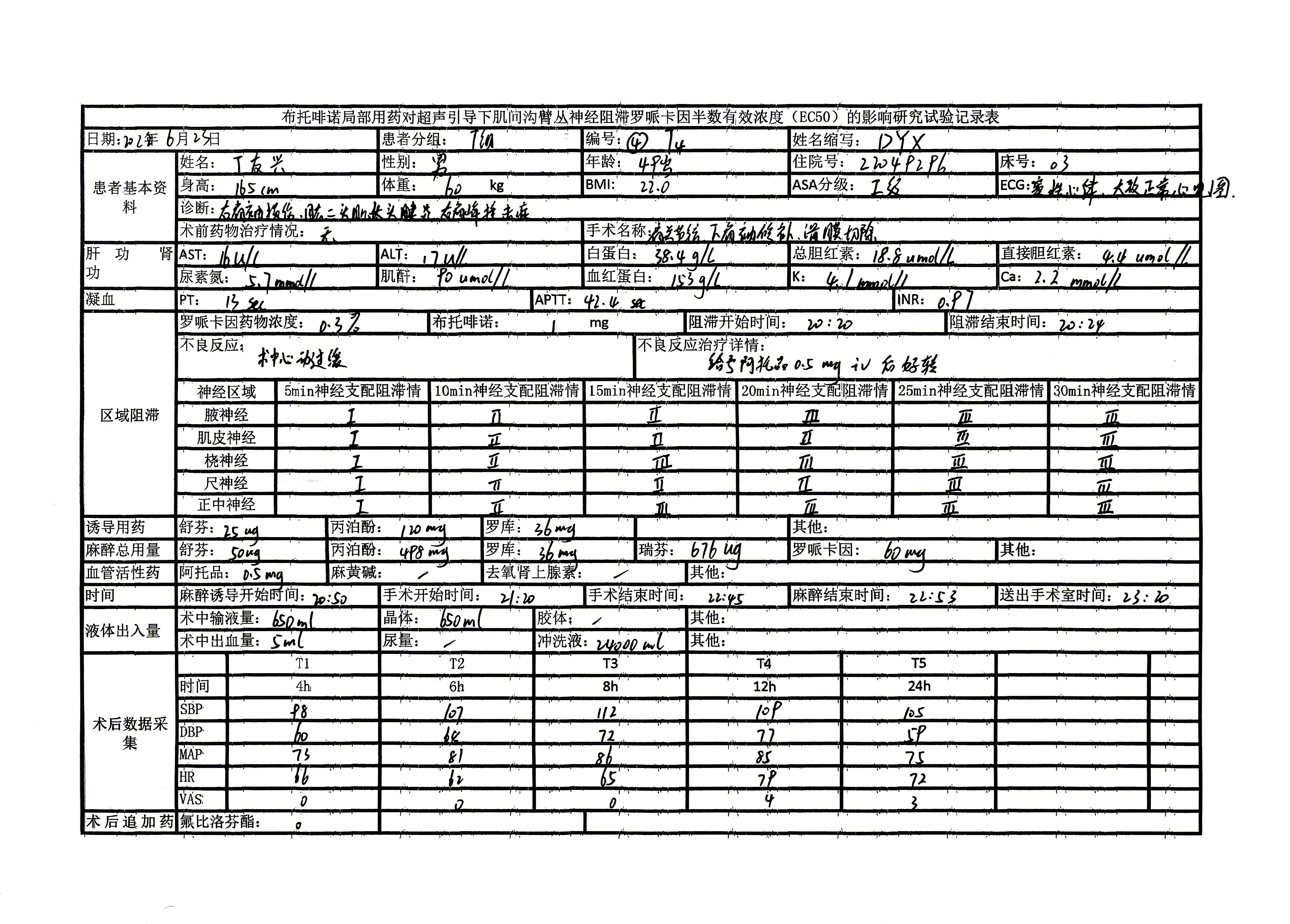

Supplement: S7 File — (ZIP) [file pone.0350613.s011.zip › 037.jpg]

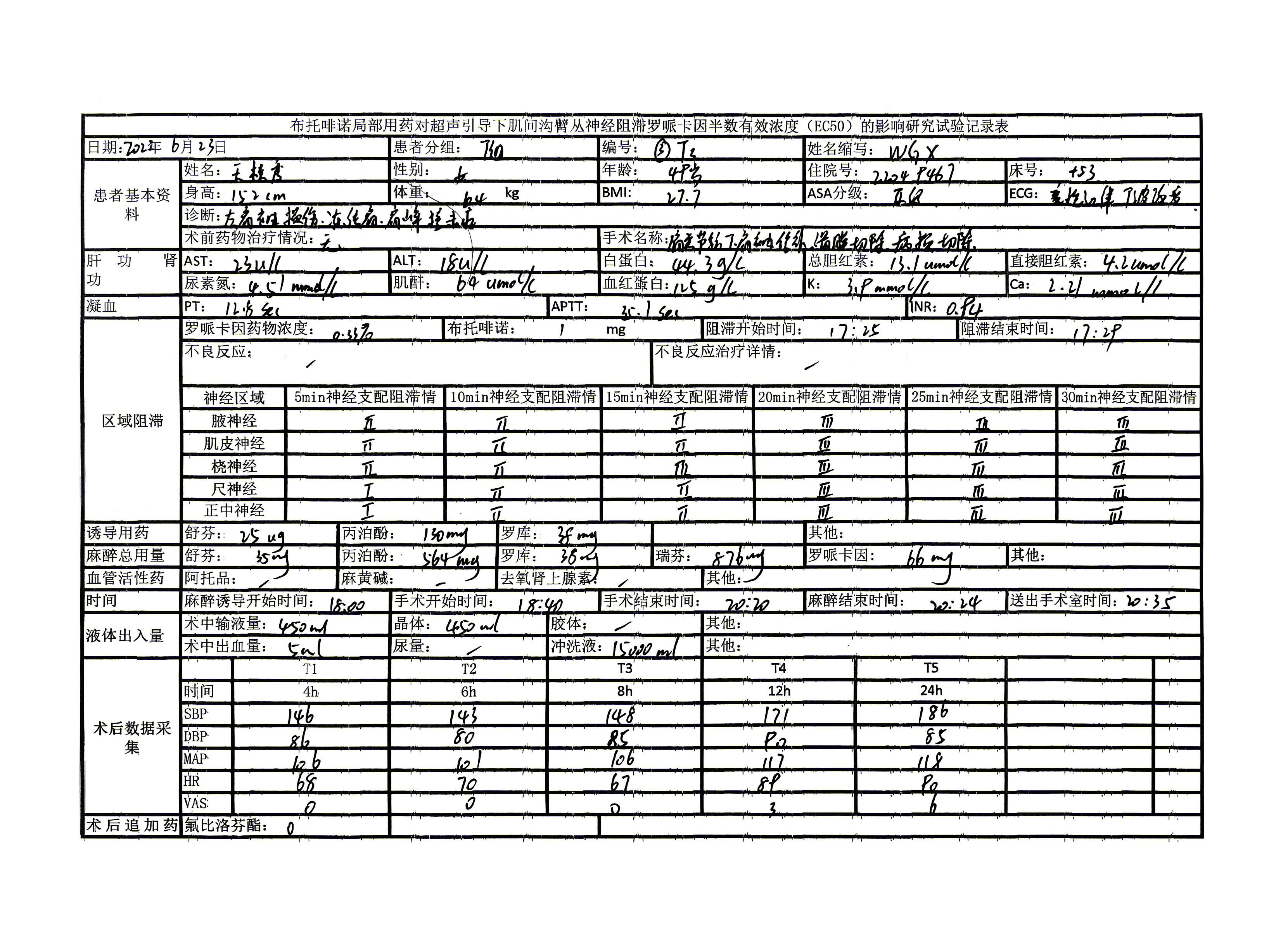

Supplement: S7 File — (ZIP) [file pone.0350613.s011.zip › 038.jpg]

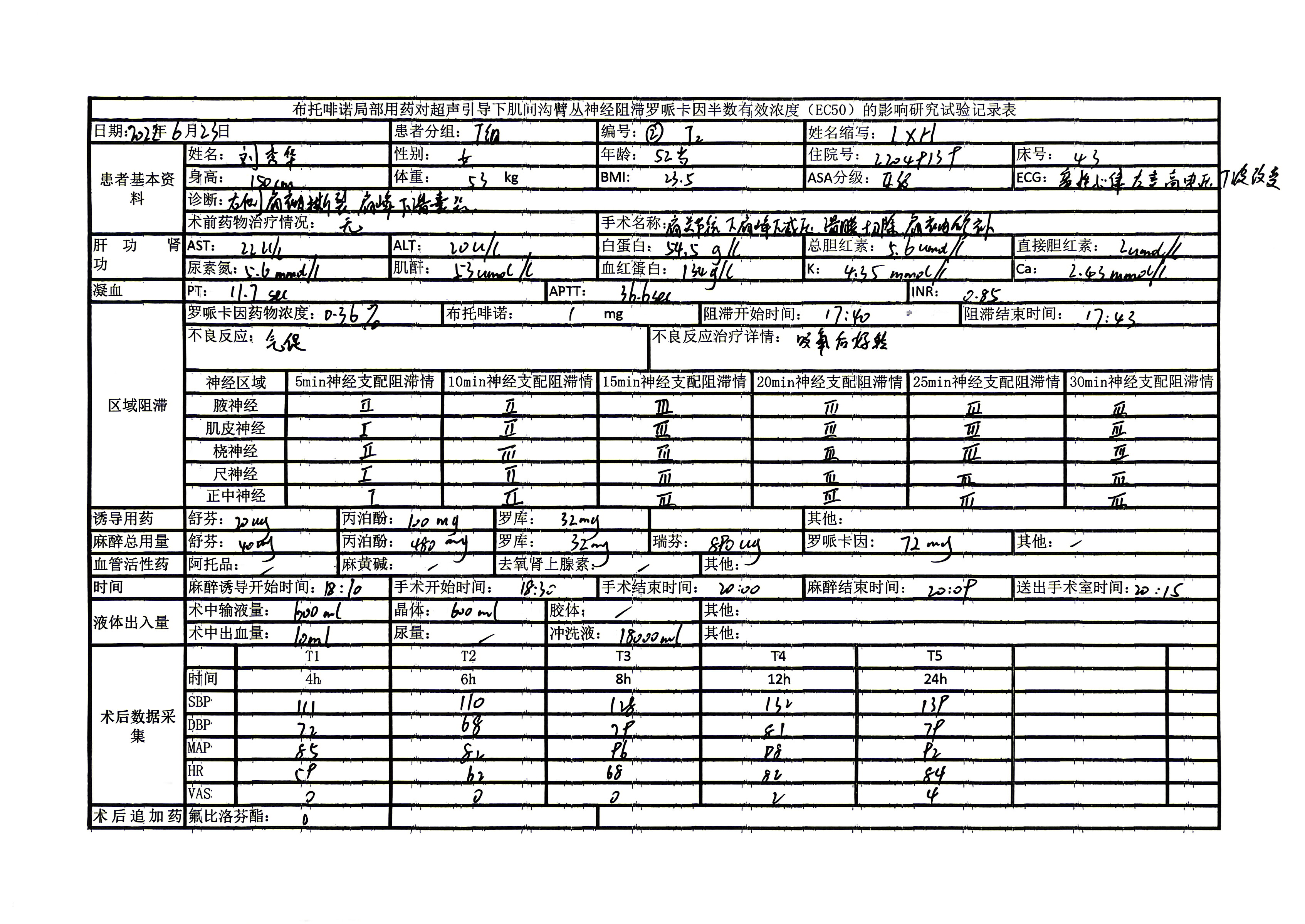

Supplement: S7 File — (ZIP) [file pone.0350613.s011.zip › 039.jpg]

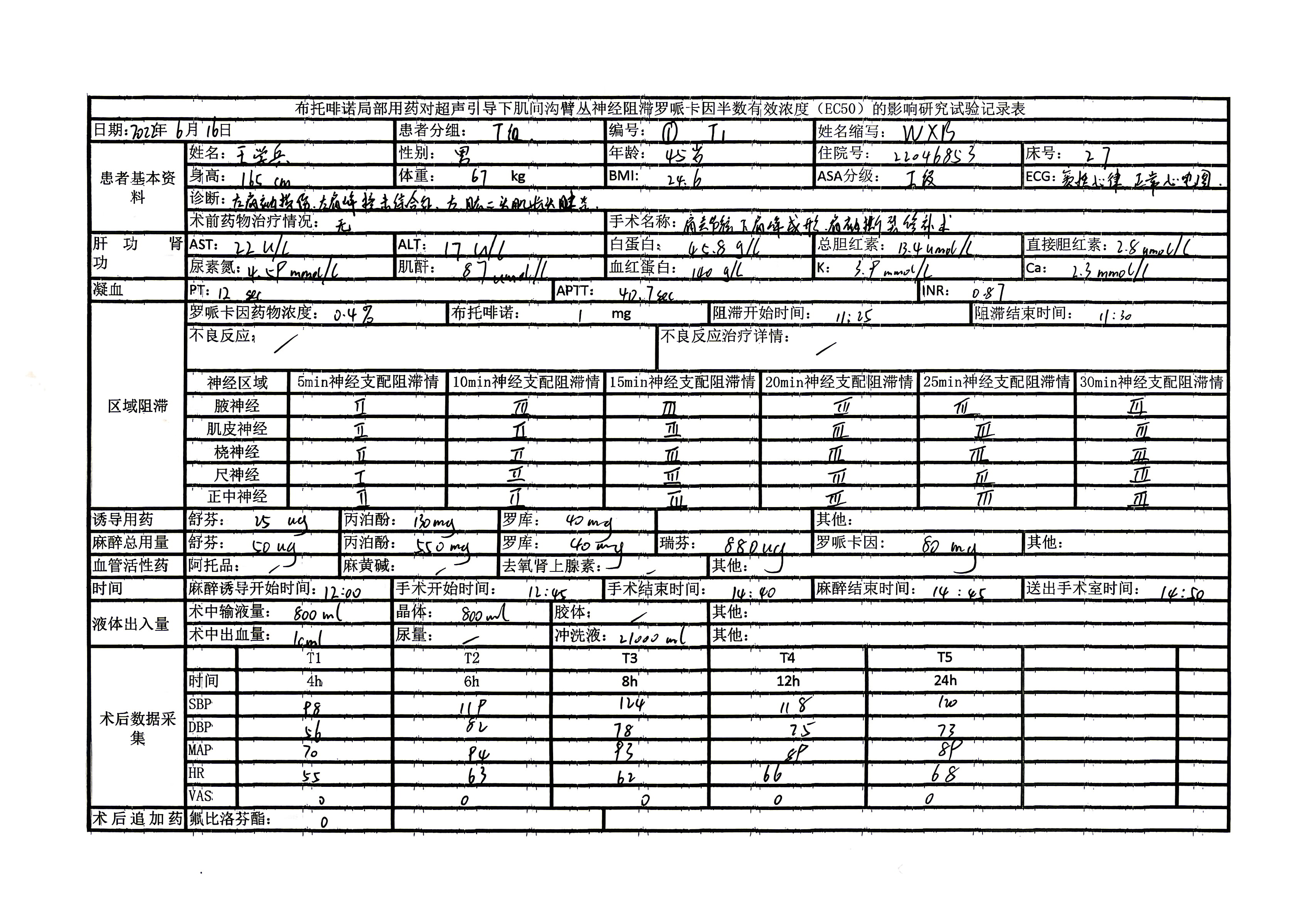

Supplement: S7 File — (ZIP) [file pone.0350613.s011.zip › 040.jpg]
