# Supplementary material for: Butorphanol decreased the median effective concentration of ropivacaine in ultrasound-guided interscalene brachial plexus block
Source: PLoS One. 2026 Jun 16;21(6):e0350613. doi: 10.1371/journal.pone.0350613 (PMC13271508; doi:10.1371/journal.pone.0350613)
Supplement: S2 File — (DOCX) [file pone.0350613.s006.docx]

| **试验方案** |
| --- |
| **一.** **患者选择**  选择在遂宁市中心医院全麻下行肩关节镜手术患者50例，所有患者及家属均签署知情同意书。  1. 纳入标准:  （1）择期全麻下行肩关节镜手术患者  （2）ASA 级I ～ II 级，年龄 20 ～ 65岁，体重指数（BMI）18～28kg/m2；  （3）无镇痛镇静药物滥用史；  （4）能阅读和理解知情同意书内容，并能签署试验知情同意书。  2. 排除标准:  （1）精神、神经系统疾病史，颅脑损伤等不能配合者；  （2）穿刺部位皮肤有感染患者；  （3）凝血功能障碍患者；  （4）对研究药物过敏者；  （5）术前长期服用非甾体抗炎药、麻醉性镇痛药或镇静剂者；  （6）语言、通信障碍或无法理解疼痛评估量表，无法使用病人自控镇痛(PCA)泵者；  （7）糖尿病患者。  3. 退出标准：  在试验进行过程中，若发生下述情况，则需中止试验。对退出试验的原因在观察表上详细记录与评估。   1. 患者在试验进行过程中拒绝配合； 2. 超声引导下臂丛神经阻滞在实施过程中出现失败，包括多次穿刺或注药后药液扩散位置不佳，或出现局麻药中毒等不良反应；   （3）术中出现大出血、心跳骤停等不良事件；  （4）术后随访时间不足或数据收集不完整；  （5）受试者不能遵循试验设定方案中规定的用药要求；  （6）若术后 24h 内患者出现切口部位感染、裂开、持续高烧不退等，或者因其他因素需再次手术者退出试验；  **二．试验分组与研究药物应用方案**  1试验分组：  对50位全麻下行肩关节镜手术的患者进行随机化分组，分成两组：对照组（C 组）和试验组（T 组）：C 组神经阻滞用药仅为罗哌卡因20ml；T 组神经阻滞用药为罗哌卡因和布托啡诺1mg混合液20ml。罗哌卡因浓度由上下序贯法确定，起始浓度为 0.4%，相邻浓度比值为1.1。  由不参与神经阻滞操作和效果评估的手术室护士应用 Excel 软件对50位患者进行随机化分组，分为 C 组和 T 组患者，分组完成后将每个数字对应的组别及干预措施密封后从1开始编号，保证其与纳入患者的顺序一一对应，在试验当天由该护士按照分组进行药物配制，交于进行神经阻滞操作的医生进行超声引导下肌间沟臂丛神经阻滞操作。操作完成后的评估则由对上述过程都不知情的医生进行，且患者及家属对分组情况也不知情。  2 研究药物应用方案：  各组在超声引导下行肌间沟臂丛神经阻滞，采用序贯法开始试验，以20 ml为总量，第1例患者起始浓度为0.4%，相邻浓度比值为1.1。注药后30min内，每10min评价五条神经（肌皮神经、桡神经、正中神经、尺神经、腋神经）支配区痛觉阻滞情况：痛觉正常或稍减退为Ⅰ级，痛觉明显减退为Ⅱ级，痛觉消失为Ⅲ级。感觉阻滞有效定义为：上述神经支配皮区的感觉阻滞程度为Ⅲ级；感觉阻滞无效的定义为：上述任一神经支配皮区的感觉阻滞程度为Ⅰ-Ⅱ级。注药后30min如任一神经支配皮区的感觉阻滞无效，为阳性反应，则下一例患者所用罗哌卡因上调一级浓度；注药后30min如任一神经支配皮区的感觉阻滞有效，为阴性反应，罗哌卡因下调一级浓度。从首次出现阴性反应后第1个阳性反应点开始，将每次从阳性反应到阴性反应的折点作为一个交叉点，直至每组出现第7个交叉点结束。以各交叉点罗哌卡因浓度的均数作为罗哌卡因用于臂丛神经阻滞的EC50。试验终点为达到七个上下周期或者罗哌卡因浓度≤0.1%或者≥1%持续7例。   1. **麻醉方案** 2. 麻醉前行肌间沟臂丛神经阻滞：所有患者术前禁食8小时。患者入麻醉准备间后非手术侧上肢建立静脉通路，常规吸氧，持续监测心率、血压、SpO2。所有患者均去枕平卧，术侧上肢紧贴躯干，头略偏向对侧。首先进行臂丛神经定位，先在超声下寻找肌间沟所在部位，定位臂丛神经具体位置。将超声线阵探头置于环状软骨水平，逐渐向胸锁乳突肌外侧缘移动，在这个过程中于超声下可依次识别出气管、甲状腺、颈动脉、颈内静脉、胸锁乳突肌、前斜角肌和中斜角肌，此时可于前、中斜角肌之间看到数个低回声结构，呈串珠样排列，上下滑动探头，在见到神经干数目最多时即为穿刺理想切面。需要注意的是，臂丛神经根或干超声显示为低回声暗区，与血管结构回声类似，但无搏动、压之闭合等特点，此时，应在穿刺前用超声 Color 模式或多普勒模式进行鉴别，以避免穿刺损伤和局麻药误入血管。当明确判断臂丛神经结构后，对拟穿刺部位进行消毒，在超声探头外适宜位置进行穿刺，选用50mm穿刺针进行穿刺，采用平面内穿刺技术，注意时刻保持针体及针尖位于超声监视下，以避免盲目操作导致的不必要的神经、血管损伤。当看到针尖到达神经周围时，回抽注射器，当无血液、气体及脑脊液返流时，可在神经周围注射实验用药物。需注意，在神经阻滞过程中需不断调整针尖位置，以使药物充分包绕神经纤维。在每次调整位置时均需常规回抽注射器，然后再注射药物，以避免穿刺损伤导致气胸，或穿刺针误入蛛网膜下腔，导致全脊麻，或误入血管导致局麻药中毒。   采用针刺法测定感觉神经阻滞情况，助手采用25G针头碰触神经支配区域（肌皮神经：前臂外侧上中三分之一交界处；桡神经：虎口区；正中神经：大鱼际和食指远端指节指腹，尺神经-小鱼际和小指远端指节指腹；腋神经：肩上部、三角肌区域。）评价阻滞情况（感觉阻滞分3级：痛觉正常或稍减退为Ⅰ级，痛觉明显减退为Ⅱ级，痛觉消失为Ⅲ级），每隔10 min记录神经分布区域的痛觉阻滞情况，如阻滞后 30 min 内针刺患者痛觉Ⅲ级视为有效。  所有操作均由同一位麻醉医师完成，操作完成后的评估和记录则由对上述过程都不知情的麻醉医生进行。   1. 麻醉诱导及术中管理：患者进入手术室后监测心电图（Electrocardiography, ECG）、无创动脉血压（Noninvasive blood pressure, NIBP）心率（Heartrate，HR）和脉搏血氧饱和度（Saturation of pulse oximetry, SpO2）、呼气末二氧化碳分压（PETCO2）、脑电图双频指数（Bispectral Index，BIS）。面罩吸氧3min，氧流量6L/min。吸氧去氮后以舒芬太尼0.4µg/kg、丙泊酚2mg/kg、罗库溴铵0.6 mg/kg静脉诱导，麻醉深度合适后气管插管机械通气，设定潮气量8~10mL/kg，调整呼吸频率使呼气末二氧化碳分压（PETCO2）维持在35~45mmHg。术中泵注丙泊酚和瑞芬太尼维持麻醉，维持BIS 值控制在40~60，血压和心率维持在基础值20%以内，同时维持患者体温在36～37℃之间。如果平均动脉压(MBP) <基线30%，则给予3 mg麻黄碱或去氧肾上腺素40 μg；当MBP>基线30%时，注射5mg乌拉地尔；如果HR <50次/分，注射0.3-0.5mg阿托品；如果HR＞130次/分时，注射艾司洛尔10～20mg。如有必要，这些步骤会重复执行。   手术结束前30min停止泵注瑞芬太尼，并静脉给予舒芬太尼0.2ug/kg。丙泊酚在缝皮结束后停止。手术结束后待患者清醒，自主呼吸恢复良好，循环稳定，拔管后送麻醉后护理单元（PACU）观察1h后送回病房。  术中监测无创血压、血红蛋白氧饱和度(SpO2)、心率、呼吸频率、PETCO2、BIS值。所有监测参数每5分钟记录一次，直到患者转移到PACU。  3.不良事件的处理  在进行神经阻滞过程中，若出现局麻药中毒反应，则立即停止给药，面罩给氧，保持呼吸道通畅，可静脉注射咪达唑仑（0.05～0.1mg/kg）或丙泊酚（1～2mg/kg）。当循环抑制时，应尽快补充血容量或应用血管活性药物（去甲肾上腺素、阿托品、麻黄碱）维持血流动力学稳定；若出现呼吸抑制，应立即面罩加压给氧，进行有效的人工通气，维持SpO2和PaCO2在正常值范围之内；若出现心动过缓（HR＜50 次/分），可给予阿托品 0.05mg/kg；出现恶心呕吐，给予昂丹司琼4mg。  **四. 手术方案：**所有患者均由同一个手术团队行肩关节镜手术。  **五. 数据收集**  1.记录患者性别、年龄、BMI 等一般资料。  2.穿刺后并发症：记录气胸、膈神经麻痹、霍纳综合症、喉返神经阻滞、恶心呕吐、呼吸抑制、心动过缓、局麻药中毒反应等并发症的发生率。  3.两组分别得到的有效/无效反应的序贯例数、各试验浓度组下的有效数（r）和无效数（s）、各组的有效率（p）和计算后的 EC50及 95%CI。  4.两组患者术后 4h（T1）、术后 6h（T2）、术后 8h（T3）、术后 12h（T4）、术后24h（T5）的VAS 评分。  5.两组患者术后各时间点的HR 和 MAP。  6.两组患者使用罗哌卡因总量、术中阿片类药物使用总量和术后24h内需要进行额外镇痛的人数。 |
