# Supplementary material for: Butorphanol decreased the median effective concentration of ropivacaine in ultrasound-guided interscalene brachial plexus block
Source: PLoS One. 2026 Jun 16;21(6):e0350613. doi: 10.1371/journal.pone.0350613 (PMC13271508; doi:10.1371/journal.pone.0350613)
Supplement: S3 File — (DOCX) [file pone.0350613.s007.docx]

| study protocol |
| --- |
| **一.** **Participants**  In this study, we enrolled 50 patients who underwent arthroscopic shoulder surgery under general anesthesia. All participants provided their written informed consent prior to participation in the study.  1. Inclusion criteria:  （1）patients undergoing shoulder arthroscopic surgery under elective general anesthesia  （2）ASA grade I to II, aged 20 to 65 years, body mass index (BMI) 18 to 28kg/m^2^；  （3）No history of analgesic and sedative drug abuse；  （4）Be able to read and understand the contents of the informed consent and sign the informed consent.  2. Exclusion criteria:  （1）History of mental and nervous system disease, craniocerebral injury and other uncooperative persons;；  （2）Patients with skin infection at the puncture site;；  （3）Patients with coagulation disorders;  （4）Individuals who are allergic to the study drug;  （5）Those who take non-steroidal anti-inflammatory drugs, anesthetic analgesics, or sedatives for a long time before surgery;；  （6）Pain assessment scale for language and communication barriers or inability to understand;；  （7）diabetes.  3. Withdraw criteria：   1. The patient refused to cooperate during the trial; 2. Ultrasound guided brachial plexus block may fail during implementation, including poor diffusion of medication after multiple punctures or injections, or adverse reactions such as local anesthetic poisoning; 3. Adverse events such as heavy bleeding and sudden cardiac arrest occurred during the operation; 4. Insufficient postoperative follow-up time or incomplete data collection; 5. The subjects are unable to follow the medication requirements specified in the trial protocol; 6. If the patient experiences incision site infection, rupture, persistent high fever, etc. within 24 hours after surgery, or if another surgery is required due to other factors, the patient should withdraw from the trial;   2、 Experimental grouping and study drug application plan  1. Experimental grouping:  Fifty patients undergoing shoulder arthroscopy surgery under general anesthesia were randomized into two groups: control group (Group C) and experimental group (Group T): Group C received only 20ml of ropivacaine for nerve block, while Group T received 20ml of a mixture of 1mg of ropivacaine and butorphanol for nerve block. The concentration of ropivacaine was determined by a top-down sequential method, with an initial concentration of 0.4% and an adjacent concentration ratio of 1.1.  Fifty patients were randomly divided into Group C and Group T by operating room nurses who did not participate in nerve block operations and effect evaluations. After grouping, each number corresponding to a group and intervention measure was sealed and numbered from 1 to ensure that it corresponded one-to-one with the order of the included patients. On the day of the experiment, the nurse prepared the medication according to the group and handed it over to the doctor who performed the nerve block operation for ultrasound-guided intermuscular groove brachial plexus nerve block operation. The evaluation after the operation is completed is conducted by doctors who are not aware of the above process, and patients and their families are also unaware of the grouping situation.  2. Research drug application plan:  Each group underwent ultrasound-guided intermuscular groove brachial plexus block, starting the experiment using a sequential method with a total volume of 20 ml. The initial concentration of the first patient was 0.4%, and the ratio of adjacent concentrations was 1.1. Within 30 minutes after injection, evaluate the pain block in the innervation areas of five nerves (muscle skin nerve, radial nerve, median nerve, ulnar nerve, axillary nerve) every 10 minutes: if the pain is normal or slightly reduced, it is classified as level I; if the pain is significantly reduced, it is classified as level II; if the pain disappears, it is classified as level III. The effective definition of sensory blockade is: the degree of sensory blockade in the above-mentioned nerve innervated skin area is grade III; The definition of ineffective sensory blockade is that the degree of sensory blockade in any of the above-mentioned nerve innervated skin areas is grade I-II. If the sensory blockade of any nerve innervated skin area is ineffective 30 minutes after injection, it is a positive reaction, and the next patient will use ropivacaine to upregulate the first level concentration; If the sensory blockade of any nerve innervated skin area is effective 30 minutes after injection, it is a negative reaction, and ropivacaine is downregulated to the first level concentration. Starting from the first positive reaction point after the first occurrence of a negative reaction, use each inflection point from positive to negative reaction as a crossover point until the seventh crossover point appears in each group. The average concentration of ropivacaine at each intersection point is used as the EC50 for ropivacaine in brachial plexus block. The endpoint of the experiment is to achieve seven cycles or a concentration of ropivacaine ≤ 0.1% or ≥ 1% for 7 consecutive cases.  3、 Anesthesia plan  1. Muscle groove brachial plexus block before anesthesia: All patients should fast for 8 hours before surgery. After the patient enters the anesthesia preparation room, a venous access is established in the non-surgical upper limb, and routine oxygen inhalation is performed. Heart rate and blood pressure are continuously monitored SpO2。 All patients were supine with their pillows removed, and the upper limb on the operated side was tightly attached to the trunk, with the head slightly tilted towards the opposite side. Firstly, locate the brachial plexus nerve by searching for the location of the intermuscular groove under ultrasound to locate the specific position of the brachial plexus nerve. Place the ultrasound linear array probe at the level of the cricoid cartilage and gradually move it towards the outer edge of the sternocleidomastoid muscle. During this process, the trachea, thyroid gland, carotid artery, internal jugular vein, sternocleidomastoid muscle, anterior scalene muscle, and middle scalene muscle can be identified sequentially under ultrasound. Several hypoechoic structures can be seen between the anterior and middle scalene muscles, arranged in a bead like pattern. Slide the probe up and down, and the ideal section for puncture is when the maximum number of nerve trunks is seen. It should be noted that the brachial plexus nerve root or trunk ultrasound shows a hypoechoic dark area, similar to the echo of vascular structures, but without pulsation, pressure closure, and other characteristics. At this time, ultrasound Color mode or Doppler mode should be used for identification before puncture to avoid puncture damage and local anesthetic entering the blood vessels by mistake. After clearly determining the structure of the brachial plexus nerve, disinfect the intended puncture site and perform the puncture at a suitable position outside the ultrasound probe. Use a 50mm puncture needle for puncture and employ in-plane puncture technique. Pay attention to keeping the needle body and tip under ultrasound monitoring at all times to avoid unnecessary nerve and vascular damage caused by blind operation. When the needle tip reaches the nerve area, withdraw the syringe. When there is no blood, gas, or cerebrospinal fluid reflux, experimental drugs can be injected around the nerve area. It should be noted that during the process of nerve block, the needle tip position needs to be continuously adjusted to ensure that the drug fully wraps around the nerve fibers. Routine withdrawal of the syringe is required every time the position is adjusted, followed by injection of medication, to avoid puncture injury causing pneumothorax, or puncture needle accidentally entering the subarachnoid space, leading to spinal anesthesia, or accidentally entering blood vessels causing local anesthetic poisoning.   1. The sensory nerve block was measured using acupuncture, and the assistant used a 25G needle to touch the nerve innervation area (muscle skin nerve: the junction of the upper and middle third of the outer forearm; radial nerve: the tiger's mouth area; median nerve: the fingertip of the distal phalanx and index finger, ulnar nerve - distal phalanx and little finger; axillary nerve: the upper shoulder and deltoid muscle area) to evaluate the block situation (sensory block is divided into 3 levels: normal or slightly reduced pain sensation is level I, significantly reduced pain sensation is level II, and pain disappearance is level III). The pain block situation in the nerve distribution area was recorded every 10 minutes, and if the patient's pain sensation is level III within 30 minutes after block, it is considered effective.   All operations are performed by the same anesthesiologist, and the evaluation and recording of the completed operations are carried out by anesthesiologists who are not aware of the above process.  Anesthesia induction and intraoperative management: After entering the operating room, patients are monitored for electrocardiography (ECG), non-invasive blood pressure (NIBP), heart rate (HR), saturation of pulse oxygen (SpO2), end expiratory carbon dioxide partial pressure (PETCO2), and bispecific index (BIS) of electroencephalography. Mask oxygen inhalation for 3 minutes, oxygen flow rate 6L/min. After oxygen and nitrogen removal, intravenous induction was performed with sufentanil 0.4 µ g/kg, propofol 2mg/kg, and rocuronium 0.6 mg/kg. After appropriate anesthesia depth, mechanical ventilation was performed through endotracheal intubation with a tidal volume of 8-10 mL/kg. The respiratory rate was adjusted to maintain the end tidal carbon dioxide partial pressure (PETCO2) at 35-45 mmHg. During the operation, propofol and remifentanil were pumped to maintain anesthesia, with BIS values controlled between 40-60, blood pressure and heart rate maintained within 20% of baseline values, and patient temperature maintained between 36-37 ℃. If the mean arterial pressure (MBP) is less than 30% of baseline, administer 3 mg of ephedrine or 40 μ g of epinephrine; When MBP>baseline 30%, inject 5mg of urapidil; If HR<50 times/minute, inject 0.3-0.5mg atropine; If HR>130 times/minute, inject 10-20mg of esolol. If necessary, these steps will be repeated.  Stop pumping remifentanil 30 minutes before the end of the surgery and administer sufentanil 0.2ug/kg intravenously. Propofol is stopped after the suture is completed. After the surgery, wait for the patient to be awake, with good recovery of spontaneous breathing and stable circulation. Remove the tube and send it to the post anesthesia care unit (PACU) for observation for 1 hour before returning to the ward.  Intraoperative monitoring of non-invasive blood pressure, hemoglobin oxygen saturation (SpO2), heart rate, respiratory rate, PETCO2, and BIS values. All monitoring parameters are recorded every 5 minutes until the patient is transferred to the PACU.  3. Handling of adverse events  During the process of nerve block, if there is a local anesthetic poisoning reaction, the administration should be immediately stopped, oxygen should be given through a mask, and the respiratory tract should be kept unobstructed. Midazolam (0.05-0.1mg/kg) or propofol (1-2mg/kg) can be intravenously injected. When circulation is inhibited, blood volume should be replenished as soon as possible or vasoactive drugs (norepinephrine, atropine, ephedrine) should be used to maintain hemodynamic stability; If respiratory depression occurs, immediately apply pressure to oxygen through a face mask and perform effective artificial ventilation to maintain SpO2 and PaCO2 within the normal range; If bradycardia occurs (HR<50 beats/minute), atropine 0.05mg/kg can be administered; If nausea and vomiting occur, administer 4mg of ondansetron. All patients underwent shoulder arthroscopy surgery by the same surgical team.  data collection  1. Record general information such as patient gender, age, BMI, etc.  2. Complications after puncture: Record the incidence of complications such as pneumothorax, diaphragmatic nerve paralysis, Horner's syndrome, recurrent laryngeal nerve block, nausea and vomiting, respiratory depression, bradycardia, and local anesthetic poisoning reactions.  3. The sequential number of effective/ineffective reactions obtained for each group, the effective and ineffective numbers (r) and (s) for each experimental concentration group, the effective rates (p) for each group, and the calculated EC50 and 95% CI.  4. VAS scores of two groups of patients at 4 hours (T1), 6 hours (T2), 8 hours (T3), 12 hours (T4), and 24 hours (T5) after surgery.  5. HR and MAP of two groups of patients at different postoperative time points.  6. The total amount of ropivacaine used by both groups of patients, the total amount of opioid drugs used during surgery, and the number of patients who require additional analgesia within 24 hours after surgery. |
