# Supplementary material for: Butorphanol decreased the median effective concentration of ropivacaine in ultrasound-guided interscalene brachial plexus block
Source: PLoS One. 2026 Jun 16;21(6):e0350613. doi: 10.1371/journal.pone.0350613 (PMC13271508; doi:10.1371/journal.pone.0350613)
Supplement: S8 File — (DOCX) [file pone.0350613.s012.docx]

R group refer to Ropivacaine group. B group refer to Butorphanol group. Ⅱ Ⅰ

Notes:sex，Male： M， Female ：F

| R group：serial number | Age (years) | Sex  (M/F) | Height (cm) | weight (kg) | BMI  (kg/m^2^) | Operation time(min) | ASA |
| --- | --- | --- | --- | --- | --- | --- | --- |
| 1 | 39 | M | 168 | 67 | 23.7 | 55 | Ⅱ |
| 2 | 43 | M | 170 | 80 | 27.6 | 110 | Ⅱ |
| 3 | 59 | F | 145 | 41 | 19.5 | 195 | Ⅱ |
| 4 | 51 | F | 161 | 68 | 26.23 | 110 | Ⅱ |
| 5 | 57 | M | 158 | 60 | 24.03 | 170 | Ⅱ |
| 6 | 51 | F | 165 | 73 | 26.8 | 95 | Ⅱ |
| 7 | 36 | M | 165 | 60 | 22.03 | 80 | Ⅱ |
| 8 | 54 | M | 160 | 63 | 24.6 | 125 | Ⅱ |
| 9 | 56 | F | 150 | 57 | 25.3 | 115 | Ⅱ |
| 10 | 52 | M | 166 | 64.8 | 23.5 | 195 | Ⅱ |
| 11 | 51 | M | 168 | 62.5 | 22.1 | 25 | Ⅱ |
| 12 | 61 | F | 159 | 60 | 23.7 | 85 | Ⅱ |
| 13 | 53 | F | 150 | 51 | 22.6 | 105 | Ⅱ |
| 14 | 42 | F | 160 | 65 | 25.3 | 80 | Ⅱ |
| 15 | 57 | F | 155 | 66 | 27.4 | 140 | Ⅱ |
| 16 | 63 | F | 148 | 40 | 18.2 | 130 | Ⅱ |
| 17 | 58 | F | 158 | 68 | 27.2 | 75 | Ⅱ |
| 18 | 52 | M | 165 | 70 | 25.7 | 100 | Ⅱ |

| B group：serial number | Age (years) | Sex | Height (cm) | weight (kg) | BMI  (kg/m^2^) | Operation time(min) | ASA |
| --- | --- | --- | --- | --- | --- | --- | --- |
| 1 | 45 | M | 165 | 67 | 24.6 | 115 | Ⅰ |
| 2 | 52 | F | 150 | 53 | 23.5 | 90 | Ⅱ |
| 3 | 49 | F | 152 | 64 | 27.7 | 100 | Ⅱ |
| 4 | 49 | M | 165 | 60 | 22 | 85 | Ⅰ |
| 5 | 35 | M | 162 | 51 | 19.4 | 140 | Ⅱ |
| 6 | 49 | F | 150 | 56 | 24.8 | 115 | Ⅱ |
| 7 | 64 | F | 155 | 49 | 20.4 | 105 | Ⅱ |
| 8 | 61 | F | 153 | 65 | 27.76 | 165 | Ⅱ |
| 9 | 36 | M | 167 | 56.5 | 20.2 | 100 | Ⅰ |
| 10 | 26 | M | 173 | 65 | 21.7 | 170 | Ⅱ |
| 11 | 37 | M | 170 | 73 | 25.5 | 85 | Ⅱ |
| 12 | 44 | F | 158 | 51 | 20.4 | 45 | Ⅱ |
| 13 | 52 | M | 155 | 56 | 23.3 | 50 | Ⅱ |
| 14 | 59 | F | 150 | 60 | 26.6 | 215 | Ⅱ |
| 15 | 51 | F | 158 | 65 | 26 | 65 | Ⅱ |
| 16 | 43 | F | 155 | 51 | 21.2 | 135 | Ⅱ |
| 17 | 58 | M | 170 | 59 | 20.4 | 160 | Ⅱ |
| 18 | 54 | F | 156 | 58.2 | 23.9 | 105 | Ⅱ |
| 19 | 48 | M | 161 | 62 | 23.9 | 65 | Ⅱ |
| 20 | 52 | F | 150 | 50 | 21.2 | 225 | Ⅱ |
| 21 | 54 | M | 168 | 55 | 19.4 | 205 | Ⅱ |
| 22 | 53 | F | 155 | 63.5 | 26.4 | 100 | Ⅱ |

Postoperative blood pressure. Data format SBP/DBP, MAP.(mmHg)

| **R group：serial number** | 4h | 6h | 8h | 12h | 24h |
| --- | --- | --- | --- | --- | --- |
| 1 | 100/65 77 | 100/66 78 | 93/54 67 | 98/60 73 | 118/72 87 |
| 2 | 108/70 83 | 108/68 81 | 115/74 88 | 109/71 84 | 125/84 97 |
| 3 | 91/52 65 | 101/66 78 | 100/60 74 | 106/65 77 | 118/70 86 |
| 4 | 130/85 100 | 141/94 109 | 139/92 107 | 145/82 103 | 126/77 93 |
| 5 | 113/77 89 | 106/73 84 | 124/78 93 | 135/76 95 | 136/84 101 |
| 6 | 136/81 99 | 132/80 97 | 120/74 89 | 118/68 84 | 130/78 95 |
| 7 | 114/65 81 | 118/87 97 | 118/86 96 | 96/69 79 | 119/84 95 |
| 8 | 130/80 96 | 120/83 95 | 126/84 98 | 135/84 100 | 114/72 86 |
| 9 | 108/76 86 | 106/70 82 | 106/68 80 | 100/64 76 | 115/74 87 |
| 10 | 122/75 90 | 116/69 84 | 106/85 92 | 105/89 94 | 108/64 79 |
| 11 | 122/64 84 | 121/75 90 | 119/75 89 | 110/76 87 | 122/70 87 |
| 12 | 120/82 94 | 126/76 92 | 118/77 90 | 135/90 105 | 129/89 102 |
| 13 | 156/92 113 | 154/98 116 | 153/92 112 | 130/81 97 | 120/70 87 |
| 14 | 98/65 76 | 102/69 80 | 112/75 87 | 96/66 76 | 126/75 92 |
| 15 | 129/58 81 | 113/59 77 | 107/63 77 | 101/67 78 | 102/51 68 |
| 16 | 144/85 104 | 157/104 121 | 158/92 114 | 154/79 104 | 159/98 118 |
| 17 | 117/80 92 | 121/77 91 | 121/77 91 | 116/74 80 | 120/66 84 |
| 18 | 134/83 100 | 134/85 101 | 139/91 107 | 149/94 112 | 126/74 92 |

| **B group：serial number** | 4h | 6h | 8h | 12h | 24h |
| --- | --- | --- | --- | --- | --- |
| 1 | 98/56 70 | 119/82 94 | 124/78 93 | 118/75 89 | 120/73 89 |
| 2 | 111/72 85 | 110/68 82 | 128/79 96 | 132/81 98 | 139/79 92 |
| 3 | 146/86 106 | 143/80 101 | 148/85 106 | 171/90 117 | 186/85 118 |
| 4 | 98/60 73 | 107/68 81 | 112/72 86 | 109/77 85 | 105/59 75 |
| 5 | 125/74 91 | 111/61 78 | 109/57 74 | 99/52 68 | 120/72 88 |
| 6 | 126/84 98 | 110/74 86 | 110/67 81 | 106/62 77 | 116/75 89 |
| 7 | 130/74 92 | 129/77 94 | 116/64 81 | 112/74 86 | 119/60 79 |
| 8 | 116/65 82 | 128/64 85 | 119/73 88 | 133/76 95 | 124/70 88 |
| 9 | 124/70 88 | 125/69 87 | 128/77 94 | 129/75 93 | 116/67 83 |
| 10 | 116/78 90 | 120/78 92 | 128/83 98 | 122/75 90 | 111/72 85 |
| 11 | 110/63 78 | 105/56 72 | 106/74 84 | 93/56 68 | 110/69 82 |
| 12 | 86/54 65 | 90/50 63 | 89/56 67 | 101/65 77 | 97/64 75 |
| 13 | 121/82 95 | 116/74 88 | 121/79 93 | 115/68 84 | 114/68 83 |
| 14 | 157/85 109 | 156/89 111 | 147/97 113 | 149/81 107 | 137/84 101 |
| 15 | 121/88 99 | 132/80 97 | 114/80 91 | 98/63 74 | 118/85 96 |
| 16 | 104/64 77 | 97/66 76 | 102/51 68 | 96/60 72 | 105/72 83 |
| 17 | 136/86 102 | 110/77 84 | 121/77 91 | 126/65 85 | 124/76 92 |
| 18 | 123/73 89 | 115/67 83 | 120/70 86 | 118/71 86 | 126/65 85 |
| 19 | 122/74 90 | 138/84 102 | 136/84 101 | 133/78 96 | 152/101 91 |
| 20 | 102/62 76 | 126/65 85 | 96/62 73 | 95/61 72 | 110/68 82 |
| 21 | 112/79 90 | 112/67 82 | 133/97 109 | 136/68 91 | 126/58 76 |
| 22 | 115/71 86 | 128/75 92 | 109/72 84 | 112/71 84 | 118/71 86 |

Postoperative HR (beats/min)

| **R group：serial number** | 4h | 6h | 8h | 12h | 24h |
| --- | --- | --- | --- | --- | --- |
| 1 | 85 | 82 | 72 | 62 | 78 |
| 2 | 78 | 77 | 78 | 58 | 78 |
| 3 | 62 | 75 | 61 | 51 | 57 |
| 4 | 75 | 73 | 84 | 79 | 76 |
| 5 | 80 | 68 | 80 | 65 | 69 |
| 6 | 80 | 80 | 82 | 78 | 80 |
| 7 | 65 | 60 | 69 | 57 | 74 |
| 8 | 64 | 64 | 59 | 66 | 66 |
| 9 | 78 | 81 | 84 | 77 | 79 |
| 10 | 68 | 81 | 78 | 84 | 74 |
| 11 | 64 | 86 | 80 | 72 | 80 |
| 12 | 86 | 86 | 72 | 68 | 72 |
| 13 | 76 | 68 | 68 | 63 | 65 |
| 14 | 61 | 71 | 70 | 70 | 80 |
| 15 | 65 | 59 | 67 | 63 | 68 |
| 16 | 82 | 81 | 85 | 85 | 86 |
| 17 | 82 | 85 | 83 | 82 | 82 |
| 18 | 66 | 70 | 63 | 120 | 84 |

| **B group：serial number** | 4h | 6h | 8h | 12h | 24h |
| --- | --- | --- | --- | --- | --- |
| 1 | 55 | 63 | 62 | 66 | 68 |
| 2 | 59 | 62 | 68 | 82 | 84 |
| 3 | 68 | 70 | 67 | 89 | 90 |
| 4 | 66 | 62 | 65 | 79 | 72 |
| 5 | 76 | 65 | 58 | 60 | 73 |
| 6 | 70 | 70 | 73 | 66 | 72 |
| 7 | 86 | 89 | 89 | 76 | 71 |
| 8 | 75 | 57 | 58 | 54 | 61 |
| 9 | 54 | 78 | 74 | 60 | 72 |
| 10 | 72 | 75 | 75 | 56 | 63 |
| 11 | 65 | 74 | 70 | 80 | 74 |
| 12 | 66 | 58 | 68 | 78 | 85 |
| 13 | 70 | 69 | 62 | 67 | 74 |
| 14 | 85 | 60 | 63 | 61 | 68 |
| 15 | 78 | 78 | 82 | 83 | 79 |
| 16 | 62 | 77 | 81 | 81 | 70 |
| 17 | 71 | 71 | 76 | 72 | 71 |
| 18 | 62 | 56 | 59 | 51 | 62 |
| 19 | 79 | 98 | 93 | 93 | 112 |
| 20 | 89 | 85 | 79 | 82 | 83 |
| 21 | 82 | 82 | 80 | 76 | 76 |
| 22 | 87 | 82 | 85 | 79 | 89 |

Dosage of drugs

| R group：serial number | Sufentanil (µg) | Propofol (mg) | Rocuronium Bromide (mg) | Remifentanil (µg) | Ropivacaine (mg) |
| --- | --- | --- | --- | --- | --- |
| 1 | 45 | 448 | 40 | 510 | 80 |
| 2 | 35 | 653 | 60 | 702 | 72 |
| 3 | 15 | 550 | 35 | 860 | 66 |
| 4 | 47.5 | 575 | 40 | 702 | 60 |
| 5 | 25 | 488 | 65 | 606 | 66 |
| 6 | 40 | 523 | 45 | 520 | 60 |
| 7 | 35 | 416 | 35 | 444 | 66 |
| 8 | 45 | 540 | 40 | 672 | 60 |
| 9 | 32.5 | 639 | 35 | 764 | 66 |
| 10 | 45 | 785 | 40 | 1190 | 60 |
| 11 | 35 | 756 | 40 | 944 | 66 |
| 12 | 35 | 404 | 35 | 410 | 60 |
| 13 | 30 | 544 | 30 | 928 | 66 |
| 14 | 35 | 452 | 40 | 496 | 60 |
| 15 | 35 | 712 | 40 | 768 | 54 |
| 16 | 25 | 432 | 25 | 664 | 60 |
| 17 | 37.5 | 522 | 40 | 756 | 54 |
| 18 | 37.5 | 472 | 40 | 636 | 60 |

| B group：serial number | Sufentanil (µg) | Propofol (mg) | Rocuronium Bromide (mg) | Remifentanil (µg) | Ropivacaine (mg) |
| --- | --- | --- | --- | --- | --- |
| 1 | 50 | 550 | 40 | 880 | 80 |
| 2 | 40 | 480 | 32 | 890 | 72 |
| 3 | 35 | 564 | 38 | 876 | 66 |
| 4 | 50 | 498 | 36 | 676 | 60 |
| 5 | 40 | 678 | 30 | 912 | 54 |
| 6 | 32.5 | 609 | 35 | 822 | 50 |
| 7 | 30 | 436 | 30 | 576 | 46 |
| 8 | 35 | 742 | 40 | 748 | 50 |
| 9 | 37.5 | 502 | 30 | 674 | 46 |
| 10 | 45 | 875 | 40 | 1132 | 50 |
| 11 | 40 | 478 | 45 | 566 | 46 |
| 12 | 30 | 326 | 30 | 432 | 50 |
| 13 | 32.5 | 286 | 35 | 320 | 46 |
| 14 | 35 | 1062 | 35 | 1264 | 42 |
| 15 | 35 | 412 | 50 | 402 | 46 |
| 16 | 30 | 550 | 40 | 736 | 50 |
| 17 | 35 | 703 | 35 | 938 | 46 |
| 18 | 32.5 | 503 | 35 | 504 | 50 |
| 19 | 35 | 437 | 40 | 500 | 46 |
| 20 | 30 | 780 | 30 | 660 | 50 |
| 21 | 32.5 | 1060 | 100 | 1020 | 46 |
| 22 | 35 | 677 | 50 | 322 | 50 |

Drug concentration and Individual response

| R group：serial number | Ropivacaine group（%） | B group：serial number | butorphanol group（%） |  |
| --- | --- | --- | --- | --- |
| 1 | 0.4/1 | 1 | 0.4/1 |  |
| 2 | 0.36/1 | 2 | 0.36/1 |  |
| 3 | 0.33/1 | 3 | 0.33/1 |  |
| 4 | 0.3/0 | 4 | 0.3/1 |  |
| E1 | 0.33/0 | 5 | 0.27/1 |  |
| 5 | 0.33/1 | 6 | 0.25/1 |  |
| 6 | 0.3/0 | 7 | 0.23/0 |  |
| 7 | 0.33/1 | 8 | 0.25/1 |  |
| 8 | 0.3/0 | 9 | 0.23/0 |  |
| 9 | 0.33/1 | 10 | 0.25/1 |  |
| 10 | 0.3/0 | 11 | 0.23/0 |  |
| 11 | 0.33/1 | 12 | 0.25/1 |  |
| E2 | 0.3/1 | 13 | 0.23/1 |  |
| 12 | 0.3/0 | 14 | 0.21/0 |  |
| 13 | 0.33/1 | 15 | 0.23/0 |  |
| 14 | 0.3/1 | 16 | 0.25/1 |  |
| E3 | 0.27/0 | 17 | 0.23/0 |  |
| 15 | 0.27/0 | 18 | 0.25/1 |  |
| 16 | 0.3/1 | 19 | 0.23/0 |  |
| 17 | 0.27/0 | 20 | 0.25/1 |  |
| E4 | 0.3/1 | 21 | 0.23/0 |  |
| 18 | 0.3/1 | 22 | 0.25/1 |  |

E1-E4 refer to the 4 excluded patients. “0” refers to negative response,”1” refers to positive response

Postoperative VAS

| **R group：serial number** | 4h | 6h | 8h | 12h | 24h |
| --- | --- | --- | --- | --- | --- |
| 1 | 0 | 0 | 0 | 3 | 2 |
| 2 | 0 | 0 | 0 | 4 | 0 |
| 3 | 0 | 0 | 1 | 6 | 3 |
| 4 | 0 | 0 | 7 | 6 | 6 |
| 5 | 0 | 0 | 5 | 7 | 4 |
| 6 | 0 | 2 | 4 | 6 | 3 |
| 7 | 0 | 0 | 3 | 7 | 3 |
| 8 | 2 | 7 | 7 | 5 | 4 |
| 9 | 0 | 0 | 3 | 7 | 4 |
| 10 | 0 | 0 | 2 | 6 | 1 |
| 11 | 0 | 0 | 6 | 5 | 2 |
| 12 | 0 | 0 | 7 | 5 | 4 |
| 13 | 0 | 0 | 4 | 6 | 2 |
| 14 | 0 | 0 | 4 | 8 | 3 |
| 15 | 0 | 0 | 4 | 6 | 3 |
| 16 | 0 | 0 | 3 | 7 | 5 |
| 17 | 0 | 0 | 4 | 6 | 4 |
| 18 | 0 | 0 | 3 | 6 | 4 |

| **B group：serial number** | 4h | 6h | 8h | 12h | 24h |
| --- | --- | --- | --- | --- | --- |
| 1 | 0 | 0 | 0 | 0 | 0 |
| 2 | 0 | 0 | 0 | 2 | 4 |
| 3 | 0 | 0 | 0 | 3 | 6 |
| 4 | 0 | 0 | 0 | 4 | 3 |
| 5 | 0 | 0 | 0 | 3 | 2 |
| 6 | 0 | 0 | 0 | 4 | 6 |
| 7 | 1 | 1 | 1 | 2 | 1 |
| 8 | 0 | 0 | 0 | 2 | 2 |
| 9 | 0 | 0 | 0 | 1 | 1 |
| 10 | 0 | 0 | 1 | 3 | 4 |
| 11 | 0 | 0 | 0 | 3 | 2 |
| 12 | 0 | 0 | 0 | 3 | 2 |
| 13 | 0 | 0 | 0 | 2 | 1 |
| 14 | 0 | 0 | 3 | 3 | 4 |
| 15 | 0 | 0 | 1 | 3 | 4 |
| 16 | 0 | 0 | 3 | 4 | 3 |
| 17 | 0 | 0 | 0 | 4 | 4 |
| 18 | 0 | 0 | 0 | 3 | 3 |
| 19 | 0 | 0 | 0 | 3 | 2 |
| 20 | 0 | 0 | 2 | 4 | 3 |
| 21 | 0 | 0 | 2 | 3 | 3 |
| 22 | 0 | 0 | 0 | 2 | 4 |

Rescue analgesia

'0' refers to no adverse reactions occurred, ”1” refers to adverse reactions occurred

| **R group：serial number** | Adverse reactions related to nerve block | Adverse reactions related to Surgical |
| --- | --- | --- |
| 1 | 1 Perioral numbness | 0 |
| 2 | 1 Perioral numbness | 0 |
| 3 | 0 | 1 Bradycardia |
| 4 | 1 Perioral numbness | 0 |
| 5 | 0 | 1 Bradycardia |
| 6 | 1 Vomiting | 0 |
| 7 | 0 | 0 |
| 8 | 0 | 1 Hypotension |
| 9 | 1 Dyspnea | 0 |
| 10 | 0 | 0 |
| 11 | 0 | 1 Bradycardia |
| 12 | 0 | 0 |
| 13 | 0 | 0 |
| 14 | 0 | 0 |
| 15 | 0 | 0 |
| 16 | 1 Dyspnea | 1 Bradycardia |
| 17 | 0 | 0 |
| 18 | 0 | 0 |

| **B group：serial number** | Adverse reactions related to nerve block | Adverse reactions related to Surgical |
| --- | --- | --- |
| 1 | 0 | 0 |
| 2 | 1 Dyspnea | 0 |
| 3 | 0 | 0 |
| 4 | 0 | 1 Bradycardia |
| 5 | 0 | 0 |
| 6 | 0 | 1 Bradycardia |
| 7 | 0 | 0 |
| 8 | 0 | 1 Bradycardia |
| 9 | 0 | 0 |
| 10 | 0 | 0 |
| 11 | 0 | 0 |
| 12 | 0 | 0 |
| 13 | 0 | 0 |
| 14 | 0 | 1 Bradycardia |
| 15 | 0 | 0 |
| 16 | 0 | 0 |
| 17 | 0 | 0 |
| 18 | 0 | 0 |
| 19 | 0 | 0 |
| 20 | 0 | 0 |
| 21 | 0 | 0 |
| 22 | 0 | 0 |

Rescue analgesia. '0' indicates that no rescue analgesia has been taken. '1' indicates that rescue analgesia has been taken.

| **serial number** | R group | B group |
| --- | --- | --- |
| 1 | 0 | 0 |
| 2 | 0 | 0 |
| 3 | 1 | 0 |
| 4 | 1 | 0 |
| 5 | 1 | 0 |
| 6 | 1 | 1 |
| 7 | 0 | 0 |
| 8 | 1 | 0 |
| 9 | 0 | 0 |
| 10 | 1 | 0 |
| 11 | 1 | 0 |
| 12 | 1 | 0 |
| 13 | 1 | 0 |
| 14 | 1 | 0 |
| 15 | 1 | 0 |
| 16 | 1 | 0 |
| 17 | 1 | 0 |
| 18 | 1 | 0 |
| 19 |  | 1 |
| 20 |  | 0 |
| 21 |  | 1 |
| 22 |  | 0 |
