# Supplementary material for: Butorphanol decreased the median effective concentration of ropivacaine in ultrasound-guided interscalene brachial plexus block
Source: PLoS One. 2026 Jun 16;21(6):e0350613. doi: 10.1371/journal.pone.0350613 (PMC13271508; doi:10.1371/journal.pone.0350613)
Supplement: S9 File — (PDF) [file pone.0350613.s013.pdf]

Medical Research Ethics Review Committee of Suining Central Hospital
Initial Review

Project Name<br>（Acceptance Number）	Effect of local butorphano on median effective concentration (EC50) of ropivacaine in ultrasound-guided intermuscular sulcus brachial plexus block<br>Acceptance Number:LLSLH20220029
Applying Department	Surgical Anesthesia Department I
Name of project leader	Juan Li
reason for review	Initial Review
review method	☐ Meeting review ☒ Rapid Assessment
review documents	1. Suining Central Hospital Scientific Research Ethics Review Application Form<br>2. Informed consent form
Examination Opinion: After preliminary review by two experts from this ethics committee, it is agreed to proceed with the study	

Precautions:
All materials shall not be modified without the review and approval of the committee;
If any serious adverse event occurs during the research process, please notify this ethics committee within 24 hours of becoming aware of it;
If any deviation from the research protocol occurs during the research process, a protocol deviation report should be submitted promptly;
From the date of approval, an annual report must be submitted to the ethics committee every year. Please submit the application and report for annual review one month before the review date;
If the research is suspended or terminated early, a timely application for suspension/termination review should be submitted;
At the end of the study, a conclusion report should be submitted to the ethics committee;
This certificate is solely intended for use as proof of ethical compliance in scientific research

Signature of the Chairman/Vice Chairman: Date: June 8, 2022

Medical Research Ethics Review Committee of Suining Central Hospital（Seal here）
