# Supplementary material for: Butorphanol decreased the median effective concentration of ropivacaine in ultrasound-guided interscalene brachial plexus block
Source: PLoS One. 2026 Jun 16;21(6):e0350613. doi: 10.1371/journal.pone.0350613 (PMC13271508; doi:10.1371/journal.pone.0350613)
Supplement: Attachment — Submitted filename: Response to Reviewers.docx [file pone.0350613.s015.docx]

**Response Letter**

Dear editor:

Thank you very much for the editorial staff and reviewer’s constructive comments and suggestions on our manuscript titled “Butorphanol decreased the median effective concentration of ropivacaine in ultrasound-guided interscalene brachial plexus block”. Your comments and those from the reviewers were highly insightful and allowed us to improve the quality of our manuscript. We have carefully considered all comments and provided point-by-point responses to the comments below, and all revisions are shown in highlights in the revised manuscript.

We have resubmitted the revised manuscript for your consideration for publication in *PLOS One* We hope that we have satisfactorily addressed the editorial staff’s and reviewers’ questions and comments. We deeply appreciate your and reviewers’ careful review of our manuscript. If you have any further questions, please feel free to contact me at my email address: tfpnc@163.com.

Yours sincerely,

Faping Tu

**Response to Journal academic editor**

**1. Thank you for submitting your clinical trial to PLOS ONE and for providing the name of the registry and the registration number. The information in the registry entry suggests that your trial was registered after patient recruitment began. PLOS ONE strongly encourages authors to register all trials before recruiting the first participant in a study.**

**As per the journal’s editorial policy, please include in the Methods section of your paper:**

**a) your reasons for your delay in registering this study (after enrolment of participants started);**

**b) confirmation that all related trials are registered by stating: “The authors confirm that all ongoing and related trials for this drug/intervention are registered”.**

Response: Thank you for your careful review and for pointing out the concern regarding the timing of trial registration. We acknowledge that this study was registered in the Chinese Clinical Trial Registry (ChiCTR2200065164) on October 30, 2022, after the initiation of participant recruitment (June 13, 2022). This constitutes a retrospective registration.The delay in registration was primarily due to administrative oversight during the early stage of the study initiation. Importantly, the study protocol, including the study design, inclusion/exclusion criteria, primary and secondary outcomes, and statistical analysis plan, had been fully established prior to the enrollment of the first participant and was approved by the Institutional Ethics Committee (approval no: LLSLH20220029). To improve transparency, we have clarified in the revised manuscript that this was a retrospectively registered trial.

Revised text in the Letter:

Line 78-90: This was a prospective, randomized, double-blind, and up-and-down sequential allocation trial. The study protocol was approved by the Institutional Ethics Committee of the Suining Central Hospital (approval no: LLSLH20220029) and registered in the Chinese Clinical Trials Registry in October 30th, 2022 (available at http://www.chictr.org.cn, identifier: ChiCTR2200065164), all methods were carried out in accordance with relevant guidelines and regulations. Participants were recruited between June 13, 2022, and December 31, 2022, from patients at the Suining Central Hospital. The study protocol, including the design, eligibility criteria, primary and secondary outcomes, and statistical analysis plan, was finalized prior to the enrollment of the first participant.The delay in registration was primarily due to administrative oversight during the early stage of the study initiation. The authors confirm that all ongoing and related trials for this drug/intervention are registered and all participants provided their written informed consent prior to participation in the study.

**2. Thank you for stating the following in the Acknowledgments Section of your manuscript:**

**“The author(s) received no specific funding for this work.”**

**We note that you have provided funding information that is not currently declared in your Funding Statement. However, funding information should not appear in the Acknowledgments section or other areas of your manuscript. We will only publish funding information present in the Funding Statement section of the online submission form.**

**Please remove any funding-related text from the manuscript and let us know how you would like to update your Funding Statement. Currently, your Funding Statement reads as follows:**

**“The author(s) received no specific funding for this work.”**

**Please include your amended statements within your cover letter; we will change the online submission form on your behalf.**

Response: Thank you for your valuable comments. We have deleted all funding-related text from the manuscript and have no intention of updating our Funding Statement.

**3. Please review your reference list to ensure that it is complete and correct. If you have cited papers that have been retracted, please include the rationale for doing so in the manuscript text, or remove these references and replace them with relevant current references. Any changes to the reference list should be mentioned in the rebuttal letter that accompanies your revised manuscript. If you need to cite a retracted article, indicate the article’s retracted status in the References list and also include a citation and full reference for the retraction notice.**

Response: Thank you for bringing this to our attention. We acknowledge that Citation 5 has been retracted. The reference has now been removed and replaced with a more appropriate and reliable source (Fredrickson MJ et al., Anaesthesia, 2010). The corresponding citation in the manuscript has been updated accordingly.

Revised text in the Letter:

Line 387-389: Fredrickson MJ, Krishnan S, Chen CY. Postoperative analgesia for shoulder surgery: a critical appraisal and review of current techniques. Anaesthesia. 2010 Jun;65(6):608-624. doi: 10.1111/j.1365-2044.2009.06231.x. PMID: 20565394.

**Response to Reviewer**

**The authors present an analysis of the EC50 obtained using probit regression analysis. However, as noted in their Citation 16 (Pace and Stylianou, 2007), due to the nonindependent and unknown distribution of the sequential allocation method (also noted by the authors at Line 191) this is not recommended. Instead, the recommendation for obtaining the estimate is isotonic regression. Pace and Stylianou recommend the pooled-adjacent-violators algorithm (PAVA). Confidence intervals require the bootstrap method --- in particular, the bootstrap should also be used to determine the confidence interval for the contrast of interest.**

Response:

Thank you for this insightful and important comment. Due to the nonindependent structure and unknown distribution of responses in the up-and-down sequential allocation design, probit regression analysis may not be appropriate, as highlighted by Pace and Stylianou (2007). ^3^In accordance with the reviewer’s suggestion, we have reanalyzed the data using isotonic regression implemented via the pooled-adjacent-violators algorithm (PAVA) to estimate the EC50. Furthermore, bootstrap resampling was applied to obtain robust estimates of the EC50 and corresponding 95% confidence intervals (CIs). In addition, the bootstrap method was used to assess the between-group difference (contrast) in EC50. The bootstrap EC50 of ropivacaine was significantly lower in the butorphanol group (0.24%, 95% CI: 0.23%–0.24%) than in the ropivacaine group (0.30%, 95% CI: 0.29%–0.31%). The confidence intervals did not overlap, indicating a statistically significant difference between the two groups. These findings still confirm that the addition of butorphanol reduces the EC50 of ropivacaine, and the revised analysis provides a more robust and methodologically appropriate estimation.

|  | **Ropivacaine group**  **(n = 18)** | **Butorphanol group (n=22)** | ***P* value** |
| --- | --- | --- | --- |
| PAVA EC50 | 0.31% | 0.24% | *P* < 0.05 |
| Bootstrap EC50 | 0.30% | 0.24% | *P* < 0.05 |
| Probit regression EC50 | 0.31% | 0.24% | *P* < 0.05 |
| Probit regression 95% CI | 0.27%–0.34% | 0.23%–0.25% | *P* < 0.05 |
| 95% CI | 0.29%–0.32% | 0.23%–0.24% | *P* < 0.05 |
| Bootstrap 95% CI | 0.29%–0.31% | 0.23%–0.24% | *P* < 0.05 |

**Also, the authors indicate (Line 214) that patient data was excluded after six crossover points were obtained. At line 152, the authors indicate that the trial would be terminated when seven negative versus positive or vice versa points would be obtained. It is not clear why some patient data were excluded. The authors should clarify this as well as provide a sensitivity analysis including all data to demonstrate that the results are not contingent on this exclusion.**

Response: Thank you for your valuable comments. Upon re-examining the manuscript, we identified an oversight and confirmed that the correct number of crossovers is seven. We sincerely apologize for this inadvertent error. We have now revised the relevant text, tables, and figures throughout the revised manuscript to accurately reflect seven crossovers, ensuring consistency across all sections.

Paul and his colleagues thought that the reliability of the Dixon method increased with the number of pairs and six pairs was enough. By contrast, a further increase in the number of crossovers to 10 affected the average MAC and SD of MAC estimates less. ^1^ To ensure data quality while minimizing the sample size, we selected seven crossovers for our analysis.

**For Table 1 and 2 provide exact p-values instead of "<0.05". Providing exact p-values allows better reproducibility and also allows post hoc p-value adjustment by researchers.**

Response: Thank you for your valuable comments. We have re-examined the statistical analyses and updated all results in Table 1 and Table 2 with the corresponding exact P values. In addition, multiple comparisons were adjusted using the Benjamini–Hochberg (BH) method. The corrected results remain consistent with our original conclusions. Due to formatting requirements (reporting P values to two decimal places), the full precision values were not presented in the original submission. The exact P-values for Table 1 and Table 2 are 0.00006576234,9.12365e^-06^ ,2.577501e^-10^ and 0.0001692.

**The authors have essentially presented a "per protocol" analysis. This is reasonable. However, the authors should also present an "intent to treat" analysis as a sensitivity analysis if at all possible.**

Response: Thank you for your valuable suggestion. We agree that an intention-to-treat analysis, where feasible, provides an important sensitivity assessment in addition to the per-protocol analysis. In response, we re-included the excluded samples and conducted a sensitivity analysis. The sensitivity analysis yielded results that were essentially identical to those of the primary analysis. As presented in Table 5, the PAVA-derived EC50 was 0.30% for the ropivacaine (control) group and 0.239% for the butorphanol (treatment) group. The bootstrap EC50 estimates were 0.302% and 0.238%, respectively. The 95% percentile confidence intervals remained very similar (0.289%–0.319% in the control group and 0.233%–0.240% in the treatment group), confirming that the main findings were robust to re-inclusion of these samples. Accordingly, we have incorporated this sensitivity analysis into the revised manuscript.

|  | **Ropivacaine group**  **(n = 22)** | **Butorphanol group (n=22)** | ***P* value** |
| --- | --- | --- | --- |
| PAVA EC50 | 0.30% | 0.239% | *P* < 0.05 |
| Bootstrap EC50 | 0.302% | 0.238% | *P* < 0.05 |
| 95% CI | 0.289%–0.319% | 0.233%–0.240% | *P* < 0.05 |
| Bootstrap 95% CI | 0.288%–0.314% | 0.225%–0.240% | *P* < 0.05 |

**Line 221: Please provide an industry standard table of adverse reactions.**

Response: Thank you for the valuable comment. Following your suggestion, we compiled detailed information on adverse reactions and supplemented an industry‑standard consolidated table of adverse reactions in the revised manuscript (see Table 2).

**Line 371: It appears that Citation 5 has been retracted.**

Response: Thank you very much for pointing out that a retracted article was included in our references. We have promptly verified its status and confirmed that the paper was retracted on July 12, 2023. At the time of citation, the article had not yet been retracted, and this error represents an unintentional oversight on our part. We sincerely apologize for this issue.

Upon evaluation, we confirmed that this reference was only used for background information and does not affect the core conclusions of our study. Accordingly, we have: (1) removed the retracted article from the reference list and revised the corresponding textby replacing it with authoritative literature; ^2^ (2) Modified the content of lines 61-62 in the manuscript and (3) carefully rechecked all references to ensure that no additional retracted or questionable articles remain.

Thank you again for your careful and rigorous comments. We will strengthen our verification procedures for all references to avoid similar problems in future submissions

**Line 451: In Citation 28 the author name is not complete.**

Response: We sincerely thank the reviewer for pointing out this error. We have corrected the incomplete author name in Citation 28 in the revised manuscript, and ensured that all reference information, including author names, affiliations, and publication details, is complete and consistent throughout the paper.

**Line 463: Include the statistical tests performed here.**

Response: Thank you for the comment. We have now clearly described all statistical tests performed in this analysis in the revised manuscript

**Line 465: In addition to the table it may be of interest to see the data plotted over time.**

Response: Thank you for this valuable suggestion. In accordance with the reviewer’s comment, we have added 3 pictures to visualize the changes of the main data over time in the revised manuscript, alongside the corresponding table, for clearer observation and comparison.

1. Paul M, Fisher DM. Are estimates of MAC reliable? Anesthesiology. 2001 Dec; 95(6):1362-70. doi: 10.1097/00000542-200112000-00014. PMID: 11748393.
2. Fredrickson MJ, Krishnan S, Chen CY. Postoperative analgesia for shoulder surgery: a critical appraisal and review of current techniques. Anaesthesia. 2010 Jun; 65(6):608-624. doi: 10.1111/j.1365-2044.2009.06231.x. PMID: 20565394.
3. Pace NL, Stylianou MP. Advances in and limitations of up-and-down methodology: a précis of clinical use, study design, and dose estimation in anesthesia research. Anesthesiology. 2007;107(1):144-152.
