# Supplementary material for: Butorphanol decreased the median effective concentration of ropivacaine in ultrasound-guided interscalene brachial plexus block
Source: PLoS One. 2026 Jun 16;21(6):e0350613. doi: 10.1371/journal.pone.0350613 (PMC13271508; doi:10.1371/journal.pone.0350613)
Supplement: Peer review history

# Peer review: Decision Letter 0

**DOI:** 10.1371/journal.pone.0350613.r001

**Authors:** José Luiz Fernandes Vieira

18 Feb 2026

PONE-D-25-64915

Butorphanol decreased the median effective concentration of ropivacaine in ultrasound-guided interscalene brachial plexus block

PLOS One

Dear Dr. Tu,

Thank you for submitting your manuscript to PLOS ONE. After careful consideration, we feel that it has merit but does not fully meet PLOS ONE’s publication criteria as it currently stands. Therefore, we invite you to submit a revised version of the manuscript that addresses the points raised during the review process.

==============================

This is an interesting manuscript; however, several revisions are necessary to strengthen the scientific rigor of the study. I recommend that the authors carefully address and incorporate the suggestions provided by the reviewers. This decision is based on PLOS ONE’s publication criteria.We are waiting the changes in your manuscript

Best regards

For Lab, Study and Registered Report Protocols: These article types are not expected to include results but may include pilot data.

==============================

Please submit your revised manuscript by Apr 04 2026 11:59PM.  If you will need more time than this to complete your revisions, please reply to this message or contact the journal office at plosone@plos.org. When you're ready to submit your revision, log on to https://www.editorialmanager.com/pone/ and select the 'Submissions Needing Revision' folder to locate your manuscript file.

Please include the following items when submitting your revised manuscript:

- A letter that responds to each point raised by the academic editor and reviewer(s). You should upload this letter as a separate file labeled 'Response to Reviewers'.
- A marked-up copy of your manuscript that highlights changes made to the original version. You should upload this as a separate file labeled 'Revised Manuscript with Track Changes'.
- An unmarked version of your revised paper without tracked changes. You should upload this as a separate file labeled 'Manuscript'.

-->If you would like to make changes to your financial disclosure, please include your updated statement in your cover letter. Guidelines for resubmitting your figure files are available below the reviewer comments at the end of this letter.

If applicable, we recommend that you deposit your laboratory protocols in protocols.io to enhance the reproducibility of your results. Protocols.io assigns your protocol its own identifier (DOI) so that it can be cited independently in the future. For instructions see: https://journals.plos.org/plosone/s/submission-guidelines#loc-laboratory-protocols. Additionally, PLOS ONE offers an option for publishing peer-reviewed Lab Protocol articles, which describe protocols hosted on protocols.io. Read more information on sharing protocols at https://plos.org/protocols?utm_medium=editorial-email&utm_source=authorletters&utm_campaign=protocols.

We look forward to receiving your revised manuscript.

Kind regards,

José Luiz Fernandes Vieira

Academic Editor

PLOS One

Journal Requirements:

When submitting your revision, we need you to address these additional requirements.

1. Please ensure that your manuscript meets PLOS ONE's style requirements, including those for file naming. The PLOS ONE style templates can be found at

https://journals.plos.org/plosone/s/file?id=wjVg/PLOSOne_formatting_sample_main_body.pdf and

https://journals.plos.org/plosone/s/file?id=ba62/PLOSOne_formatting_sample_title_authors_affiliations.pdf

2. Thank you for submitting your clinical trial to PLOS ONE and for providing the name of the registry and the registration number. The information in the registry entry suggests that your trial was registered after patient recruitment began. PLOS ONE strongly encourages authors to register all trials before recruiting the first participant in a study.

As per the journal’s editorial policy, please include in the Methods section of your paper:

a) your reasons for your delay in registering this study (after enrolment of participants started);

b) confirmation that all related trials are registered by stating: “The authors confirm that all ongoing and related trials for this drug/intervention are registered”.

3. Thank you for stating the following in the Acknowledgments Section of your manuscript:

“The author(s) received no specific funding for this work.”

We note that you have provided funding information that is not currently declared in your Funding Statement. However, funding information should not appear in the Acknowledgments section or other areas of your manuscript. We will only publish funding information present in the Funding Statement section of the online submission form.

Please remove any funding-related text from the manuscript and let us know how you would like to update your Funding Statement. Currently, your Funding Statement reads as follows:

“The author(s) received no specific funding for this work.”

Please include your amended statements within your cover letter; we will change the online submission form on your behalf.

4. If the reviewer comments include a recommendation to cite specific previously published works, please review and evaluate these publications to determine whether they are relevant and should be cited. There is no requirement to cite these works unless the editor has indicated otherwise.

5. Please review your reference list to ensure that it is complete and correct. If you have cited papers that have been retracted, please include the rationale for doing so in the manuscript text, or remove these references and replace them with relevant current references. Any changes to the reference list should be mentioned in the rebuttal letter that accompanies your revised manuscript. If you need to cite a retracted article, indicate the article’s retracted status in the References list and also include a citation and full reference for the retraction notice.

[Note: HTML markup is below. Please do not edit.]

Reviewers' comments:

Reviewer's Responses to Questions

**Comments to the Author**

1. Is the manuscript technically sound, and do the data support the conclusions?

The manuscript must describe a technically sound piece of scientific research with data that supports the conclusions. Experiments must have been conducted rigorously, with appropriate controls, replication, and sample sizes. The conclusions must be drawn appropriately based on the data presented.

Reviewer #1: Partly

**********

2. Has the statistical analysis been performed appropriately and rigorously?

Reviewer #1: No

**********

3. Have the authors made all data underlying the findings in their manuscript fully available?

The PLOS Data policy requires authors to make all data underlying the findings described in their manuscript fully available without restriction, with rare exception (please refer to the Data Availability Statement in the manuscript PDF file). The data should be provided as part of the manuscript or its supporting information, or deposited to a public repository. For example, in addition to summary statistics, the data points behind means, medians and variance measures should be available. If there are restrictions on publicly sharing data—e.g. participant privacy or use of data from a third party—those must be specified.

Reviewer #1: No

**********

4. Is the manuscript presented in an intelligible fashion and written in standard English?

PLOS ONE does not copyedit accepted manuscripts, so the language in submitted articles must be clear, correct, and unambiguous. Any typographical or grammatical errors should be corrected at revision, so please note any specific errors here.

Reviewer #1: Yes

**********

5. Review Comments to the Author

Please use the space provided to explain your answers to the questions above. You may also include additional comments for the author, including concerns about dual publication, research ethics, or publication ethics. (Please upload your review as an attachment if it exceeds 20,000 characters)

Reviewer #1: # General remarks:

The authors present an analysis of the EC50 obtained using probit regression analysis. However, as noted in their Citation 16 (Pace and Stylianou, 2007), due to the nonindependent and unknown distribution of the sequential allocation method (also noted by the authors at Line 191) this is not recommended. Instead, the recommendation for obtaining the estimate is isotonic regression. Pace and Stylianou recommend the pooled-adjacent-violators algorithm (PAVA). Confidence intervals require the bootstrap method --- in particular, the bootstrap should also be used to determine the confidence interval for the contrast of interest.

Also, the authors indicate (Line 214) that patient data was excluded after six crossover points were obtained. At line 152, the authors indicate that the trial would be terminated when seven negative versus positive or vice versa points would be obtained. It is not clear why some patient data were excluded. The authors should clarify this as well as provide a sensitivity analysis including all data to demonstrate that the results are not contingent on this exclusion.

For Table 1 and 2 provide exact p-values instead of "<0.05". Providing exact p-values allows better reproducibility and also allows post hoc p-value adjustment by researchers.

The authors have essentially presented a "per protocol" analysis. This is reasonable. However, the authors should also present an "intent to treat" analysis as a sensitivity analysis if at all possible.

# Specific remarks:

Line 221: Please provide an industry standard table of adverse reactions.

Line 371: It appears that Citation 5 has been retracted.

Line 451: In Citation 28 the author name is not complete.

Line 463: Include the statistical tests performed here.

Line 465: In addition to the table it may be of interest to see the data plotted over time.

**********

6. PLOS authors have the option to publish the peer review history of their article (what does this mean?). If published, this will include your full peer review and any attached files.

If you choose “no”, your identity will remain anonymous but your review may still be made public.

**Do you want your identity to be public for this peer review?**     For information about this choice, including consent withdrawal, please see our Privacy Policy.

Reviewer #1: No

**********

[NOTE: If reviewer comments were submitted as an attachment file, they will be attached to this email and accessible via the submission site. Please log into your account, locate the manuscript record, and check for the action link "View Attachments". If this link does not appear, there are no attachment files.]

To ensure your figures meet our technical requirements, please review our figure guidelines: https://journals.plos.org/plosone/s/figures

You may also use PLOS’s free figure tool, NAAS, to help you prepare publication quality figures: https://journals.plos.org/plosone/s/figures#loc-tools-for-figure-preparation.

NAAS will assess whether your figures meet our technical requirements by comparing each figure against our figure specifications.

# Author comment: Author response to Decision Letter 1

**DOI:** 10.1371/journal.pone.0350613.r002

24 Apr 2026

Response to Reviewer

The authors present an analysis of the EC50 obtained using probit regression analysis. However, as noted in their Citation 16 (Pace and Stylianou, 2007), due to the nonindependent and unknown distribution of the sequential allocation method (also noted by the authors at Line 191) this is not recommended. Instead, the recommendation for obtaining the estimate is isotonic regression. Pace and Stylianou recommend the pooled-adjacent-violators algorithm (PAVA). Confidence intervals require the bootstrap method --- in particular, the bootstrap should also be used to determine the confidence interval for the contrast of interest.

Response:

Thank you for this insightful and important comment. Due to the nonindependent structure and unknown distribution of responses in the up-and-down sequential allocation design, probit regression analysis may not be appropriate, as highlighted by Pace and Stylianou (2007). 3In accordance with the reviewer’s suggestion, we have reanalyzed the data using isotonic regression implemented via the pooled-adjacent-violators algorithm (PAVA) to estimate the EC50. Furthermore, bootstrap resampling was applied to obtain robust estimates of the EC50 and corresponding 95% confidence intervals (CIs). In addition, the bootstrap method was used to assess the between-group difference (contrast) in EC50. The bootstrap EC50 of ropivacaine was significantly lower in the butorphanol group (0.24%, 95% CI: 0.23%–0.24%) than in the ropivacaine group (0.30%, 95% CI: 0.29%–0.31%). The confidence intervals did not overlap, indicating a statistically significant difference between the two groups. These findings still confirm that the addition of butorphanol reduces the EC50 of ropivacaine, and the revised analysis provides a more robust and methodologically appropriate estimation.

Ropivacaine group

(n = 18) Butorphanol group (n=22) P value

PAVA EC50 0.31% 0.24% P < 0.05

Bootstrap EC50 0.30% 0.24% P < 0.05

Probit regression EC50 0.31% 0.24% P < 0.05

Probit regression 95% CI 0.27%–0.34% 0.23%–0.25% P < 0.05

95% CI 0.29%–0.32% 0.23%–0.24% P < 0.05

Bootstrap 95% CI 0.29%–0.31% 0.23%–0.24% P < 0.05

Also, the authors indicate (Line 214) that patient data was excluded after six crossover points were obtained. At line 152, the authors indicate that the trial would be terminated when seven negative versus positive or vice versa points would be obtained. It is not clear why some patient data were excluded. The authors should clarify this as well as provide a sensitivity analysis including all data to demonstrate that the results are not contingent on this exclusion.

Response: Thank you for your valuable comments. Upon re-examining the manuscript, we identified an oversight and confirmed that the correct number of crossovers is seven. We sincerely apologize for this inadvertent error. We have now revised the relevant text, tables, and figures throughout the revised manuscript to accurately reflect seven crossovers, ensuring consistency across all sections.

Paul and his colleagues thought that the reliability of the Dixon method increased with the number of pairs and six pairs was enough. By contrast, a further increase in the number of crossovers to 10 affected the average MAC and SD of MAC estimates less. 1 To ensure data quality while minimizing the sample size, we selected seven crossovers for our analysis.

For Table 1 and 2 provide exact p-values instead of "<0.05". Providing exact p-values allows better reproducibility and also allows post hoc p-value adjustment by researchers.

Response: Thank you for your valuable comments. We have re-examined the statistical analyses and updated all results in Table 1 and Table 2 with the corresponding exact P values. In addition, multiple comparisons were adjusted using the Benjamini–Hochberg (BH) method. The corrected results remain consistent with our original conclusions. Due to formatting requirements (reporting P values to two decimal places), the full precision values were not presented in the original submission. The exact P-values for Table 1 and Table 2 are 0.00006576234,9.12365e-06 ,2.577501e-10 and 0.0001692.

The authors have essentially presented a "per protocol" analysis. This is reasonable. However, the authors should also present an "intent to treat" analysis as a sensitivity analysis if at all possible.

Response: Thank you for your valuable suggestion. We agree that an intention-to-treat analysis, where feasible, provides an important sensitivity assessment in addition to the per-protocol analysis. In response, we re-included the excluded samples and conducted a sensitivity analysis. The sensitivity analysis yielded results that were essentially identical to those of the primary analysis. As presented in Table 5, the PAVA-derived EC50 was 0.30% for the ropivacaine (control) group and 0.239% for the butorphanol (treatment) group. The bootstrap EC50 estimates were 0.302% and 0.238%, respectively. The 95% percentile confidence intervals remained very similar (0.289%–0.319% in the control group and 0.233%–0.240% in the treatment group), confirming that the main findings were robust to re-inclusion of these samples. Accordingly, we have incorporated this sensitivity analysis into the revised manuscript.

Ropivacaine group

(n = 22) Butorphanol group (n=22) P value

PAVA EC50 0.30% 0.239% P < 0.05

Bootstrap EC50 0.302% 0.238% P < 0.05

95% CI 0.289%–0.319% 0.233%–0.240% P < 0.05

Bootstrap 95% CI 0.288%–0.314% 0.225%–0.240% P < 0.05

Line 221: Please provide an industry standard table of adverse reactions.

Response: Thank you for the valuable comment. Following your suggestion, we compiled detailed information on adverse reactions and supplemented an industry‑standard consolidated table of adverse reactions in the revised manuscript (see Table 2).

Line 371: It appears that Citation 5 has been retracted.

Response: Thank you very much for pointing out that a retracted article was included in our references. We have promptly verified its status and confirmed that the paper was retracted on July 12, 2023. At the time of citation, the article had not yet been retracted, and this error represents an unintentional oversight on our part. We sincerely apologize for this issue.

Upon evaluation, we confirmed that this reference was only used for background information and does not affect the core conclusions of our study. Accordingly, we have: (1) removed the retracted article from the reference list and revised the corresponding textby replacing it with authoritative literature; 2 (2) Modified the content of lines 61-62 in the manuscript and (3) carefully rechecked all references to ensure that no additional retracted or questionable articles remain.

Thank you again for your careful and rigorous comments. We will strengthen our verification procedures for all references to avoid similar problems in future submissions

Line 451: In Citation 28 the author name is not complete.

Response: We sincerely thank the reviewer for pointing out this error. We have corrected the incomplete author name in Citation 28 in the revised manuscript, and ensured that all reference information, including author names, affiliations, and publication details, is complete and consistent throughout the paper.

Line 463: Include the statistical tests performed here.

Response: Thank you for the comment. We have now clearly described all statistical tests performed in this analysis in the revised manuscript

Line 465: In addition to the table it may be of interest to see the data plotted over time.

Response: Thank you for this valuable suggestion. In accordance with the reviewer’s comment, we have added 3 pictures to visualize the changes of the main data over time in the revised manuscript, alongside the corresponding table, for clearer observation and comparison.

1. Paul M, Fisher DM. Are estimates of MAC reliable? Anesthesiology. 2001 Dec; 95(6):1362-70. doi: 10.1097/00000542-200112000-00014. PMID: 11748393.

2. Fredrickson MJ, Krishnan S, Chen CY. Postoperative analgesia for shoulder surgery: a critical appraisal and review of current techniques. Anaesthesia. 2010 Jun; 65(6):608-624. doi: 10.1111/j.1365-2044.2009.06231.x. PMID: 20565394.

3. Pace NL, Stylianou MP. Advances in and limitations of up-and-down methodology: a précis of clinical use, study design, and dose estimation in anesthesia research. Anesthesiology. 2007;107(1):144-152.

Attachment Submitted filename: Response to Reviewers.docx

# Peer review: Decision Letter 1

**DOI:** 10.1371/journal.pone.0350613.r003

**Authors:** José Luiz Fernandes Vieira

18 May 2026

<p>Butorphanol decreased the median effective concentration of ropivacaine in ultrasound-guided interscalene brachial plexus block

PONE-D-25-64915R1

Dear Dr. TU

We’re pleased to inform you that your manuscript has been judged scientifically suitable for publication and will be formally accepted for publication once it meets all outstanding technical requirements.

Within one week, you’ll receive an e-mail detailing the required amendments. When these have been addressed, you’ll receive a formal acceptance letter and your manuscript will be scheduled for publication.

An invoice will be generated when your article is formally accepted. Please note, if your institution has a publishing partnership with PLOS and your article meets the relevant criteria, all or part of your publication costs will be covered. Please make sure your user information is up-to-date by logging into Editorial Manager at Editorial Manager® and clicking the ‘Update My Information' link at the top of the page. For questions related to billing, please contact billing support.

If your institution or institutions have a press office, please notify them about your upcoming paper to help maximize its impact. If they’ll be preparing press materials, please inform our press team as soon as possible -- no later than 48 hours after receiving the formal acceptance. Your manuscript will remain under strict press embargo until 2 pm Eastern Time on the date of publication. For more information, please contact onepress@plos.org.

Kind regards,

José Luiz Fernandes Vieira

Academic Editor

PLOS One

Reviewers' comments:

Reviewer's Responses to Questions

**Comments to the Author**

1. If the authors have adequately addressed your comments raised in a previous round of review and you feel that this manuscript is now acceptable for publication, you may indicate that here to bypass the “Comments to the Author” section, enter your conflict of interest statement in the “Confidential to Editor” section, and submit your "Accept" recommendation.

Reviewer #1: All comments have been addressed

**********

2. Is the manuscript technically sound, and do the data support the conclusions?

The manuscript must describe a technically sound piece of scientific research with data that supports the conclusions. Experiments must have been conducted rigorously, with appropriate controls, replication, and sample sizes. The conclusions must be drawn appropriately based on the data presented.

Reviewer #1: (No Response)

**********

3. Has the statistical analysis been performed appropriately and rigorously?

Reviewer #1: (No Response)

**********

4. Have the authors made all data underlying the findings in their manuscript fully available?

The PLOS Data policy requires authors to make all data underlying the findings described in their manuscript fully available without restriction, with rare exception (please refer to the Data Availability Statement in the manuscript PDF file). The data should be provided as part of the manuscript or its supporting information, or deposited to a public repository. For example, in addition to summary statistics, the data points behind means, medians and variance measures should be available. If there are restrictions on publicly sharing data—e.g. participant privacy or use of data from a third party—those must be specified.

Reviewer #1: (No Response)

**********

5. Is the manuscript presented in an intelligible fashion and written in standard English?

PLOS ONE does not copyedit accepted manuscripts, so the language in submitted articles must be clear, correct, and unambiguous. Any typographical or grammatical errors should be corrected at revision, so please note any specific errors here.

Reviewer #1: (No Response)

**********

6. Review Comments to the Author

Please use the space provided to explain your answers to the questions above. You may also include additional comments for the author, including concerns about dual publication, research ethics, or publication ethics. (Please upload your review as an attachment if it exceeds 20,000 characters)

Reviewer #1: (No Response)

**********

7. PLOS authors have the option to publish the peer review history of their article (what does this mean?). If published, this will include your full peer review and any attached files.

If you choose “no”, your identity will remain anonymous but your review may still be made public.

**Do you want your identity to be public for this peer review?**     For information about this choice, including consent withdrawal, please see our Privacy Policy.

Reviewer #1: No

**********

# Editor report: Acceptance letter

**DOI:** 10.1371/journal.pone.0350613.r004

**Authors:** José Luiz Fernandes Vieira

PONE-D-25-64915R1

PLOS One

Dear Dr. Tu,

I'm pleased to inform you that your manuscript has been deemed suitable for publication in PLOS One. Congratulations! Your manuscript is now being handed over to our production team.

At this stage, our production department will prepare your paper for publication. This includes ensuring the following:

* All references, tables, and figures are properly cited

* All relevant supporting information is included in the manuscript submission,

* There are no issues that prevent the paper from being properly typeset

You will receive further instructions from the production team, including instructions on how to review your proof when it is ready. Please keep in mind that we are working through a large volume of accepted articles, so please give us a few days to review your paper and let you know the next and final steps.

Lastly, if your institution or institutions have a press office, please let them know about your upcoming paper now to help maximize its impact. If they'll be preparing press materials, please inform our press team within the next 48 hours. Your manuscript will remain under strict press embargo until 2 pm Eastern Time on the date of publication. For more information, please contact onepress@plos.org.

You will receive an invoice from PLOS for your publication fee after your manuscript has reached the completed accept phase. If you receive an email requesting payment before acceptance or for any other service, this may be a phishing scheme. Learn how to identify phishing emails and protect your accounts at https://explore.plos.org/phishing.

If we can help with anything else, please email us at customercare@plos.org.

Thank you for submitting your work to PLOS ONE and supporting open access.

Kind regards,

PLOS ONE Editorial Office Staff

on behalf of

Dr. José Luiz Fernandes Vieira

Academic Editor

PLOS One
